# Public knowledge of ultra-processed foods: Insights from a national survey of Australian adults

**DOI:** 10.1186/s12937-026-01345-3

**Published:** 2026-06-05

**Authors:** Priscila Machado, Emily Denniss, Catherine G. Russell, Neha Khandpur, Jennifer Lacy-Nichols, Cherie Russell, Kamila Gabe, Sarah A. McNaughton

**Affiliations:** 1 Institute for Physical Activity and Nutrition, Deakin University, Geelong, Australia; 2 School of Exercise and Nutrition Sciences, Deakin University, Geelong, Australia; 3 School of Health and Social Development, Deakin University, Geelong, Australia; 4 Division of Human Nutrition and Health, Wageningen University, Wageningen, Netherlands; 5 Center for Epidemiological Research in Nutrition and Health, University of Sao Paulo, Sao Paulo, Brazil; 6 Department of Nutrition, Harvard T. H. Chan School of Public Health, Boston, USA; 7 Centre for Health Equity, Melbourne School of Population and Global Health, University of Melbourne, Melbourne, Australia; 8 Global Centre for Preventive Health and Nutrition, Institute for Health Transformation, School of Medicine, Deakin University, Geelong, Australia; 9 Health and Well-Being Centre for Research Innovation, School of Human Movement and Nutrition Sciences, University of Queensland, St Lucia, Australia

**Keywords:** Food processing, Literacy, Knowledge, Awareness, Adults, Nova system

## Abstract

**Background:**

Ultra-processed foods (UPFs) are increasingly recognised as major contributors to diet-related chronic diseases, environmental deterioration and cultural impacts. While the salience of the UPF concept in societal discourse has also grown substantially in recent years, public knowledge of UPFs remains unclear. This study aimed to explore public knowledge of, and attributes associated with, UPFs in Australia.

**Methods:**

Data included cross-sectional national survey involving 1,086 adults (20–64 years) living in Australia. Data collection occurred between June and July 2024 using a piloted questionnaire administered via Qualtrics market research services. Sociodemographic characteristics and prior awareness about UPFs were collected. The survey included open-ended questions, free-listing tasks, UPF functional knowledge measures (e.g., identification of UPFs among 20 images of products; Food Processing Knowledge (FoodProK) Score), and triadic sorting techniques (used to identify attributes associated with UPFs). Multivariable linear regression models were fitted to examine associations between the number of items correctly identified as UPFs, and FoodProK scores, with socio-demographic characteristics. Open-ended responses to the main survey question ‘*What comes to your mind when you think of the term 'ultra-processed foods'* were analysed using Leximancer, a content analysis software, to identify dominant concepts and themes. Attributes reported through the triad sorting techniques were thematically analysed.

**Results:**

Sixty-four percent of participants reported awareness of the UPF term. The dominant concepts and themes in participants’ responses reflected key characteristics of UPFs (e.g., presence of industrial ingredients) and main examples were fast-food meals and soft drinks. Most participants (85%) were able to correctly identify non-UPFs, and 45% were able to correctly identify UPFs, with multigrain bread and flavoured yoghurt misclassified most often. Socio-demographic differences were observed, with women, people with higher education, Non-Indigenous people and those with prior awareness about UPFs being more likely to correctly identify UPFs. Attributes associated with UPFs included presence of artificial ingredients, low nutritional quality, convenience, and affordability.

**Conclusions:**

In this national survey, most participants were aware of the concept of UPFs and could identify key UPF characteristics, however some UPF items were often misclassified. Future research could explore how UPF knowledge influences diet, the factors shaping this knowledge, and interventions and policies to improve knowledge and reduce UPF consumption.

**Supplementary Information:**

The online version contains supplementary material available at 10.1186/s12937-026-01345-3.

## Background

Ultra-processed foods (UPFs) are defined within the Nova system as formulations of ingredients, mostly of exclusive industrial use, that result from a series of industrial processes [1]. UPFs are highly profitable (low-cost ingredients, long shelf-life, branded), ready-to-eat, palatable, affordable, heavily promoted, and conveniently packaged products liable to displace all other NOVA food groups [2]. These formulations of ingredients (e.g., sugar, oils, protein isolates) typically contain additives like flavours, colours, and emulsifiers, and contaminants, but few whole foods [2]. UPFs include fast foods, soft drinks, snacks, ready-to-heat meals, mass-produced breads, flavoured milk drinks and breakfast cereals [2]. UPFs are now the main source of dietary energy in many high-income countries like Canada, UK and US [3], and their purchases are rapidly growing in several low- and middle-income countries, including highly populated countries such as Indonesia and China [4]. UPFs comprise 42% of total dietary energy in Australia [5], with higher intakes among young people (children, adolescents, adults aged 19-30y) [6, 7] and those with lower incomes and education levels [6]. Dietary patterns characterised by high consumption of ultra-processed foods are associated with adverse health outcomes, including type-2 diabetes, depression, heart diseases, and premature death [8]. Increased UPF production and consumption has also been suggested to result in environmental deterioration, loss of culinary traditions (‘acculturation’), and negative economic impacts on small- and middle-sized food businesses that are pushed out of the market by large-scale UPF manufacturers with substantial capital and power [9, 10].

The Nova system is the food classification system most applied in the scientific literature and policy frameworks when examining the level of industrial processing [11]. The salience of the UPF concept in societal discourse has also grown substantially in recent years, demonstrated by its increased media coverage (e.g., Google News articles on UPFs increased from 8680 in 2021 to 49,600 in 2024 [12]). Nevertheless, analysis of the UPFs coverage in the Australian news media shows that despite its increased attention, UPFs are frequently reported incorrectly [13]. UPFs are often defined as ‘junk foods’ (e.g. soft drinks, chips), but many UPFs are in food categories not typically seen as ‘junk’ (e.g. flavoured yoghurt, packaged bread) [14], which may lead to potential public confusion about UPFs [13, 15]. Alongside this, the public is repeatedly exposed to inconsistent or misleading messages driven by the UPF industry, which seeks to present its products as appealing and healthy (i.e. ‘health-washing’) through aggressive marketing [16, 17] and by shaping nutrition scientific knowledge (e.g., by funding nutrition research to obscure public health evidence and disseminating data that favours industry) [18]. Research shows that UPF industry groups leverage their media connections to promote favourable narratives [19], by disseminating a narrow subset of nutrition information consistent with corporate interests [20].

There is limited independent research on the public’s understanding of UPFs to date. Existing studies are mostly from South America (e.g., Brazil, Uruguay and Ecuador) [21–24], countries that have issued national dietary guidelines using the Nova system, and thus increased UPF awareness in these countries is expected. In those studies, and more recent ones, including in Australia, it was found that the public often associated UPFs with the presence of industrial ingredients (e.g., additives) and negative impacts on health, but the public still faces challenges distinguishing UPF from other types of processed foods [21–25].

The extent to which these findings apply to other contexts, such as Australia, and across different socio-demographic groups remains unclear. Evidence shows that nutrition literacy varies across population groups, with higher levels of nutrition knowledge observed among women, older adults, those experiencing the least disadvantage, and nutrition experts [26–30]. People with lower health or nutrition literacy have been shown to be more susceptible to misinformation and less critical of food advertising [31, 32], often holding perceptions that align more closely with popular media and marketing content rather than with public health guidance [29]. Nutrition literacy is an important predictor of behaviour change, diet quality and health outcomes [33–35]. While most of the evidence on nutrition literacy assessments do not specifically address UPFs, emerging evidence suggests that increased UPF knowledge is associated with reduced consumption [36].

While UPF consumption is influenced by individual determinants, broader environmental and systemic drivers play a critical role in determining its consumption [4, 37]. The global and complex nature of *ultra-processed food systems* require coordinated policy and regulatory actions to transform food systems, supporting efforts to reduce UPF consumption and mitigate its associated harms [38, 39]. Public support can increase the salience of policy issues and the likelihood of adoption [40, 41]; therefore, greater awareness of UPFs may strengthen political will, governments’ capacity to counter industry opposition, and guide the prioritisation of policies targeting UPFs, including effective policies that address harmful commercial practices [18, 39].

Evidence of public understanding about UPFs in Australia can inform the development of more nuanced and tailored interventions and communication strategies to help improve consumer literacy of UPFs, important for both reducing consumption of UPFs and supporting policies to address their production and consumption. This was identified by Australian experts as a priority to advance UPF research and policy in the country [42], as well as internationally [43]. Therefore, this study aimed to explore public knowledge of, and attributes associated with, UPFs in Australia.

## Material and methods

### Study design and participants

This study applied mixed methods to comprehensively assess public knowledge of, and attributes associated with, UPFs. A cross-sectional online survey using quota-based sampling to approximate national population proportions of Australian adults was used. The survey included both open- and close-ended questions and was developed with a diverse panel of consumers, and was piloted with a convenience sample of Australian adults (see details of the pilot study in supplementary file 1).

Participants were eligible if they were aged 20-64y, living in Australia at the time of the survey, and had access to the internet to complete the online survey (either via phone or computer). Data collection occurred between June and July 2024, via Qualtrics market research services. Strategic sampling ensured proportional representation of the Australian population based on age, gender, geographic location (states and territories, and rurality), education, and ethnic background (sample size calculated as n = 1,086 considering margin of error of ± 3.0% at the 95% confidence level based on 2021 census data) [44, 45]. The survey developed in Qualtrics can be found in Supplementary file 2.

This study received ethics approval from the Deakin University Human Ethics Advisory Group (HEAG-H 178_2023; HEAG-H 63_2024). Participation was anonymous and voluntary, and all participants provided informed consent before taking part. This study is reported according to the Good Reporting of a Mixed Methods Study (GRAMMS) checklist (Supplementary file 3).

## Study measures

### Participant characteristics

Socio-demographic characteristics were collected, including age, gender, education, postcode, country of birth, language spoken at home, and identification as Aboriginal, Torres Strait Islander or both. Postcodes were used to identify location (states and territories), to assess residential area (rurality) by matching postcodes to the Australian Bureau of Statistics 2021 Australian Statistical Geography Standard Remoteness Structure [46], and assess area-level disadvantage (Socio-Economic Indexes for Areas, SEIFA) by matching postcodes to the Australian Bureau of Statistics 2021 Index of Relative Socio-Economic Disadvantaged. Other participant characteristics collected included information about professional background in health (yes/no), confidence to communicate in English (yes/no), and prior awareness about UPFs (yes/no).

### UPFs knowledge

In this study, we draw on food literacy literature [47] to define UPF knowledge as encompassing both awareness (i.e., having the information) and understanding of food and food issues. To reflect this multidimensional concept, we employed multiple methods to assess UPF knowledge. Participants were asked to complete open-ended questions (e.g., “*What comes to your mind when you think of the term 'ultra-processed foods'? How would you describe this term?”, “In your view, is there a difference between ultra-processed foods and other types of processed foods? If so, how?”)* and a free-listing task, e.g., “Please list up to 10 different types of foods and drinks that you consider to be ultra-processed”.

We then used an UPF identification task and the Food Processing Knowledge (FoodProK) Score method to measure UPF functional knowledge (i.e., the application of knowledge in action) [47]. Pictures of twenty products (10 UPF and 10 non-UPF, see Supplementary file 2) were presented and participants were asked to select UPFs from this list. The images were chosen as being representative of the most commonly consumed UPFs and non-UPFs among different age groups in the Australian population [5], as well as based on the results of a pilot study. The number of products (in)correctly identified by each participant was calculated. Scores ranged from 0 to 20, reflecting correct classification of both UPF items (n = 10) and non-UPF items (n = 10). An UPF item that was not selected was counted as ‘incorrectly’ identified. Similarly, a non-UPF item that was selected as UPF was counted as incorrectly identified.

Participants rated the healthiness of non-UPFs and UPFs using the FoodProK method [48]. In summary, the FoodProK has been developed as a functional test of nutrition knowledge based on level of processing. In this method, participants view and rate images of products according to their healthiness [48]. In our study, respondents viewed and rated images of two food products within each of four categories, including one non-UPF and one UPF version of following items: fruits (apple and UPF apple fruit drink), meat (chicken breast and UPF chicken nuggets), dairy (milk and UPF cheese slices), and grains (oats and UPF cereal bar). For each of the 8 products, respondents were asked, “Overall, how healthy is this food product?” and answered using a scale of 0 to 10, with 0 representing ‘not healthy at all’ to 10 indicating ‘extremely healthy.’ A food processing knowledge score was calculated by summing the difference between the participant-assigned processing levels of products within each category (i.e., healthiness rating for the non-UPF item minus healthiness rating for the UPF item). For example, if the apple was rated nine and the apple fruit drink, five, then the score for the fruit category was four. Therefore, the total food processing knowledge score ranges from –40 to + 40, with a lower score indicating a lower food processing knowledge.

Finally, a triadic sorting technique [49] was used to identify attributes (constructs) that participants associate with UPFs. In this technique, participants were shown four sets of three food images each. In every set, two UPFs were positioned at the top and one non-UPF at the bottom, forming a triad. Participants were then asked to freely describe the differences they perceived between the UPFs (top images) and the non-UPF (bottom image).

## Data analysis

### Quantitative data

Descriptive analyses were used to summarise participants’ characteristics, proportion of products (in)correctly identified as UPFs and non-UPFs by participants, and FoodProK score. Variables were categorised as gender: women, men and non-binary/other; age: 20–29, 30–39, 40–49, 50–64 years; education: Year 12 or equivalent, Year 11 or lower; SEIFA: quintiles ranging from the least disadvantaged (quintile 1) to the most disadvantaged (quintile 5); country of birth: Australia, other; Aboriginal, Torres Strait Islander or both: Yes/No; Rurality: Major city of Australia, other.

Multivariable (adjusted for all socio-demographic) linear regression models were fitted to examine both associations of the number of food items correctly identified (ranges 0–20, includes both correct identification of UPF and non-UPFs) and FoodProK scores with sociodemographic characteristics. Pairwise comparisons following regression models were adjusted for multiple comparisons using the Bonferroni correction. All statistical analyses were performed using Stata SE version 18. All tests were two-sided and a P < 0.05 was considered statistically significant.

### Qualitative data

Responses to the open-ended question *“What comes to your mind when you think of the term 'ultra-processed foods'? How would you describe this term?”* were content analysed using Leximancer Version 5.5. Leximancer is a software that uses machine learning to content analyse textual data and has been shown to be a reliable and valid content analysis tool [50]. The software identifies “concepts”, which are groups of words that typically appear together throughout the text. A concept map is generated by Leximancer, which visually depicts the frequency of concepts and the relationship between concepts, where concepts that co-occur frequently (i.e., are highly related) are situated close together on the map. Concepts are depicted in grey circles, where the size of the circle corresponds with the frequency of the concept’s occurrence in the text. Leximancer also identifies “themes” which are highly related groups of concepts. Themes are depicted as coloured circles that capture groups of concepts on the map.

Before analysis, spelling mistakes in the open-ended responses were corrected and consistent spelling of wording was applied. Responses for all participants were entered into Leximancer and an exploratory concept map was generated to inform the text processing settings [51, 52]. Leximancer default settings were used, except for the ‘name-like concepts’ and ‘break at paragraph’ settings, which were turned off. It is recommended that Leximancer default settings are used unless there is a good rationale for changing them [51]. Following a similar approach to Denniss et al. [52], ‘name-like concepts’ was turned off because participants did not consistently capitalise proper nouns and the ‘break at paragraph’ feature was removed because participants tended to list words or short sentences followed by a paragraph break. The concepts “ultra-processed”, “food” and “foods” were switched off, so they were not visible in the concept map because these concepts distorted the map. Concepts that sat very close together on the map that were similar or used in the same context were combined (e.g., “meat” and “meats”, “shelf” and “life”).

The sizes of themes can be altered in Leximancer, where a larger theme setting captures more concepts, and conversely, a smaller theme setting captures fewer. Themes at various sizes and their associated text segments were initially explored and interpreted by ED. Two researchers (PM and ED) met to further explore and discuss the themes and their meaning and come to an agreement regarding theme sizes and their interpretation. This process was repeated with open-ended responses stratified by gender (women and men) and level of socio-economic disadvantage (highest and lowest quintile of disadvantage using SEIFA).

NVivo qualitative analysis software (QSR International Pty Ltd, 2018) was used to generate the word cloud for the free-listing task (set to display 100 most frequent, grouping: with stemmed words) and for the thematic analysis of the triad sorting techniques used to identify attributes that participants associate with UPFs. An Excel spreadsheet was used to determine the proportion of participants that reported differences between UPF and other types of processed foods (Yes: there is a difference; No: there is no difference; Unsure: do not know if there is a difference). Reasons provided by participants who answered “Yes” (i.e., how they differ) were coded in NVivo and content analysed.

Once data analysis was complete, authors reviewed and discussed qualitative and quantitative components for an integrated assessment. Following data analysis, the authors reviewed the qualitative and quantitative findings to enable an integrated interpretation of the results.

## Results

### Participant characteristics

A total of 1,086 participants completed the survey (Table 1), with a mean (SD) survey completion time of 15.5 (17.5) minutes. Among them, 51.6% were women, 83.1% had completed Year 12 or an equivalent level of education, and the majority were born in Australia (76.9%). Almost all participants spoke English at home (94%) and did not identify as Aboriginal, Torres Strait Islander or both (96.1%). Sixty-four percent reported awareness about UPFs prior to the survey.

**Table 1:** Characteristics of the study participants (n = 1,086)

Characteristic	Number	%
Gender		
Women	560	51.6
Men	509	46.9
Non-binary or other	17	1.5
Age (years)		
20–29	274	25.2
30–39	284	26.2
40–49	255	23.5
50–64	273	25.1
Education		
Year 12 or equivalent	902	83.1
Year 11 or lower	184	16.9
Area-level disadvantage		
First quintile (greater disadvantage)	200	18.5
Second quintile	188	17.4
Third quintile	257	23.7
Fourth quintile	246	22.7
Fifth quintile (most advantage)	191	17.6
Country of birth		
Australia	824	76.9
United Kingdom	44	4.1
India	25	2.3
Other	178	16.6
Language spoken at home		
English	1,009	94.0
Hindi	13	1.2
Cantonese	12	1.1
Other	40	3.7
Aboriginal, Torres Strait Islander or both		
Yes	42	3.9
No	1,044	96.1
State		
New South Wales	346	31.8
Victoria	289	26.6
Queensland	218	20.1
South Australia	94	8.6
Western Australia	89	8.2
Tasmania	30	2.8
Australian Capital Territory	16	1.5
Northern Territory	4	0.4
Rurality		
Major city of Australia	853	78.8
Inner regional of Australia	160	14.8
Other	71	6.4
Professional Health background		
Yes	162	14.9
No	924	85.1
Prior awareness about UPFs		
Yes	695	64.0
No	391	36.0

## Quantitative data

### Correct UPF identification, FoodProK scores and associations with socio-demographic characteristics

**Fig. 1:**
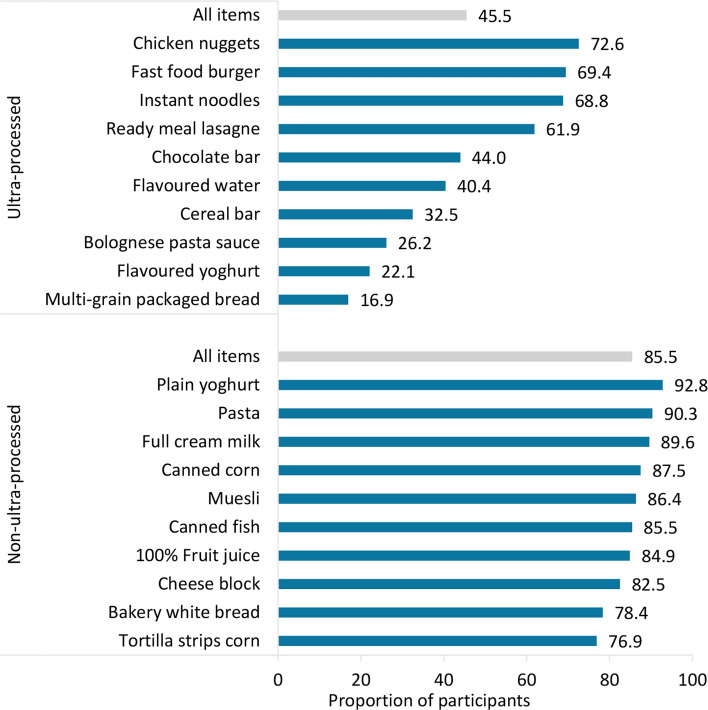
Proportion of participants who correctly identified ultra-processed food items and non-ultra-processed food items

Overall, UPFs were correctly identified by 45.5% and non-UPFs by 85.5% of participants, with a mean ± SD of total number of items correctly identified of 13.1 ± 2.4 (Fig. 1, supplementary file 5). Within the UPF group, chicken nuggets (72.6%), fast food burger (69.4%) and instant noodles (68.8%) were the items that were most often correctly identified, whereas multi-grain packaged bread (16.9%), flavoured yoghurt (22.1%) and pasta sauce (26.2%) were less often correctly identified. Within the non-UPF group, plain yoghurt (92.8%), pasta (90.3%) and full cream milk (89.6%) were most often correctly identified. Tortilla strips and bakery white bread were incorrectly identified as UPFs about 20% of the time.

In this study, the mean (SD) FoodProK score was 17.21 (± 8.53) (Supplementary file 6). Table 2 presents the association between the number of items correctly identified as UPFs and FoodProK scoring and sociodemographic characteristics of the population. A significantly lower number of correctly identified foods was observed for men (β = −0.79; 95%CI −1.08, −0.50), those of lower education (β = −0.41; 95%CI −0.81, −0.02), among Aboriginal and/or Torres Strait Islander (β = −0.80; 95%CI −1.56, −0.04), and those who reported no prior awareness about UPFs (β = −0.99; 95%CI −1.28, −0.70); and higher in Tasmania (β = 1.83; 95%CI 0.90, 2.76) and among those without a professional health background (β = 0.66; 95%CI 0.25, 1.06) (see multiple comparisons in Supplementary file 7).

**Table 2:** Associations of number of UPFs correctly identified as UPFs and Food Processing Knowledge (FoodProK) scores with socio-demographic characteristics (*n* = 1,086)

Characteristic	Number of items correctly identified as UPFs*			FoodProK**		
	Mean (SD)	Adjusted β (95% CI)	*P*-value	Mean (SD)	Adjusted β (95% CI)	*P*-value
Gender			** < 0.001**			** < 0.001**
Women	13.5 ± 2.3	Ref		18.7 ± 8.3	Ref	
Men	12.7 ± 2.4	−0.79 (−1.08, −0.50)		15.0 ± 8.4	−4.18 (−5.22, −3.13)	
Non-binary/other	13.5 ± 2.3	0.10 (−1.04, 1.25)		20.8 ± 6.5	2.73 (−1.68, 7.13)	
Age (years)			0.641			**0.002**
20–29	13.1 ± 2.3	Ref		15.7 ± 8.6	Ref	
30–39	13.1 ± 2.4	0.04 (−0.37, 0.44)		17.1 ± 8.5	1.15 (−0.30, 2.60)	
40–49	13.3 ± 2.4	0.30 (−0.11, 0.72)		17.9 ± 8.6	2.32 (0.84, 3.80)	
50–64	13.0 ± 2.4	0.14 (−0.27, 0.56)		17.7 ± 8.3	2.71 (1.22, 4.20)	
Education			**0.033**			0.443
Year 12 or equivalent	13.2 ± 2.4	Ref		17.1 ± 8.5	Ref	
Year 11 or lower	12.8 ± 2.4	−0.41 (−0.81, −0.02)		17.0 ± 8.7	−0.65 (−2.06, 0.76)	
Area-level disadvantage			**0.022**			**0.044**
First quintile	13.0 ± 2.4	Ref		16.7 ± 8.2	Ref	
Second quintile	13.0 ± 2.5	−0.07 (−0.55, 0.41)		16.7 ± 8.9	−0.49 (−2.21, 1.22)	
Third quintile	13.0 ± 2.4	−0.06 (−0.051, 0.34)		16.4 ± 9.0	−0.44 (−2.06, 1.17)	
Fourth quintile	13.2 ± 2.4	0.11 (−0.35, 0.58)		17.6 ± 8.4	0.81 (−0.86, 2.48)	
Fifth quintile	13.5 ± 2.2	0.48 (−0.01, 0.98)		18.1 ± 7.8	1.22 (−0.58, 3.01)	
Country of birth			0.122			0.389
Australia	13.2 ± 2.4	Ref		17.3 ± 8.6	Ref	
Other	12.9 ± 2.3	−0.28 (0.65, 0.09)		16.4 ± 8.3	−0.61 (−1.93, 0.71)	
Language spoken			0.192			0.090
English	13.2 ± 2.4	Ref		17.2 ± 8.6	Ref	
Other	12.6 ± 2.4	−0.44 (−1.09, 0.20)		14.8 ± 7.7	−1.87 (−4.19, 0.44)	
Aboriginal, Torres Strait Islander or both			**0.047**			** < 0.001**
No	12.2 ± 2.9	Ref		17.3 ± 8.4	Ref	
Yes	13.2 ± 2.4	−0.80 (−1.56, −0.04)		11.5 ± 10.4	−5.46 (−8.27, −2.64)	
State			0.065			0.390
New South Wales	12.9 ± 2.4	Ref		17.3 ± 8.3	Ref	
Victoria	13.3 ± 2.5	0.37 (0.002, 0.75)		17.5 ± 8.4	0.35 (−0.98, 1.70)	
Queensland	13.0 ± 2.4	0.15 (−0.25, 0.55)		16.1 ± 9.6	−1.21 (−2.67, 0.24)	
South Australia	12.9 ± 2.3	0.005 (−0.54, 0.55)		17.1 ± 8.0	−0.10(−2.05, 1.87)	
Western Australia	13.1 ± 2.0	0.14 (−0.41, 0.70)		17.0 ± 6.7	−0.21 (−2.18, 1.76)	
Tasmania	14.5 ± 2.1	1.83 (0.90, 2.76)		16.4 ± 7.9	−1.42 (−4.79, 1.94)	
Australian Capital Territory	13.4 ± 2.6	0.21 (−1.01, 1.44)		16.7 ± 10.1	−1.02 (−5.62, 3.58)	
Northern Territory	14.0 ± 3.6	1.10 (−1.25, 3.44)		20.2 ± 12.5	2.92 (−5.28, 11.14)	
Rurality			0.697			0.237
Major city of Australia	13.1 ± 2.4	Ref		16.9 ± 8.5	Ref	
Other	13.1 ± 2.4	−0.11 (−0.50, 0.28)		17.6 ± 8.6	−1.01 (−2.42, 0.40)	
Health background			**0.002**			**0.001**
Yes	12.7 ± 2.4	Ref		14.9 ± 10.0	Ref	
No	13.2 ± 2.4	0.66 (0.25, 1.06)		17.5 ± 8.2	2.39 (0.94, 3.84)	
Prior awareness about UPFs			** < 0.001**			** < 0.001**
Yes	13.5 ± 2.4	Ref		17.8 ± 8.4	Ref	
No	12.5 ± 2.3	−0.99 (−1.28, −0.70)		15.7 ± 8.5	−2.24 (−3.30, −1.17)	

^*^Ranges from 0–20, includes correctly classifying both UPF (n = 10) and non-UPF (n = 10) items; **Ranges from –40 to + 40, with a lower score indicating a lower food processing knowledge. *P*-values <0.05 indicate statistical significance and are shown in bold

FoodProK scores (lower score indicating a lower food processing knowledge) were also significantly lower for men (β = −4.18; 95%CI −5.22, −3.13), among Aboriginal, Torres Strait Islander/both (β = −5.46; 95%CI −8.27, −2.64) and those without prior awareness about UPFs (β = −2.24; 95%CI −3.30, −1.17); and higher in people aged 40–49 vs. 20–29 years (β = 2.32; 95%CI 0.84, 3.80) and those without a professional health background (β = 2.39; 95%CI 0.94, 3.84) (Table 2; see multiple comparisons in Supplementary file 7).

## Qualitative data

### Participant descriptions of UPFs

**Fig. 2:**
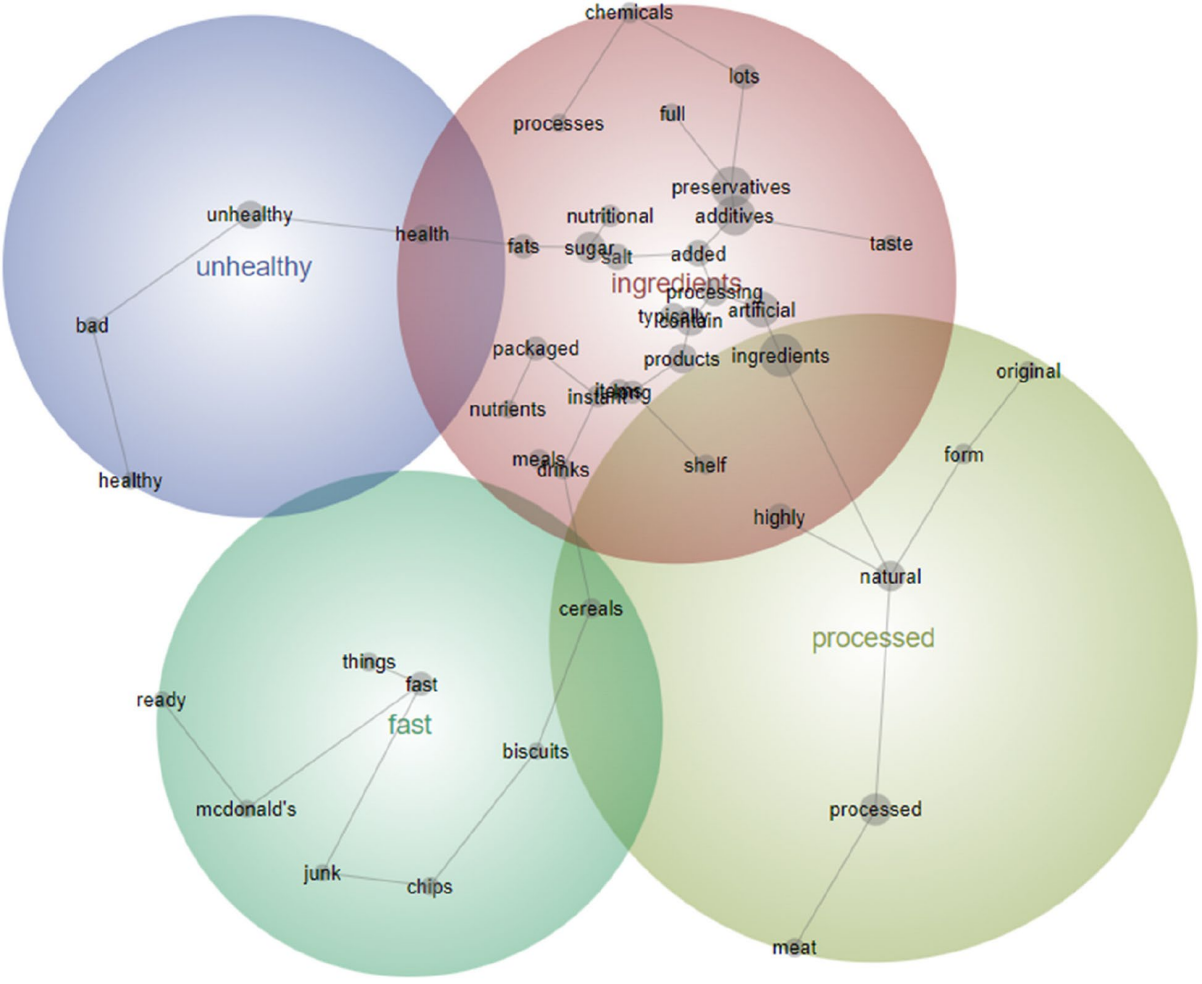
Leximancer concept map for all participant responses describing ultra-processed foods. Legend: In decreasing order of dominance in the text themes were: “ingredients”, “processed”, “fast” and “unhealthy”. The large, coloured circles depict the themes and are heat mapped, whereby warmer colours depict more dominant themes. The small grey circles depict concepts and the circle size represents the frequency of appearance in the text, whereby larger circles depict greater frequency. The proximity of concepts on the map represents the relationship between concepts and their co-occurrence in the text, where closer proximity indicates more frequent co-occurrence

The Leximancer concept map generated from the open-ended responses from all participants is shown in Fig. 2. At a theme size of 60%, four themes and 44 concepts were identified. In order of most to least dominant, the themes were: “ingredients”, “processed”, “fast” and “unhealthy”.

Themes and the frequency and relevance of each identified concept are presented in Supplementary file 8 and illustrative quotes for each theme are presented in Table 3.

**Table 3:** Illustrative quotes of participant descriptions of ultra-processed foods under each Leximancer identified theme

Theme	Illustrative quote
Ingredients	*“…food products that have undergone extensive processing and contain additives, preservatives, flavour enhancers, and often high levels of sugars, fats, and salts.”* – Man, 34 years *“Foods full of nasties like artificial preservatives, monosodium glutamate, artificial colours and artificial food additives. When you read the ingredient labels, there are a lot of food numbers (or scientific sounding names).”* – Woman, 56 years *“They’re loaded with ingredients like preservatives, artificial colours and flavours, emulsifiers, and sweeteners to enhance taste, texture, and shelf life.”* – Man, 36 years *“…often contain ingredients not commonly used in home cooking…”* – Woman, 26 years
Processed	*“Food that has been highly modified from its original form.”* – Man, 44 years *“Anything which a normal unprofessional human cannot trace back the origin of the food like bacon, sausage. We do know these are meat based but it’s not easy to differentiate what meat it is.”* – Woman, 28 years *“Foods that aren't natural in any way shape or form.”* – Man, 26 years *“…not real genuine foods but processed in a factory or man-made.”* – Woman, 22 years
Fast	*“Junk food such as fast food restaurant burgers, ice cream, chicken nuggets, tenders, freezer finger foods, soups and biscuits.”* – Woman, 28 years *“Packaged snacks like chips, chocolates, cereals and lollies.”* – Woman, 20 years *“McDonald’s, fast food, ready made pizza, tinned spaghetti”* – Man, 61 years *“Takeaway or ready to eat meals from supermarket”* – Man, 44 years
Unhealthy	*“Negative, bad for health, chemicals, no nutrients”* – Woman, 22 years *“Bad for you.”* – Woman, 40 years *“…very bad and unhealthy food…”* – Man, 28 years *“Food that is healthy and delicious.”* – Woman, 28 years

When concept maps were stratified by gender, the two most dominant themes “ingredients” and “processed” and the content of participant responses linked to each theme were consistent with the unstratified Leximancer output. The concept map generated using responses from men automatically assigned the name “preservatives” to the most dominant theme. However, upon inspection, the content of this theme related to ingredients, which was the third most frequent concept for the theme, so it was renamed as “ingredients”. A key difference between women and men’s descriptions of UPFs was the idea of UPFs being unhealthy. While this was mentioned by both genders and concepts related to health appeared in both concept maps, it was more frequently mentioned by women, evidenced by “unhealthy” and “healthy” being the third and fourth most dominant themes for women. Women’s responses under the “unhealthy” and “healthy” themes described UPFs as unhealthy, fake and bad for health. Text segments under the third and fourth most dominant themes for men both described examples of UPFs and these themes were named “chips” and “things” (e.g., “*I think of things like McDonald’s*”). Concept maps for responses stratified by gender are presented in Supplementary File 4. The two most dominant themes identified in the unstratified concept map (“ingredients” and “processing”) were consistent in the stratified analysis by level of socio-economic disadvantage (highest and lowest quintile). However, the number of responses captured in each concept map were insufficient to draw meaning from the less dominant themes, due to the small number of responses captured under each theme (data not shown). Results from the unstratified Leximancer analysis are described in detail below.

### Theme 1: Ingredients

The theme “ingredients” had the highest ranked importance and had a total of 490 linked text segments (hits). The concepts under this theme that occurred most often in participant responses were “ingredients”, “preservatives”, “additives”, “artificial”, and “sugar”. Linked text segments described UPFs as foods that have a long list of ingredients and additives. Participants outlined ingredients such as preservatives, colours, flavours, emulsifiers, and sweeteners and such ingredients were often described as “artificial”, “fake”, or “chemicals”. Furthermore, responses commonly mentioned that UPFs typically contain low levels of vitamins, minerals, and fibre and large amounts of added salt, sugar, and fat, resulting in them being nutritionally unbalanced or having “low nutritional value”. Participants also commonly described the outcomes of processing and the addition of ingredients on other characteristics of UPFs, including enhanced palatability, shelf life, texture and appearance.

### Theme 2: Processed

The theme “processed” was the second most dominant theme ranked by Leximancer, with a total of 320 hits. The most frequently counted concepts were “processed”, “natural” and “meat”. Text segments under this theme described UPFs as foods that have undergone extensive processing resulting in them being far from their “natural” or “original” form. Participants described UPFs as being so far removed from their natural state that it is difficult to identify the main ingredients. It was also common for participants to mention that UPFs are man-made in factories and “not natural”. Processed meat was frequently given as an example of UPFs, and some text segments mentioned that UPFs are unhealthy or have minimal nutritional value.

### Theme 3: Fast

The theme “fast” was ranked third in importance by Leximancer and had 194 hits. “Fast”, “things”, “chips” and “junk” were the most frequently counted concepts under this theme. Text segments described UPFs as “junk foods” and examples of UPFs were frequently provided by participants. Foods such as fast food, McDonald’s, confectionery, soft drink, chips, processed meats and frozen foods were common examples. Participants also frequently described UPFs as ready-to-eat foods and foods that came in packages.

### Theme 4: Unhealthy

The final theme was “unhealthy” with a total of 182 linked text segments. The most frequently counted concepts were “unhealthy” and “healthy” (most often used in the context of “not healthy”). Participant responses under this theme frequently described UPFs as “unhealthy” or “bad for health”. Descriptions of UPFs being unhealthy tended to be general and only six of the 182 text segments under this theme mentioned specific health outcomes (cancer, cardiovascular disease, poor gut health, obesity, diabetes and low energy). One response mentioned level of UPF consumption when discussing health. Conversely, of the 182 linked text segments, seven explicitly described UPFs as healthy foods.

### Free listing task

**Fig. 3:**
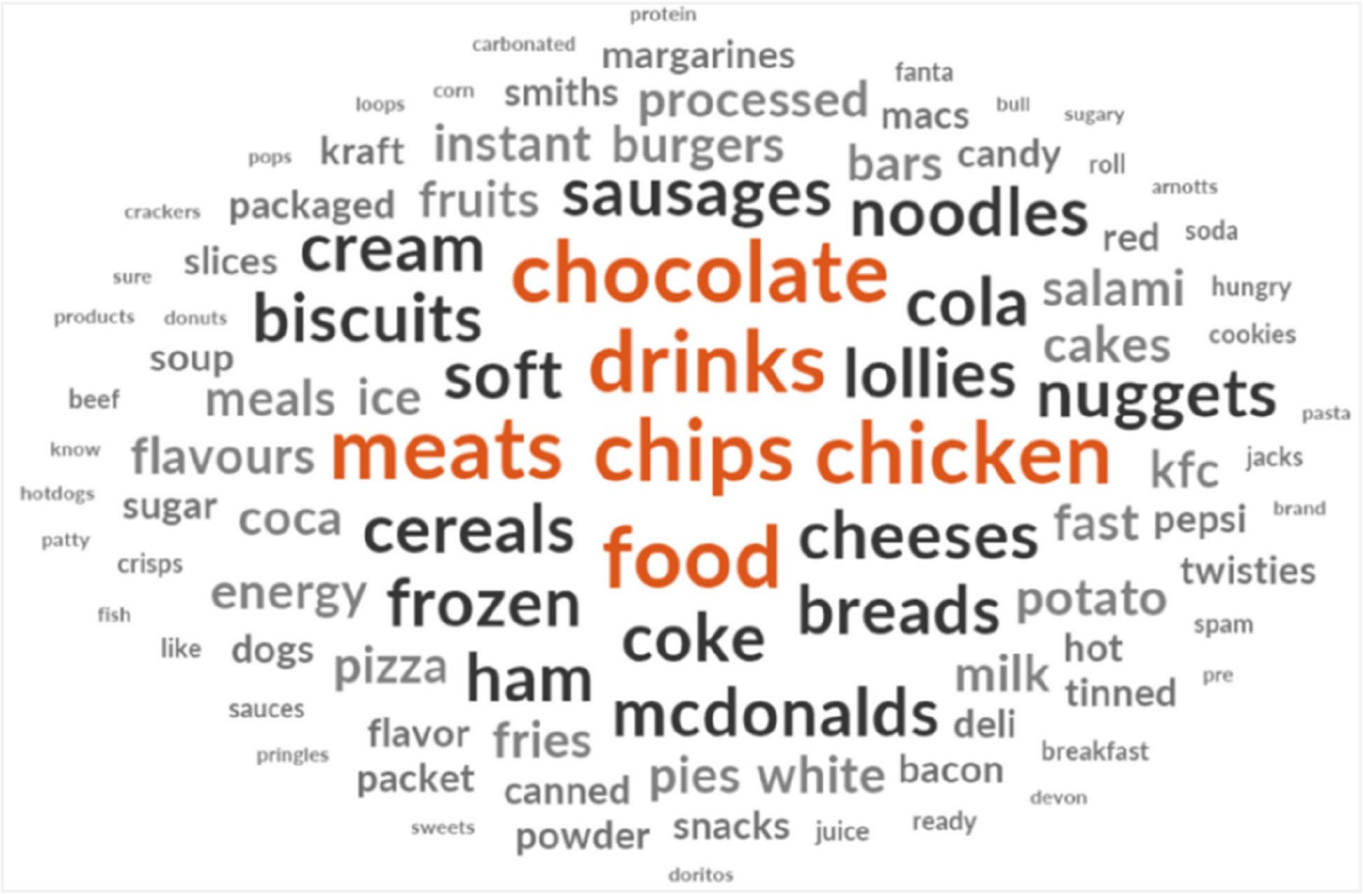
Word frequency for ultra-processed foods identified in the free listing task

Findings of the free listing task are presented in Fig. 3. Common examples of UPFs provided by participants include chocolate, soft drinks, chips, processed meats (e.g., nuggets, sausages, ham), biscuits and instant noodles. Many participants provided the name of brands or fast-food chains to illustrate their examples (e.g. McDonalds, Kraft).

### Differences between ultra-processed foods and other types of processed foods

**Fig. 4:**
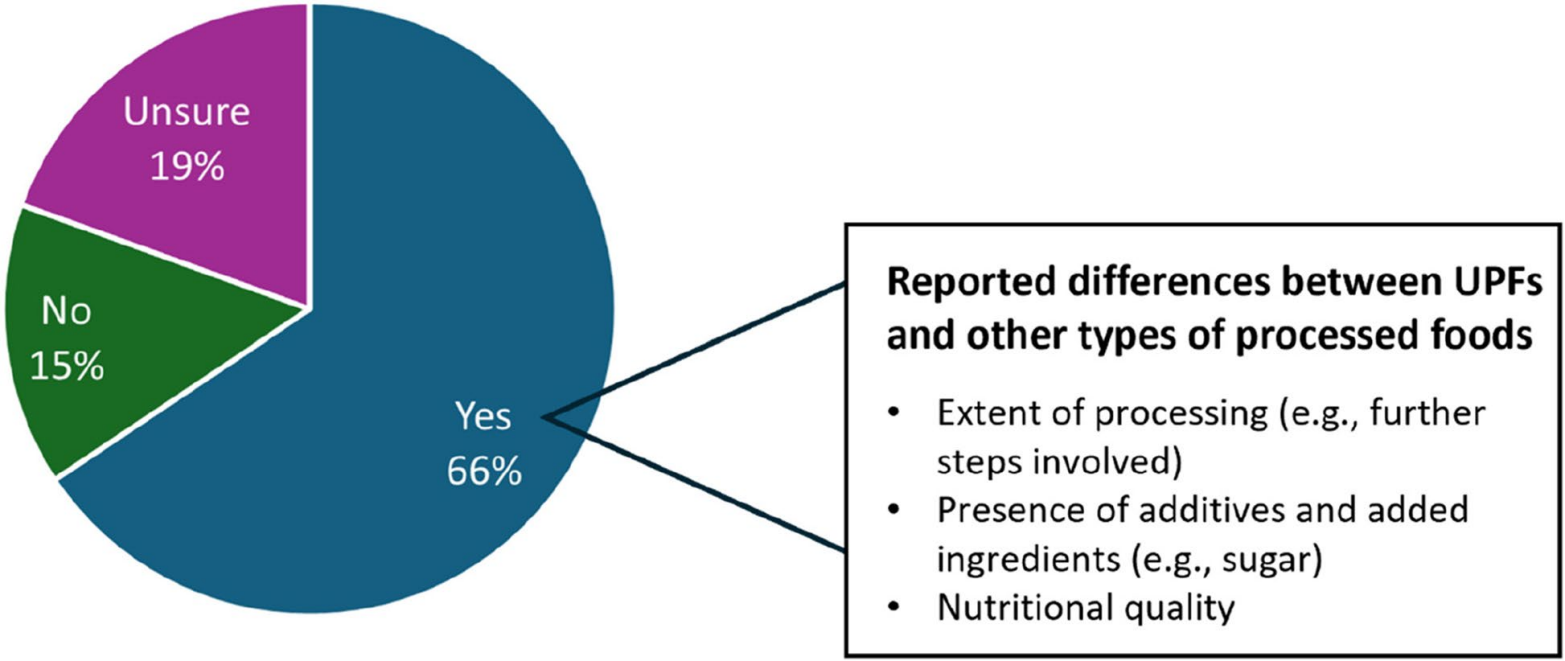
Proportion of participants reporting differences between UPF and other types of processed foods and reasoning for differences. Footnote: ‘Yes’ means that participants reported that UPFs and other types of processed foods differ; ‘No’ means that participants reported no differences; and ‘Unsure’ means that participants did not know if there were any differences

Sixty-six percent of the studied population reported differences between UPF and other types of processed foods, whereas 15% reported no differences, and 19% were unsure (Fig. 4). The main differences reported by participants were that UPFs undergo extensive industrial processes and contain many additives and added ingredients (e.g., sugar, salt and fat) making them different in composition and with a poorer nutritional quality compared to other processed foods. They also reported that processed foods that have undergone simpler processing techniques to enhance preservation (e.g., canning, freezing) and are closer to ‘natural’ state may still retain some nutritional value from their original ingredients.

### Attributes participants associated with UPFs

Table 4 presents the attributes participants associated with UPFs derived from the triad sorting technique. Some attributes were common for all four groups, while others were limited to only some groups. The attribute of artificial or unnatural ingredients was common across all four groups. Concerns about nutrient content (sugar and salt) were present for only the first two groups (milk drinks and snacks), whereas convenience was highlighted for only the latter two groups (meals). The frozen meals (group 3) were observed to have a longer shelf life, and the instant noodles and fast food (group 4) were viewed as cheaper. Finally, the UPFs in groups 2, 3 and 4 were viewed as unhealthy, whereas all foods in group 1 (milk drink products) were seen as healthy.

**Table 4:** Attributes participants associated with UPFs^*^

Survey images**	Group 1	Group 2	Group 3	Group 4
Attributes associated with UPFs *(products on the top in comparison to the one on the bottom)*	- Artificial flavour - Non-natural/artificial ingredients - Higher sugar content - All similar, e.g. healthy	- Presence of additives (e.g. colour and flavour) - Non-natural/artificial ingredients - Higher processing level - Sophisticated packaging - Higher salt content - Flavoured (vs plain) - Unhealthy (vs healthy)	- Frozen, packaged and extensively processed (vs homemade meal) - Artificial ingredients (vs fresh, natural ingredients) - More convenient - Higher shelf-life - Unhealthy (vs healthy)	- Artificial ingredients (vs fresh, natural ingredients) - More tasty - More convenient - Cheaper - Poorer nutritional quality - Unhealthy (vs healthy)

^*^Attributes identified through the question “In your view, how do the two products on the top differ from the one on the bottom?”; ** All items on the top are UPFs, and all items on the bottom are non-UPF

## Discussion

In this survey of Australian adults, most participants reported that they were familiar with the concept of UPFs. The dominant concepts and themes in participants’ responses reflected key characteristics of UPFs related to the extent of their processing (e.g., production involves several steps at the industrial level) and the presence of industrial ingredients such as flavours, colours and other additives. Examples of UPFs reported by participants included ‘fast food meals’, ‘confectionery’, ‘soft drinks’, ‘chips’, ‘processed meats’ and ‘frozen meals’. This was reflected in the correct identification of these items in the UPF classification task, but not others like multigrain packaged bread and flavoured yoghurt. Socio-demographic differences in UPF knowledge were also observed, with women, people with higher education, Non-Indigenous people and those with prior awareness about UPFs being more likely to correctly identify UPFs. Finally, attributes commonly associated with UPFs included presence of artificial ingredients, low nutritional quality, convenience, attractive packaging, palatability, and affordability. This study adds to the limited but growing area of research on public knowledge of UPFs [21–23].

While previous studies show varying levels of UPF awareness [21–24], 64% of participants reported awareness of the term in this study. This is surprisingly high considering that UPFs are not included in Australia’s official communications, unlike studies in other countries (e.g., Brazil, Uruguay), where UPF recommendations are included in their national dietary guidelines [15]. This may also reflect the higher proportion of individuals with a health background in the study sample compared to the general population [53]. Nevertheless, the overall increased awareness in Australia likely reflects the growing prominence of UPFs in public discourse, driven by expanding evidence and media coverage [12, 13, 54]. The number of publications and news media coverage on UPFs globally has increased by over 500% in the past 5 years [12, 55], and a similar trend has been observed in Australia [13]. While research on UPF content on social media remains limited, the large number of members (> 37,000) in the Reddit community dedicated to UPFs (r/ultraprocessedfood; top 4% based on size) suggests growing public engagement with the topic [56, 57].

Differences in UPF knowledge were observed across sociodemographic groups. Consistent with other literature in Australia, Canada, UK, Mexico and US [30], UPF knowledge was higher among women, older adults, highly educated and Non-Indigenous people. Behavioural differences and socioeconomic inequalities are a possible explanation for these discrepancies. Women, older adults, and those experiencing the least disadvantage appear to have higher nutrition literacy [26–30]. Women are more likely to seek out nutrition-related information [58], which can also explain our findings that women were more likely to describe UPFs in terms of their healthiness than men. Finally, socioeconomic inequalities and racism could have impacted opportunities for those with lower education or Indigenous Australians to develop and apply nutrition knowledge and related skills [59].

In this study, participants commonly described UPFs in terms of the ingredients they contain (e.g. “*ingredients not commonly used in home cooking*”) and/or their processing techniques (e.g. “*foods highly modified from its original form*”). These findings suggest a generally high understanding of UPFs, despite previous criticism that the concept might be too complex [60]. In Australia, the term ‘discretionary’ was adopted in the current Dietary Guidelines [61] to denote ‘unhealthy’ foods, following focus testing of various alternatives (e.g., ‘extra foods’, ‘sometimes foods’, ‘occasional foods’) [62]. It has been suggested that terms like ‘junk food’ might be more appropriate for public communication in Australia [63]. However, there is no evidence to date to support that those terms would be more comprehensible than UPFs. ‘Junk food’ is a widely used term in high-income countries [14, 63], yet it carries a connotation of individual choice, willpower and morality [64], which may downplay the broader systemic factors influencing dietary patterns. These terms, and others such as Energy-Dense, Nutrient-Poor (EDNP) foods, have a strong nutrient-focus and disregard level of processing on their basis, diverging from contemporary nutrition science focus on dietary patterns and food processing [65, 66].

As observed in studies elsewhere [21–23], most participants were able to correctly identify non-UPFs and also those UPFs that are typically defined as “unhealthy” (e.g., fast food meals, confectionery, soft drink, chips, processed meats) [14]. However, we found participants were less consistently able to identify UPFs in food categories commonly seen as healthy (e.g. dairy products and breads). This shows that a significant proportion of UPFs may not be easily identified as UPFs by consumers, including those that are highly consumed by the Australian population (e.g., mass-produced packaged bread, breakfast ‘cereals’) [5]. This is not a surprise, considering that most of these foods are marketed by food manufacturers as healthy options [16] and recommended in many national dietary guidelines [15]. Conflicting information about the healthiness of UPFs may lead to public confusion and reduced trust in nutrition guidance. For example, evidence shows that exposure to conflicting nutrition information can lead to confusion and contribute to nutrition backlash (i.e., negative beliefs about and attitudes toward nutrition recommendations and research), which has been associated with lower intake of fruit and vegetables [67–69]. It is therefore important that public health messaging regarding UPFs that are typically viewed as healthy is carefully planned and tested. This includes considering emerging evidence on whether certain UPFs may be acceptable in small amounts as part of an overall dietary pattern that is not predominantly based on UPFs.

Another key finding was that many participants described UPFs using terms like ‘preservatives’ and ‘canned’, which are not defining UPF features according to the Nova system [1]. Additionally, one-third were unsure or reported no differences between UPF and other types of processed foods. This likely reflects long-standing public communication around food processing. While the use of food processing terminology in dietary guidelines is increasing [70], most still favour implicit or euphemistic terms (e.g., canned, commercial) [15]. This distinction is critical, as nearly all foods undergo some level of processing. Some techniques bring important safety benefits (e.g., preservation, fermentation), while others used in the manufacture of UPFs are shown to be harmful (e.g. high-temperature and pressure techniques used in extrusion processes) [8, 71], making broad criticisms of ‘processed’ foods unhelpful [2]. Of significant concern, the UPF industry has increasingly co-opted scientific debates on food processing by deliberately conflating general processing with ultra-processing [20].

These findings highlight important directions for future research. Studies could explore how UPF knowledge influences dietary behaviours, dietary patterns, and public support for UPF policies. It would also be beneficial to examine the factors that shape this knowledge, including public engagement with government messaging, exposure to news and social media, and UPF industry influence (e.g., through marketing, funding research and campaigns). Additionally, research should focus on identifying effective strategies to improve UPF knowledge in the population, including ways to support the public in identifying credible and accurate UPF information, and other interventions and policy actions to address increased UPF consumption.

### Implications for nutrition communication and policies

As the evidence of the UPF impacts on health, environment, and society more broadly increases [8, 9, 72], so too does the need for policies and interventions aimed at reducing their harms [11, 73]. Improving food and nutrition literacy about UPFs is important to reduce their consumption [74], and data shows that 1 in 3 Australians already consider a food’s level of processing when making purchasing decisions [75]. Nevertheless, strong policies are needed to address the structural drivers of UPF consumption [4, 38, 39] and public support is crucial for the implementation of these policies [40, 41]. Therefore, the findings of this study can guide the development of communication strategies that increase awareness and literacy about UPFs, foster public support for related policies, and support the implementation of dietary recommendations.

The UPF concept provides an opportunity to move away from communication strategies centred on individual nutrients and foods, to dietary patterns and food systems [76] (see Table 5). In this study, participants described UPFs mostly in terms of their *extent* of processing (e.g., presence of certain ingredients), but not the purpose (e.g., food created for profitability) [1, 77]). Corporations producing these foods were mentioned only in a few instances, mainly referring to fast-food chains and a few major food brands. We also found that the negative impacts associated with UPFs were mostly centred on their nutritional and health aspects (e.g. “*unhealthy, harmful chemicals, no nutrients, bad for health*”), but not the other impacts such as environmental, social, cultural or economic. Further, participants also noted beneficial aspects of UPFs – that they are convenient and cheap. These are important equity considerations that require considered policy responses. While this likely reflects the focus of existing literature and media coverage on health studies—with less attention given to topics like the environment [13]—it also highlights opportunities to incorporate these broader themes into UPF communication strategies [76, 78].

**Table 5:** Opportunities for UPF communication strategies in Australia

• Use descriptors that are salient to the public (e.g., presence of many ingredients not commonly found at home; processing involves several steps in factories)
• Distinguish UPFs from other types of processed foods
• Provide examples of UPFs not commonly identified by the public (e.g., pasta sauce, flavoured yoghurt, mass-produced packaged bread)
• Prioritise ultra-processed dietary patterns (i.e., amount and types of foods consumed, displacement of unprocessed and minimally processed foods) in UPF messaging, with less emphasis on the presence of specific ingredients, nutrients or foods
• Some population groups may benefit from further tailored messaging

### Strengths and limitations

This study has several strengths. To the best of our knowledge, this is the first study to explore UPFs knowledge in a nationally representative sample of Australian adults, increasing its generalisability. The questionnaire was reviewed in consultation with a panel of consumers from a range of social, health, and cultural backgrounds, and was pilot tested for further refinement to ensure clarity and relevance of the survey questions. The survey included a range of different measures to capture UPF understanding and knowledge, and the use of open-ended questions allowed for a more in-depth exploration. We used Leximancer for analysis of the large qualitative data, and evidence shows that it helps minimise researcher bias because it uses a standard and consistent approach to identify and count concepts in the text [50, 51]. The large sample size increases statistical power of the study and allowed stratified analysis by population subgroups. We used FoodProK to measure UPF knowledge, a functional measure that has been used in multi-country studies previously, enabling comparability across different contexts.

Nevertheless, some limitations need to be considered. The study used quota-based sampling to approximate national population proportions of Australian adults; however, it may not be fully representative due to potential selection bias among individuals with internet access and willingness to participate. For example, in comparison to the general Australia’s population, this study had a higher proportion of health professionals (15% vs 5% [53]) and a lower proportion of people born overseas (23% vs 31.5% [79]). The large sample size may have enabled detection of statistically significant differences in small parameter estimates, which may not reflect meaningful differences across population subgroups. Brands were removed from the products to avoid copyright issues, which may have limited the ability of some participants to identify UPFs based on the familiarity with certain brands. Products for UPF selection tasks and triad sorting technique were selected based on data from the 2011–12 national nutrition survey data (most recent survey at the time of the study) and pilot study feedback, and they may not represent common products consumed by participants. It is also unclear whether participants’ ability to correctly identify UPFs and non-UPFs would have changed if a different set of products was used.

In conclusion, this national mixed-methods survey of Australian adults found that most participants were aware of the concept of UPFs and could identify key characteristics, such as industrial processing and the use of additives. Commonly recognised UPFs included fast food, soft drinks, confectionery, and processed meats. However, some foods like multigrain bread and flavoured yoghurt were often misclassified. Participants’ level of knowledge varied by socio-demographic factors, with women, more educated individuals, non-Indigenous participants, and those previously aware of UPFs more likely to correctly identify them. Characteristics that participants commonly associated with UPFs were low nutritional quality, convenience, attractive packaging, palatability, and affordability. Future research could focus on exploring how UPF knowledge influences dietary behaviours, dietary patterns, and public support for UPF policies; the factors that shape this knowledge (e.g., public engagement with government messaging, industry influence, and news and social media exposure); effective strategies to enhance UPF knowledge in the population, and interventions and policy actions to address increased UPF consumption.

## Supplementary Information

Supplementary Material 1.

## Data Availability

The datasets used and/or analysed during the current study are available from the corresponding author on reasonable request.
