# Supplementary material for: Public knowledge of ultra-processed foods: Insights from a national survey of Australian adults
Source: Nutr J. 2026 Jun 5;25:87. doi: 10.1186/s12937-026-01345-3 (PMC13459941; doi:10.1186/s12937-026-01345-3)
Supplement: Supplementary file 1 [file 12937_2026_1345_MOESM1_ESM.pdf]

Supplementary file 1. Description of the pilot study to develop the questionnaire to measure public knowledge of ultra-processed foods in Australia

The pilot study aimed to develop and test a questionnaire to measure public knowledge of ultra-processed foods in Australia.

Preliminary questionnaire development and consultation with consumers

The first version of the survey questionnaire was developed in Qualtrics by the research team drawing on previous literature[1,2]. The questionnaire was tested by a panel of five consumers to generate evidence of its face validity (i.e., whether the items were perceived as clear, understandable, and relevant to assessing knowledge of ultra-processed foods). The panel comprised adults living in Australia (2 young women, 1 middle aged woman, 1 middle aged man, 1 older woman, across both regional and major cities of Australia), recruited from the Deakin IPAN Consumer Network. During a 2-hour online workshop, held via Zoom in August 2023, consumers had the opportunity to trial the survey and provided feedback on survey length, appropriateness and clarity of instructions, questions, response options, and images, and identified repetitive or missing questions and items. Feedback was incorporated to the survey in Qualtrics, after discussion with the research team. Examples of how the survey was modified after feedback are presented below. A one-minute video explaining ultra-processed foods was also developed and included in the end of the survey (i.e., once participants had submitted their responses). This was suggested by consumers to provide participants an understanding of this concept without influencing their answers.

**Before consultation**	**After consultation**
“What do you understand by the term 'ultra-processed foods’?”	“What comes to your mind when you think of the term 'ultra-processed foods'? How would you describe this term?”
-	New question:<br>“Prior to today, had you heard about ultra-processed foods?”

Pilot test with a convenience sample

A cross-sectional online survey was conducted to pilot test the survey with a convenience sample of Australian adults, using the structured questionnaire developed in Qualtrics. Participants were eligible if they were aged 18-65y, living in Australia at the time of the survey and with access to a device with internet connection. Recruitment occurred over a three-week period using institutional (Deakin IPAN) social media (Facebook, LinkedIn, and Twitter) and website listing. Participation was anonymous and voluntary. Data collection occurred between February and March 2024, in English only.

A total of 52 participants completed the survey, with an average of 20 min to complete (range 6-58min). Among them, 62% were women, 57% aged 30-39 years (median 35 years, range 24-65years), 100% had completed Year 12 or an equivalent level of education, 70% spoke English at home, 75% lived in the State of Victoria, 100% did not identify as Aboriginal, Torres Strait Islander or both, and 65% did not have a professional background in health. Ninety-six percent reported awareness about UPFs prior to the pilot survey (Table S1). Responses were content analysed and key findings were summarised and informed survey refinement.

Table S1. Characteristics of the pilot study participants (n= 52).

**Demographics**	
**Gender (%)**	
Male	38
Female	62
**Age, median (min, max)**	35.3 (24.4, 65.7)
**Age, %**	
24-29 years	13.7
30-39 years	56.9
40-49 years	15.7
50-65 years	13.7
**Ability to communicate in English, %**	
Yes	100
**Language spoken at home, %**	
English	79
Other	21
**Aboriginal and Torres Strait Islander, %**	
Neither Aboriginal or Torres Strait Islander	100
**Education, %**	
Year 12 or equivalent	100
**States and territories, %**	
VIC	75
NSW	12
QLD	6
SA	6
TAS	2
WA	0
NT	0
**Professional background in health, %**	
Yes	35
No	65
**Prior awareness about UPFs, %**	
Yes	96
No	4

Final questionnaire development

After team discussion, the survey was modified considering the responses from the pilot study as follows:

Modifications to the survey after pilot study:

**Block 1: Pilot Demographic questions**	**Decision for national survey**
• What is your date of birth? (dd/mm/yyyy)<br>• How do you describe your gender?<br>• Can you communicate confidently in English? E.g., speak, read, and write English confidently<br>• Which language do you mainly speak at home? If more than one language, indicate the one that is spoken most often<br>• Are you of Aboriginal origin, Torres Strait Islander origin, or both?<br>• What is the highest year of primary or secondary school that you have completed?<br>• Suburb<br>• Postcode<br>• Do you have a professional background in health? (e.g. doctor, dietitian, nutritionist, pharmacist, etc.)	**Keep each question in its form, except:**<br>- Changed date of birth to "How old are you?"<br>- Added "What is your country of birth?"<br>- Removed 'suburb' as postcode is the only geographic-related variable being used.
**Block 2: Pilot UPF questions**	**Decision for national survey**
"What comes to your mind when you think of the term 'ultra-processed foods'? How would you describe this term?"	**Keep the question in its form.**<br><br>Justification: Participants answered as expected.
"Please list up to 10 different types of foods or drinks that you consider to be ultra-processed"	**Modifications:**<br>- Include wording to guide respondents to be more specific. New question now reads: "Please list up to 10 different types of foods or drinks that you consider to be ultra-processed. Please be specific as possible (e.g., product type, flavour and brand)."
	**New question added to the survey:**<br>- "In your view, is there a difference between ultra-processed foods and other types of processed foods? If so, how?"

	Justification: It was unclear in the pilot study if people could differentiate UPFs from other types of processed foods (e.g., many people reported issues with 'preservatives' or high content of salt, sugar and fat). This question would help to get more direct responses.
"In this section, we would like you to select any ultra-processed foods and drinks among those items presented below. You can click on the image to view it in full size."<br><br>Items included:<br>1. Chicken nuggets<br>2. Instant noodles<br>3. Ready meal lasagne<br>4. Fast food burger<br>5. Flavoured water<br>6. Chocolate block<br>7. Cereal bar<br>8. Flavoured yoghurt<br>9. Bolognese pasta sauce<br>10. Tortilla strips corn<br>11. Multigrain packaged bread<br>12. Cheese block<br>13. Canned corn<br>14. Canned fish<br>15. Full cream milk<br>16. 100% Fruit juice<br>17. Pasta<br>18. Plain yoghurt<br>19. Chicken breast<br>20. Oats	**Suggested modifications:**<br><br>- Replace 'chicken breast' and 'oats' with potentially more controversial items since >95% did not select as UPF. Add instead: (1) non-UPF Bread and (2) Muesli.
"Can you briefly tell us how did you make the decision whether a food or drink below was ultra-processed? For instance, did you consider elements from the packaging or rely on what you already know about these foods? Your insights will help us understand your perspective better."	**Keep the question in its form.**<br>*Justification*: It helps identify rationale for identifying ultra-processed foods (including misconceptions) at the product level.

Block 3: Triadic sorting technique	Decision for national survey
"In your view, how do the two products on the top differ from the one on the bottom? Please include both the top and bottom products in your response."	**Keep the question in its form.**<br>Changes only in the pictures of foods provided to make sure the 3 images represented examples from equivalent groups (e.g., all dairy-based).
**Block 4: FoodProK**	**Decision for national survey**
"Overall, how healthy is this food product? Please move the bar under the image on a scale from 0 to 10, where 0 = Not at all healthy, and 10 = Extremely healthy."	**Keep the question in its form.**
**Block 5: Final questions**	**Decision for national survey**
"Is there anything else you would like to tell us about ultra-processed foods?"	**Keep the question in its form.**
"Prior to today, had you heard about ultra-processed foods?"	**Keep the question in its form.**
**Overall**: brands were removed for the main study questionnaire due to copyright issues.	

The final survey was developed in Qualtrics, containing 9 questions about characteristics of the population (e.g., demographics), 2 open-ended questions about understanding of ultra-processed foods, one free-listing task, one ultra-processed food identification task (with 20 pictures of products, 10 being ultra-processed foods), one triadic sorting technique with four groups of foods and beverages, the assessment of healthiness perception using the Food Processing Knowledge (FoodProK) Score method[2], and final questions about awareness of the ultra-processed food term prior to the study, and other comments.

References for supplementary file 1:

1. Ares G, Vidal L, Allegue G, Gimenez A, Bandeira E, Moratorio X, et al. Consumers' conceptualization of ultra-processed foods. Appetite. 2016;105:611-7.

2. Bhawra J, Kirkpatrick SI, Hall MG, Vanderlee L, Hammond D. Initial Development and Evaluation of the Food Processing Knowledge (FoodProK) Score: A Functional Test of Nutrition Knowledge Based on Level of Processing. Journal of the Academy of Nutrition and Dietetics. 2021;121(8):1542-50.

Intro

What do consumers think about ultra-processed foods?

Welcome and thank you for your interest in this study.

Our aim is to recruit adults living in Australia (aged 20-64 years) to understand what consumers think about ultra-processed foods.

This survey will take approximately 10-20 minutes.

Eligibility

Before beginning the survey, it's important that you have a full understanding of what's involved in participation. We have attached a Plain Language Statement, which provides details about this study.

It is important to read the Plain Language Statement, as it clearly describes the research, why we are doing it, what you can do if you would like to withdraw, how your information is protected and how you can contact us if you need further information.

Please click here to open the Plain Language Statement. If you would like to, you can save a copy for your records.

Consent statement
I have read and understand the Plain Language Statement.

I understand my participation is voluntary.

I freely agree to participate in this project according to the conditions in the Plain Language Statement.

I have been given access to a copy of the Plain Language Statement to keep.

I understand all data I provide will remain confidential and only be used for the purposes described in the Plain Language Statement.

I have been informed that the confidentiality of the information I provide will be safeguarded subject to any legal requirements.

The researchers have agreed not to reveal my identity and personal details, including where information about this project is published, or presented in any public form.

- ◯ I agree to the above statements and give my full consent to participate in the Study

Eligibility

To allow us to determine if you are eligible, we would like to ask you a couple of a quick questions.

Are you aged between 20 and 64 years?

- ◯ Yes
- ◯ No

Eligibility

To allow us to determine if you are eligible, we would like to ask you a couple of a quick questions.

Are you currently living in Australia?

- ◯ Yes

○ No

Demographic questions

Thank you, you are eligible for this survey.

How old are you?

[ ]

Thank you, you are eligible for this survey.

How old are you?

[ ]

How do you describe your gender?

○ Man or Male
○ Woman or Female
○ Non-Binary
○ I use a different term (please specify): [ ]
○ Prefer not to answer

Can you communicate confidently in English? E.g., speak, read, and write English confidently

○ Yes
○ No

Which language do you mainly speak at home? If more than one language, indicate the one that is spoken most often

- English
- Mandarin
- Arabic
- Cantonese
- Vietnamese
- Italian
- Greek
- Hindi
- Spanish
- Punjabi
- Other (please specify): ______

What is your country of birth?

______

Are you of Aboriginal origin, Torres Strait Islander origin, or both?

- Aboriginal
- Torres Strait Islander
- Both Aboriginal and Torres Strait Islander
- Neither Aboriginal or Torres Strait Islander

What is the highest year of primary or secondary school that you have completed?

- Year 12 or equivalent
- Year 11 or equivalent
- Year 10 or equivalent
- Year 9 or equivalent
- Year 8 or equivalent
- Year 7 or below
- Never attended school

Postcode

Do you have a professional background in health? (e.g. doctor, dietitian, nutritionist, pharmacist, etc.)

- Yes
- No

UPF questions

We are interested to hear what consumers like you think about different types of foods and how that impacts your choices. These next questions are all about your thoughts on specific foods.

What comes to your mind when you think of the term 'ultra-processed foods'? How would you describe this term?

Please list up to 10 different foods or drinks that you consider to be ultra-processed. Please be specific as possible (e.g., product type, flavour and brand).

In your view, is there a difference between ultra-processed foods and other types of processed foods? If so, how?

In this section, we would like you to select any ultra-processed foods and drinks among those items presented below. You can click on the image to view it in full size.

☐ » Fast food burger

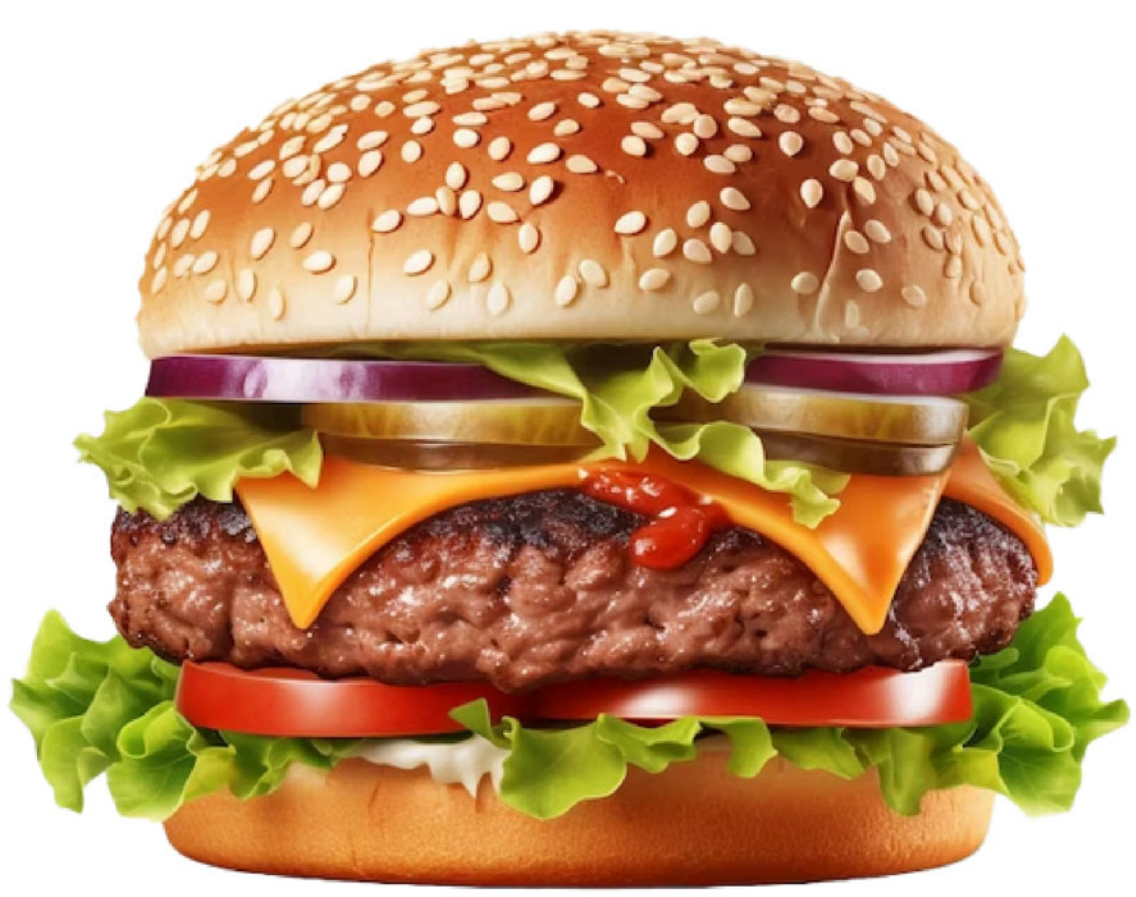

☐ Chocolate block

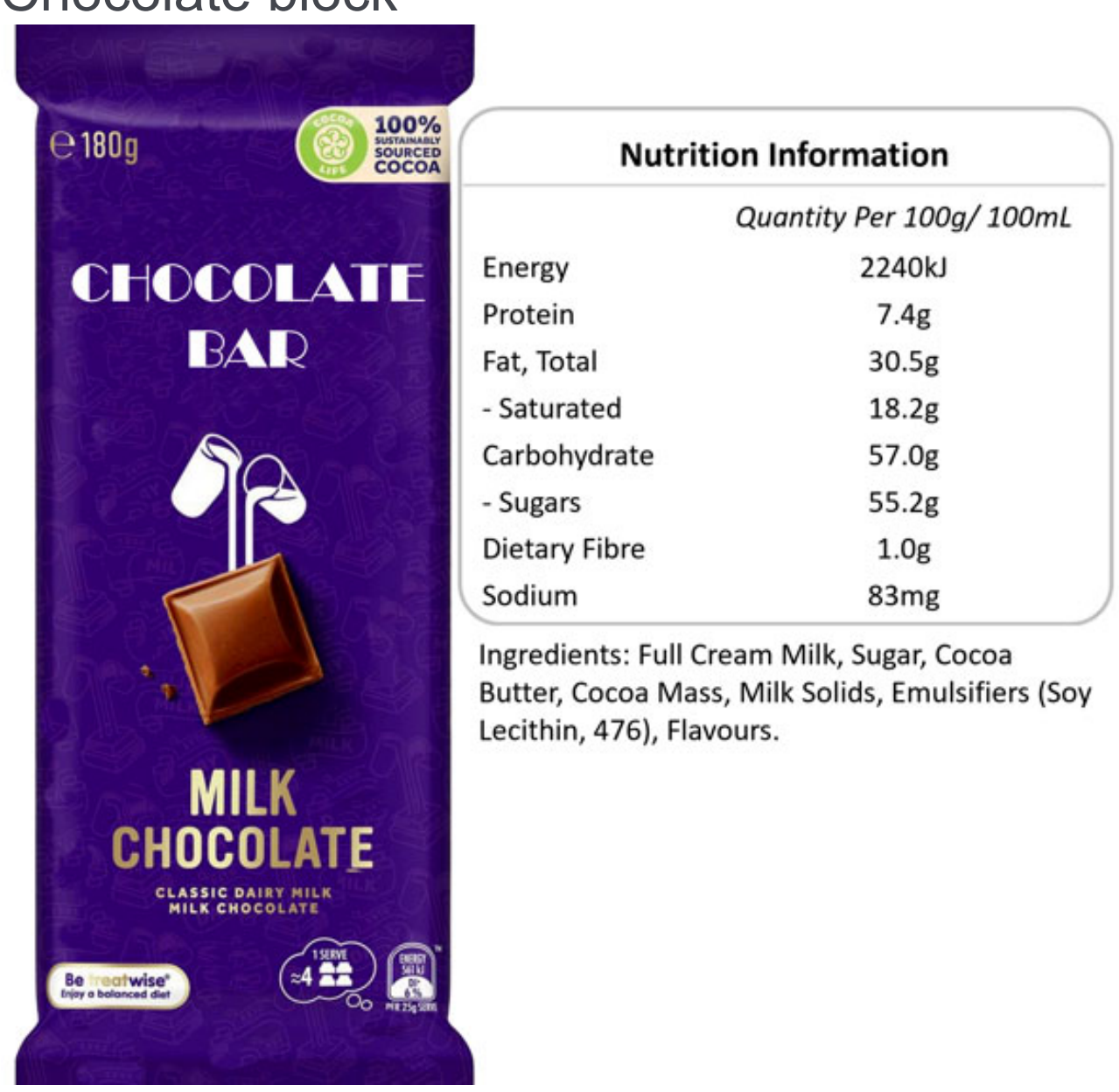

☐ Instant noodles

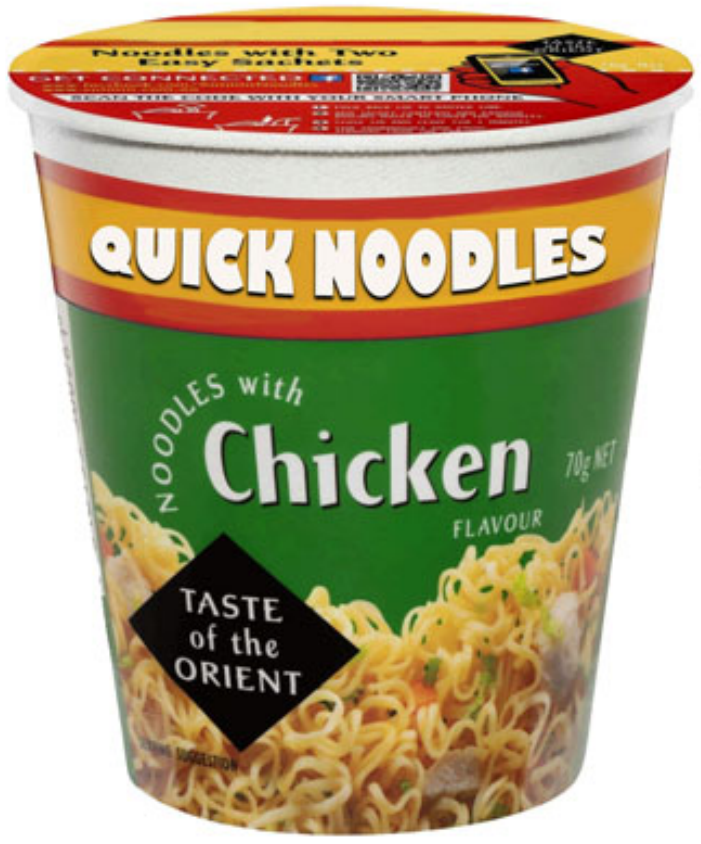

Nutrition Information	Quantity Per 100g/ 100mL
Energy	378kJ
Protein	1.7g
Fat, Total	3.6g
- Saturated	2.1g
Carbohydrate	11.3g
- Sugars	0.4g
Dietary Fibre	8.4g
Sodium	420mg

Ingredients: Noodles: Wheat Flour, Vegetable Oil (Antioxidant (320)), Salt.
Flavour Sachet: Salt, Flavour Enhancers (621, 635), Maltodextrin, Flavour, Anticaking Agent (551), Chicken Powder, Onion Powder, Herbs and Spice, Vegetable Oil (Antioxidant (307b)), Colours (150a, 100).
Vegetable Sachet: Dried Carrot, Textured Vegetable Protein, Dried Onion.

☐ » Chocolate block

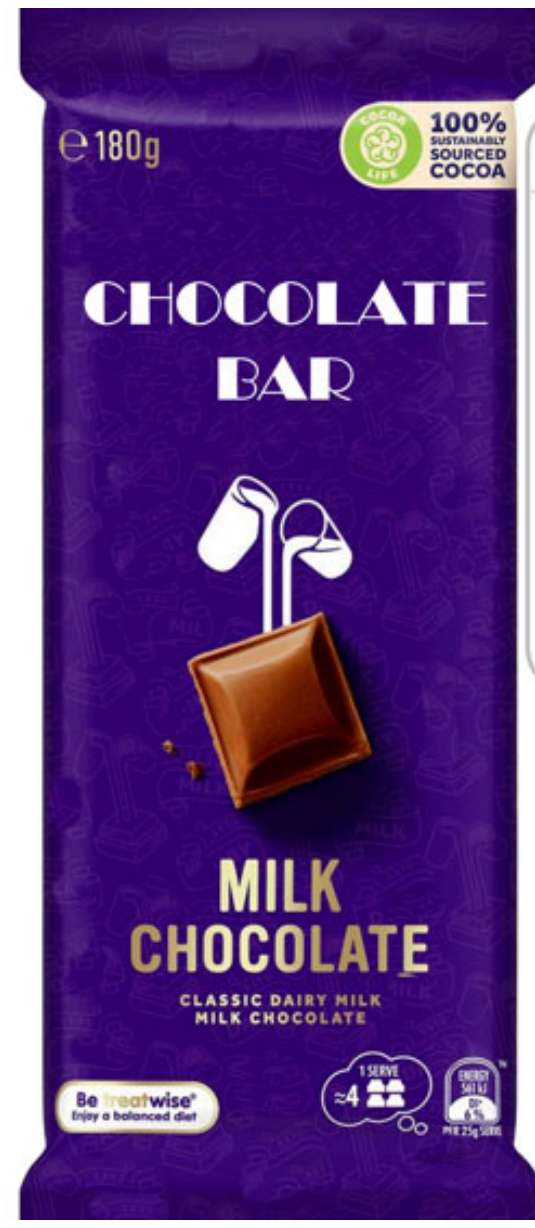

Nutrition Information	Quantity Per 100g/ 100mL
Energy	2240kJ
Protein	7.4g
Fat, Total	30.5g
- Saturated	18.2g
Carbohydrate	57.0g
- Sugars	55.2g
Dietary Fibre	1.0g
Sodium	83mg

Ingredients: Full Cream Milk, Sugar, Cocoa Butter, Cocoa Mass, Milk Solids, Emulsifiers (Soy Lecithin, 476), Flavours.

☐ Cereal bar

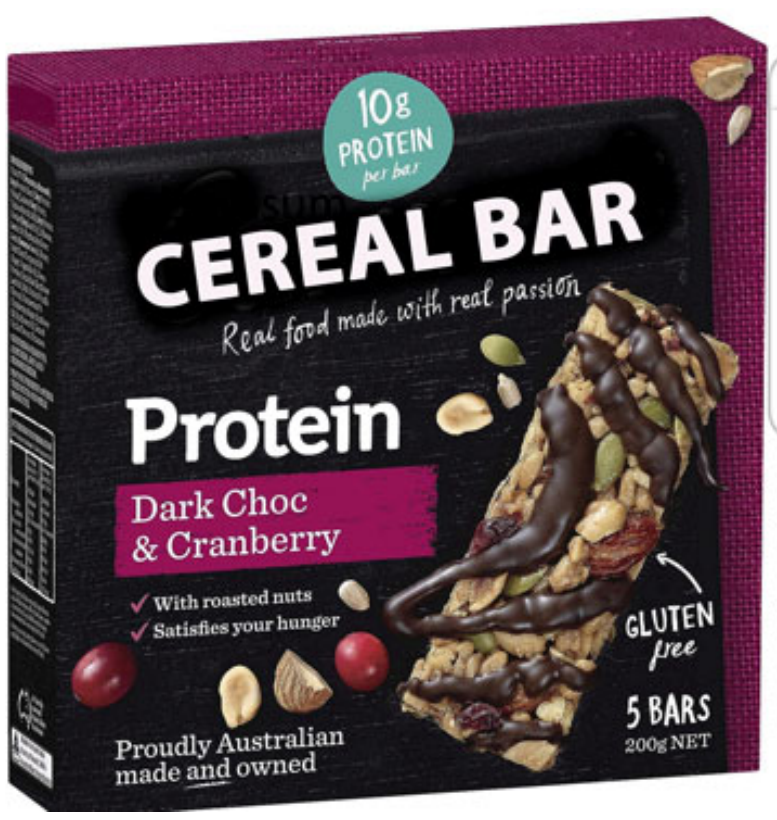

Nutrition Information	Quantity Per 100g/ 100mL
Energy	2060kJ
Protein	26.2g
Fat, Total	26.7g
- Saturated	5.6g
Carbohydrate	33.5g
- Sugars	23.0g
Dietary Fibre	6.6g
Sodium	130mg

Ingredients: Nuts 26% (Peanuts, Almonds), Plant Protein Blend (Soy Protein Crisps Isolated Soy Protein, Tapioca Starch, Salt, Soy Protein Isolate), Glucose, Seeds 11% (Sunflower, Pepitas, Sesame), Honey, Dark Choc Compound 9% (Sugar, Vegetable Oil, Cocoa Powder, Emulsifier Soy Lecithin, Sunflower Lecithin, Salt, Natural Flavour, Milk), Cranberries 7% (Sugar, Cranberries, Sunflower Oil), Sunflower Oil, Natural Flavours, Cinnamon.

☐ Multi-grain packaged bread

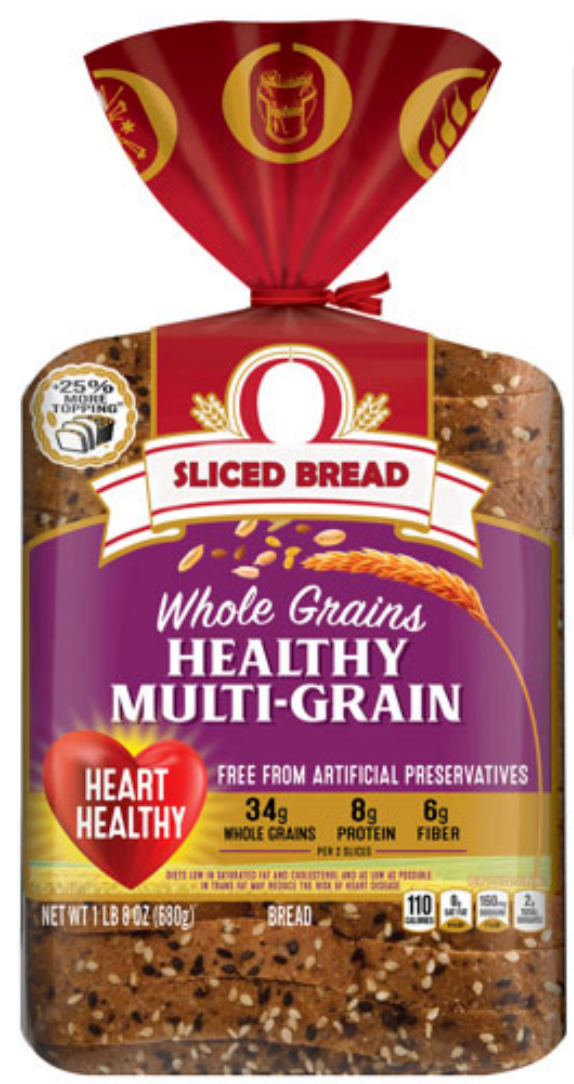

Nutrition Information	Quantity Per 100g/ 100mL
Energy	1166kJ
Protein	11.6g
Fat, Total	5.8g
- Saturated	0.0g
Carbohydrate	46.2g
- Sugars	9.3g
Dietary Fibre	6.7g
Sodium	419mg

Ingredients: Whole Wheat Flour, Water, Sugar, Wheat Gluten, Brown And Golden Flaxseed, Yeast, Honey, Bulgur Wheat, Vegetable Oil (Soybean), Cornmeal, Oats, Brown Rice, Steel Cut Oats, Sunflower Seeds, Sea Salt, Cellulose Fibre, Molasses, Cultured Wheat Flour, Grain Vinegar, Natural Flavors, Soy Lecithin, Citric Acid, Oat Fibre.

☐ » Bakery white bread

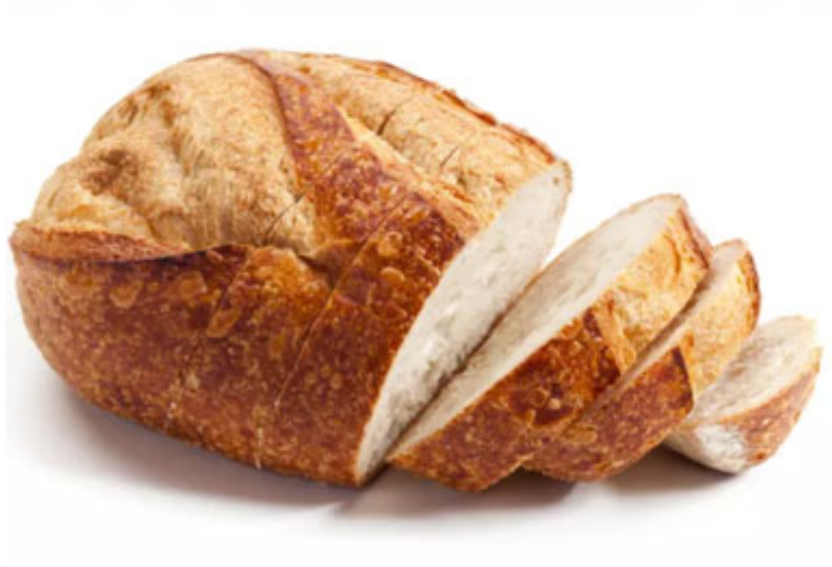

Nutrition Information	Quantity Per 100g/ 100mL
Energy	1040kJ
Protein	9.3g
Fat, Total	1.4g
- Saturated	0.3g
Carbohydrate	45.6g
- Sugars	2.7g
Dietary Fibre	2.6g
Sodium	440mg

Ingredients: Wheat Flour, Water, Sourdough Culture (Water, Wheat Flour, Whole meal Wheat Flour), Whole meal Wheat Flour, Iodised Salt, Yeast, Vitamins (Thiamin, Folic Acid).

☐ » Canned corn

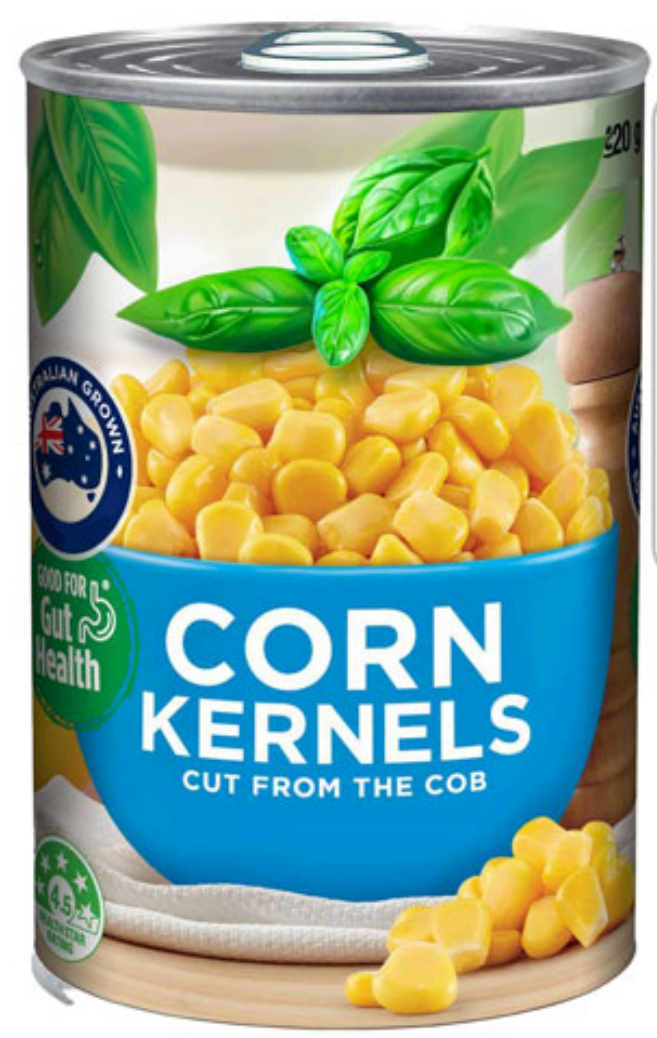

Nutrition Information	Quantity Per 100g/ 100mL
Energy	275kJ
Protein	2.5g
Fat, Total	1.9g
- Saturated	0.4g
Carbohydrate	8.2g
- Sugars	3.2g
Dietary Fibre	2.6g
Sodium	241mg

Ingredients: Sweet corn (58%), water, salt.

☐ Fast food burger

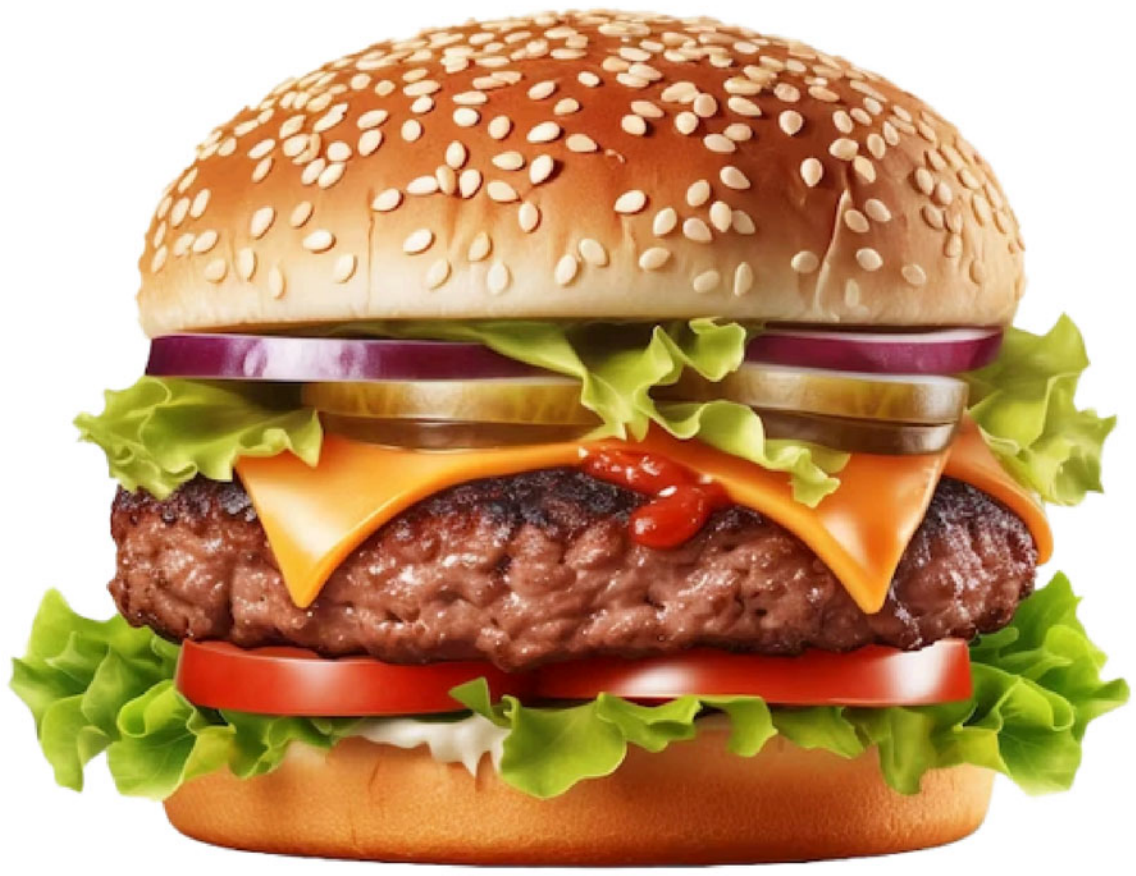

☐ Ready meal lasagne

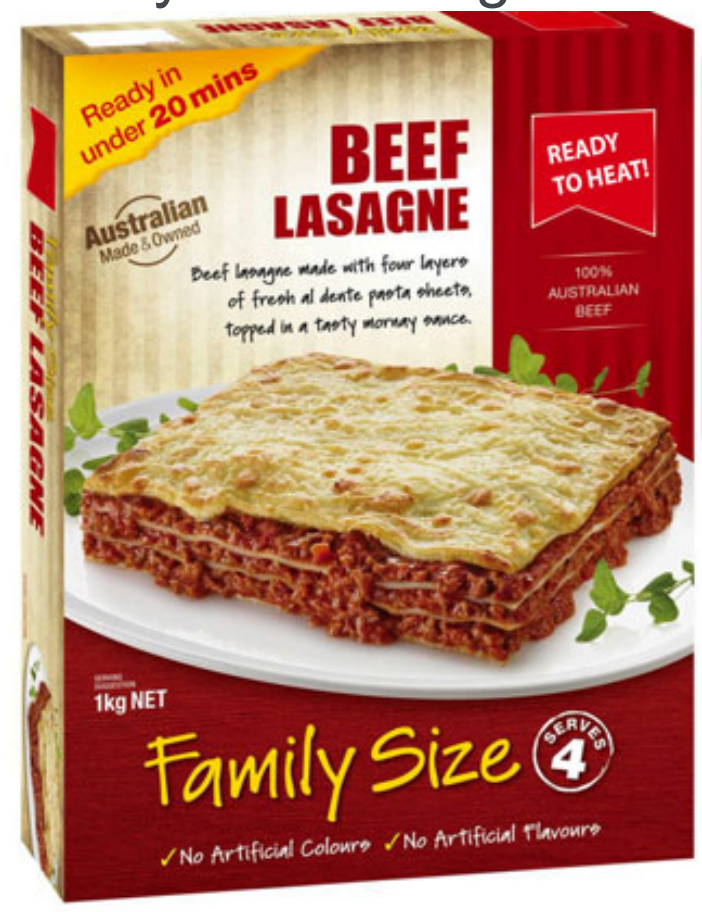

Nutrition Information

	Quantity Per 100g/ 100mL
Energy	607kJ
Protein	5.3g
Fat, Total	6.2g
- Saturated	3.1g
Carbohydrate	16.1g
- Sugars	2.2g
Dietary Fibre	2.1g
Sodium	277mg

Ingredients: Water, Cooked Pasta (27%) [Wheat Semolina, Water], Milk, Beef (11%), Tomato Paste (Tomatoes, Food Acid (330), Cheese (Milk), Onion, Vegetable Spread (Soy), Thickener (1422), Wheat Flour, Carrot, Sugar, Salt, Spices, Herbs, Yeast Extract, Food Acid (330), Flavour Enhancers (635).

☐ » 100% Fruit juice

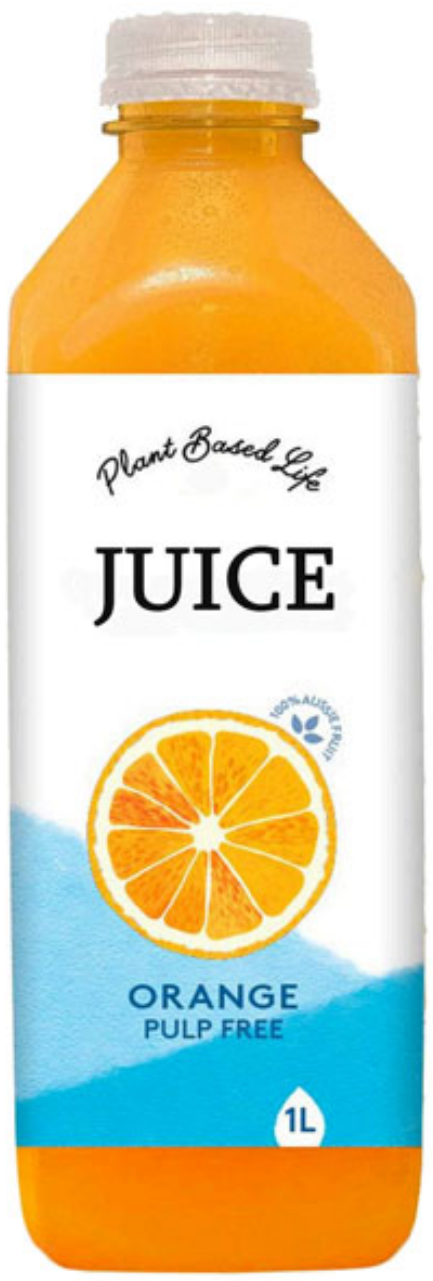

Nutrition Information

	Quantity Per 100g/ 100mL
Energy	493kJ
Protein	1.2g
Fat, Total	0.1g
- Saturated	0.1g
Carbohydrate	27.2g
- Sugars	21.3g
Dietary Fibre	0.1g
Sodium	1.0mg

Ingredients: 100% Orange Juice.

☐ Tortilla strips corn

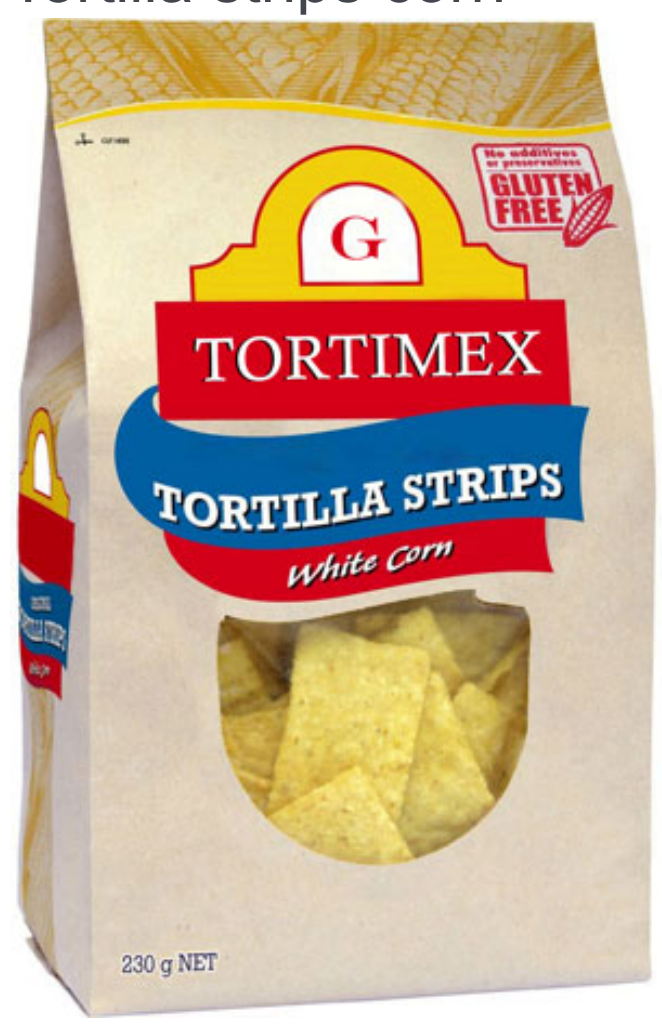

Nutrition Information

	Quantity Per 100g/ 100mL
Energy	2140kJ
Protein	6.3g
Fat, Total	26.5g
- Saturated	2.3g
Carbohydrate	59.2g
- Sugars	1g
Dietary Fibre	2.1g
Sodium	330mg

Ingredients: White Corn Flour (76%), Vegetable Oil , Water, Salt.

☐ » Chicken nuggets

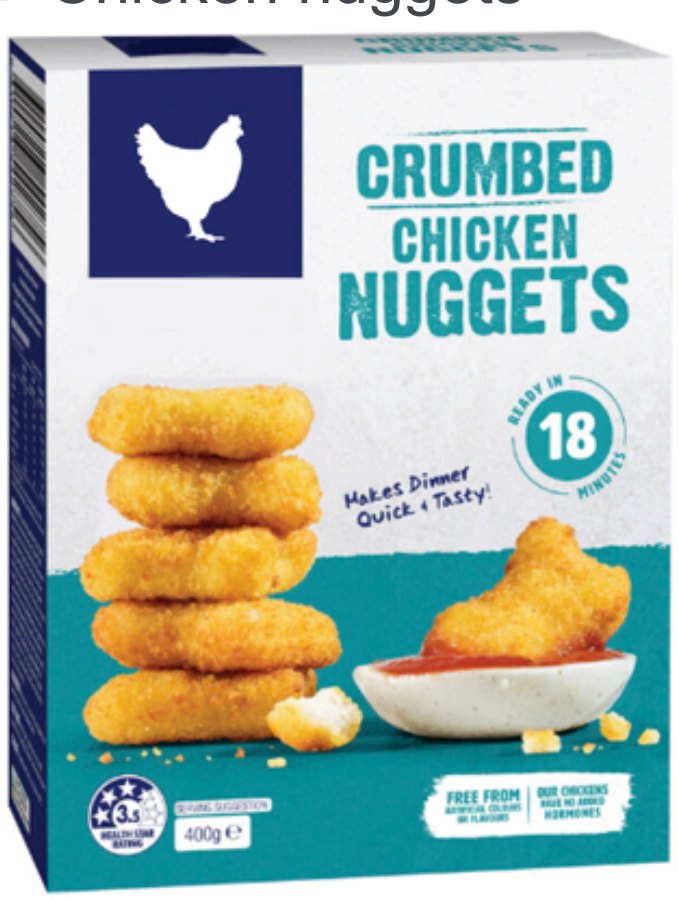

Nutrition Information

	Quantity Per 100g/ 100mL
Energy	962kJ
Protein	11.1g
Fat, Total	12.5g
- Saturated	6.2g
Carbohydrate	18.2g
- Sugars	1.9g
Dietary Fibre	1.0g
Sodium	500mg

Ingredients: Chicken Breast (41%), Coating (37%) Potato Flakes (Potato, Emulsifier (471), Mineral Salt (450) Preservative (223) (Sulphites), Food Acid (330)), Wheat Flour, Rice Flour, Salt, Emulsifier (481), Dextrose, Spice Extracts (160c, 100), Water, Wheat Gluten, Wheat Starch, Vegetable Oil, Thickener (1404), Mineral Salts (450, 500) Vegetable Gums (412, 415), Water, Vegetable Oil, Tapioca Starch (Sulphites), Soy Protein, Mineral Salts (451, 450), Dehydrated Vegetables (Onion, Garlic), Yeast Extract.

☐ » Canned fish

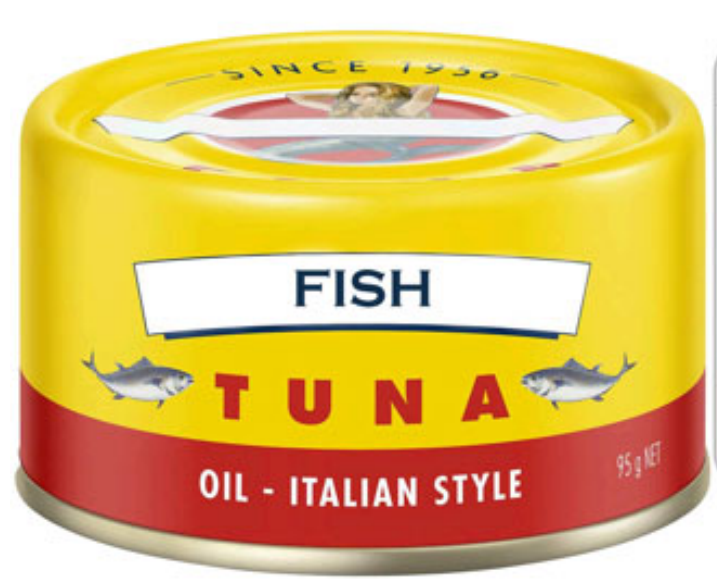

Nutrition Information

	Quantity Per 100g/ 100mL
Energy	809kJ
Protein	26.0g
Fat, Total	9.9g
- Saturated	1.2g
Carbohydrate	1.0g
- Sugars	1.0g
Dietary Fibre	-
Sodium	519mg

Ingredients: Pole & Line Caught Yellowfin Tuna (Fish)(73.5%), Oil (Sunflower Oil And Extra Virgin Olive Oil) (25%), Sea Salt.

☐ » Flavoured yoghurt

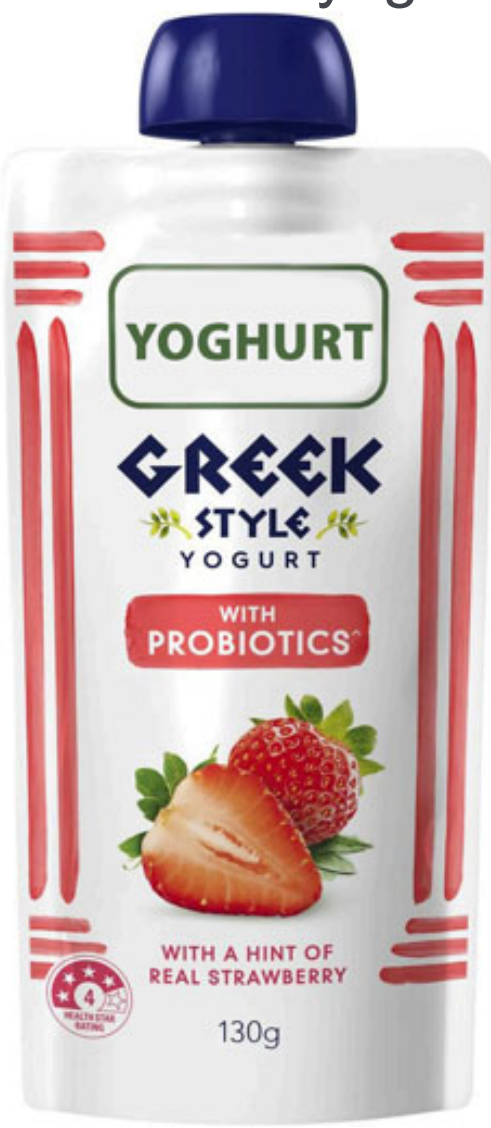

Nutrition Information

	Quantity Per 100g/ 100mL
Energy	376kJ
Protein	5.0g
Fat, Total	2.9g
- Saturated	1.9g
Carbohydrate	10.7g
- Sugars	10.2g
Dietary Fibre	0.0g
Sodium	50mg

Ingredients: Milk, Milk Solids, Strawberry (5%), (Puree, Reconstituted Juice), Water, Sugar, Cream (Milk), Thickener (Modified Starch), Natural Flavour, Natural Colour (Carrot Concentrate), Milk Minerals, Probiotic Cultures (Milk): B. lactis (BB-12), Yoghurt Cultures (Milk): S. Thermophilus, L. Bulgaricus.

☐ » Tortilla strips corn

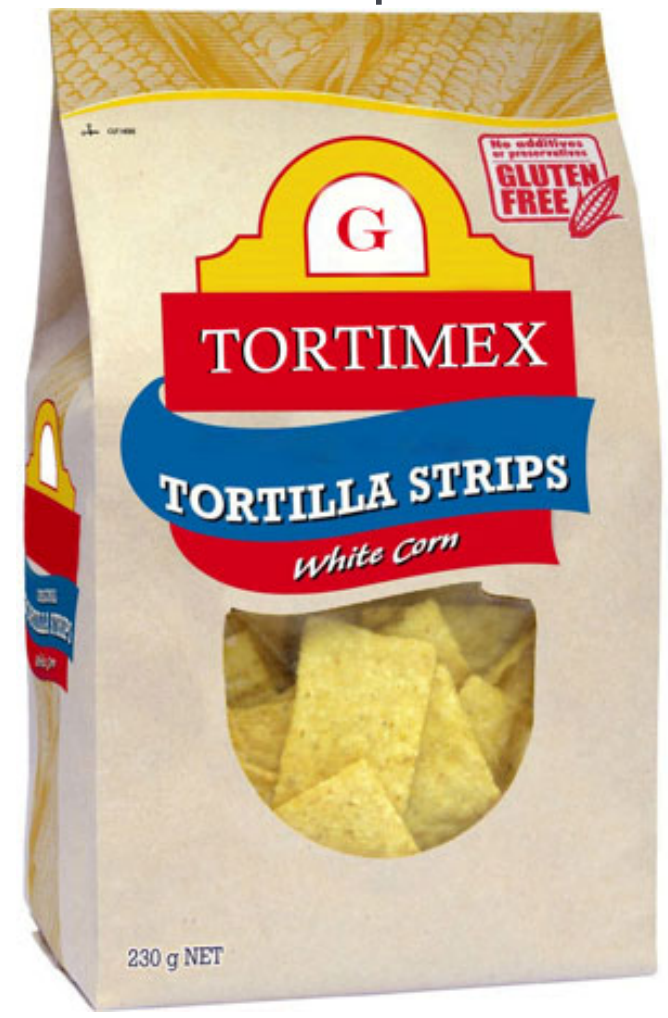

Nutrition Information

	Quantity Per 100g/ 100mL
Energy	2140kJ
Protein	6.3g
Fat, Total	26.5g
- Saturated	2.3g
Carbohydrate	59.2g
- Sugars	1g
Dietary Fibre	2.1g
Sodium	330mg

Ingredients: White Corn Flour (76%), Vegetable Oil , Water, Salt.

☐ Canned fish

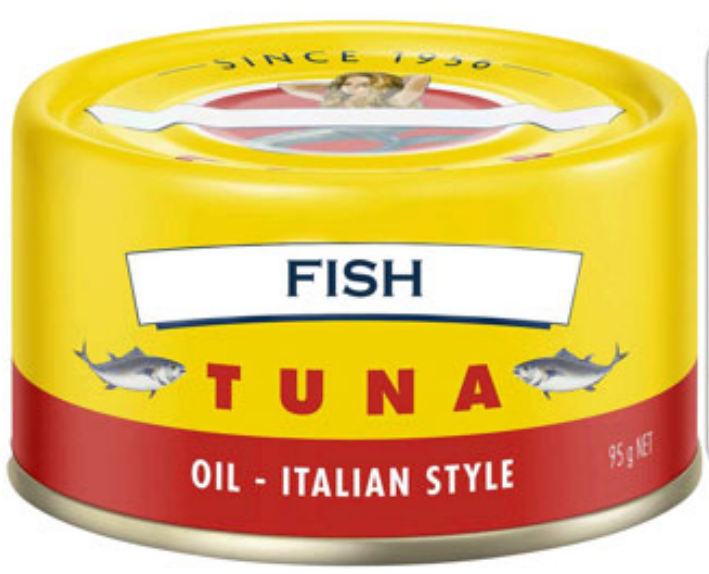

Nutrition Information

	Quantity Per 100g/ 100mL
Energy	809kJ
Protein	26.0g
Fat, Total	9.9g
- Saturated	1.2g
Carbohydrate	1.0g
- Sugars	1.0g
Dietary Fibre	-
Sodium	519mg

Ingredients: Pole & Line Caught Yellowfin Tuna (Fish)(73.5%), Oil (Sunflower Oil And Extra Virgin Olive Oil) (25%), Sea Salt.

☐ » Flavoured water

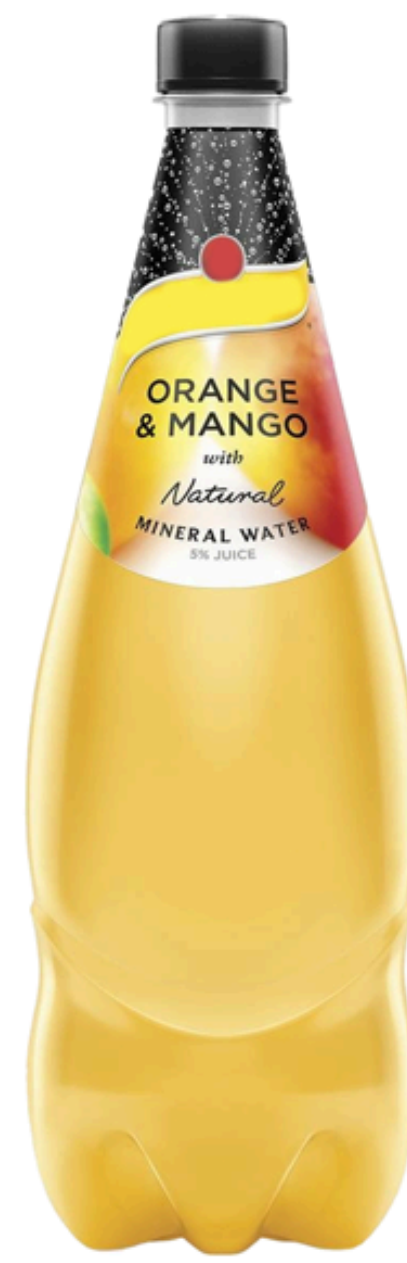

Nutrition Information

	Quantity Per 100g/ 100mL
Energy	65kJ
Protein	0.0g
Fat, Total	0.0g
- Saturated	0.0g
Carbohydrate	3.5g
- Sugars	3.4g
Dietary Fibre	-
Sodium	6mg

Ingredients: Carbonated Water (Mineral Water 80%, Purified Water 11%), Reconstituted Juices (Orange 4%, Mango 1%) , Sugar, Natural Flavours, Food Acids (Citric Acid , Ascorbic Acid), Preservatives (211, 202, 223 (SULPHITES)), Natural Colours (Carminic Acid, Lutein) , Antioxidant (385).

☐ Canned corn

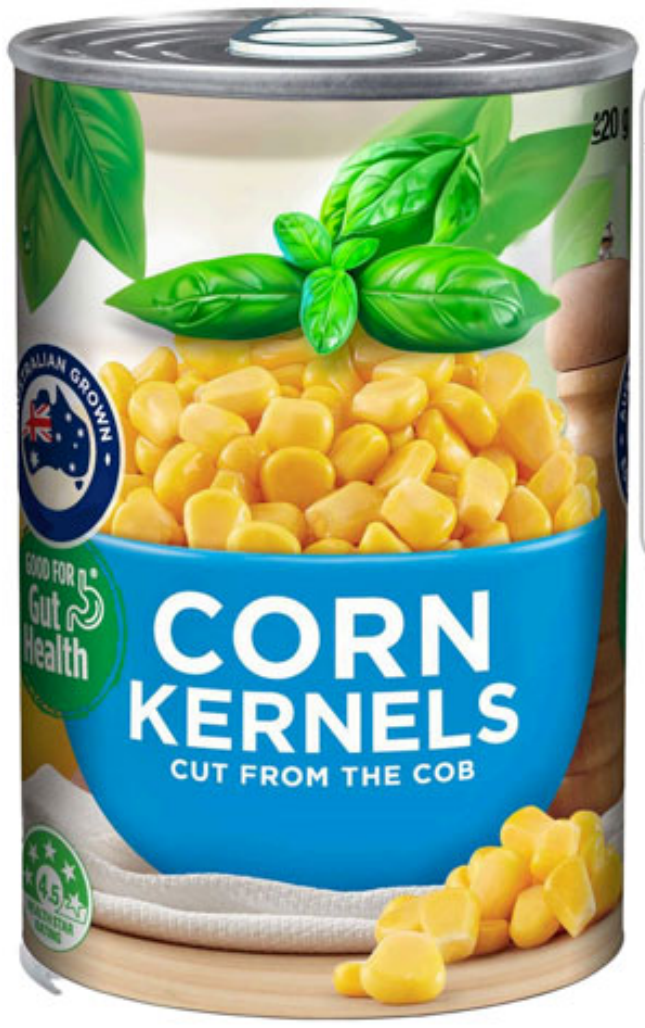

Nutrition Information	Quantity Per 100g/ 100mL
Energy	275kJ
Protein	2.5g
Fat, Total	1.9g
- Saturated	0.4g
Carbohydrate	8.2g
- Sugars	3.2g
Dietary Fibre	2.6g
Sodium	241mg

Ingredients: Sweet corn (58%), water, salt.

☐ Flavoured water

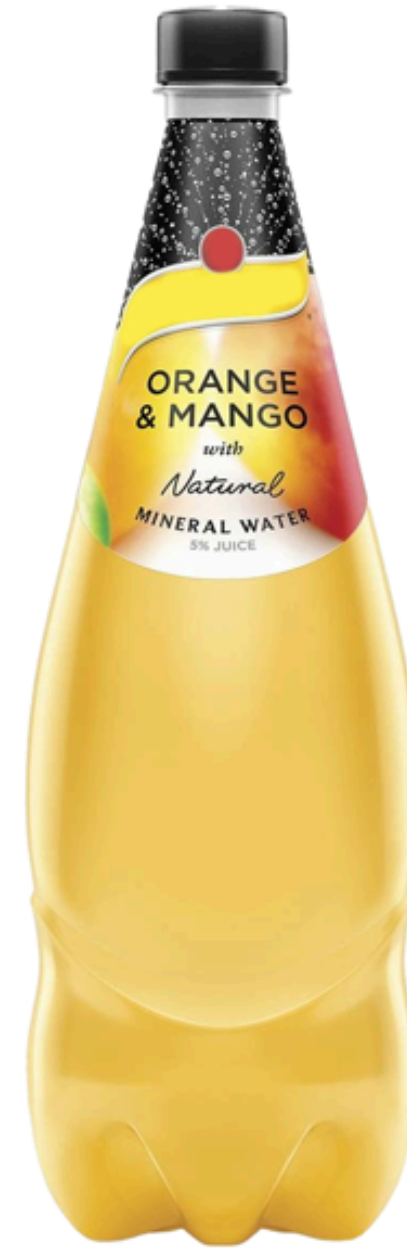

Nutrition Information	Quantity Per 100g/ 100mL
Energy	65kJ
Protein	0.0g
Fat, Total	0.0g
- Saturated	0.0g
Carbohydrate	3.5g
- Sugars	3.4g
Dietary Fibre	-
Sodium	6mg

Ingredients: Carbonated Water (Mineral Water 80%, Purified Water 11%), Reconstituted Juices (Orange 4%, Mango 1%) , Sugar, Natural Flavours, Food Acids (Citric Acid , Ascorbic Acid), Preservatives (211, 202, 223 (SULPHITES)), Natural Colours (Carminic Acid, Lutein) , Antioxidant (385).

☐ Muesli

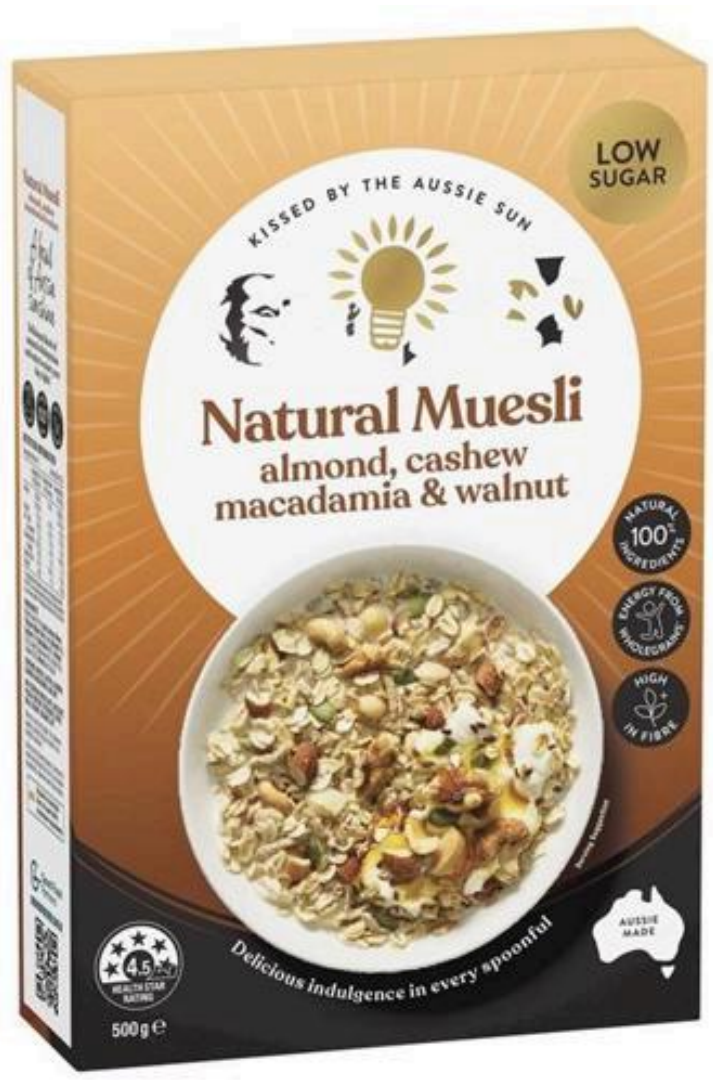

Nutrition Information	Quantity Per 100g/100mL
Energy	1700kJ
Protein	13.0g
Fat, Total	16.8g
- Saturated	2.3g
Carbohydrate	49.0g
- Sugars	1.7g
Dietary Fibre	9.6g
Sodium	8.0mg

Ingredients: Whole Grains (75%) Oats, Barley, Wheat, Triticale (Wheat, Rye), Nuts Almond (5%), Cashew (5%), Macadamia (2%) Walnut (2%), Seeds Sunflower (4%), Pepita (2%), Linseed (2%), Wheat Bran Straws.

☐ Pasta

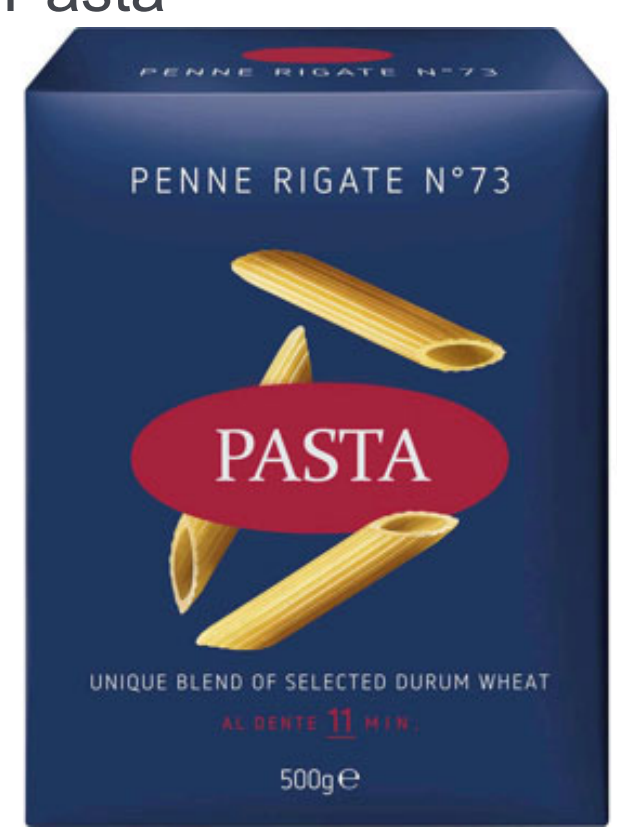

Nutrition Information	Quantity Per 100g/ 100mL
Energy	1520kJ
Protein	12.5g
Fat, Total	2.0g
- Saturated	0.5g
Carbohydrate	71.2g
- Sugars	3.5g
Dietary Fibre	3.0g
Sodium	5mg

Ingredients: Durum wheat semolina, Water.

☐ » Cheese block

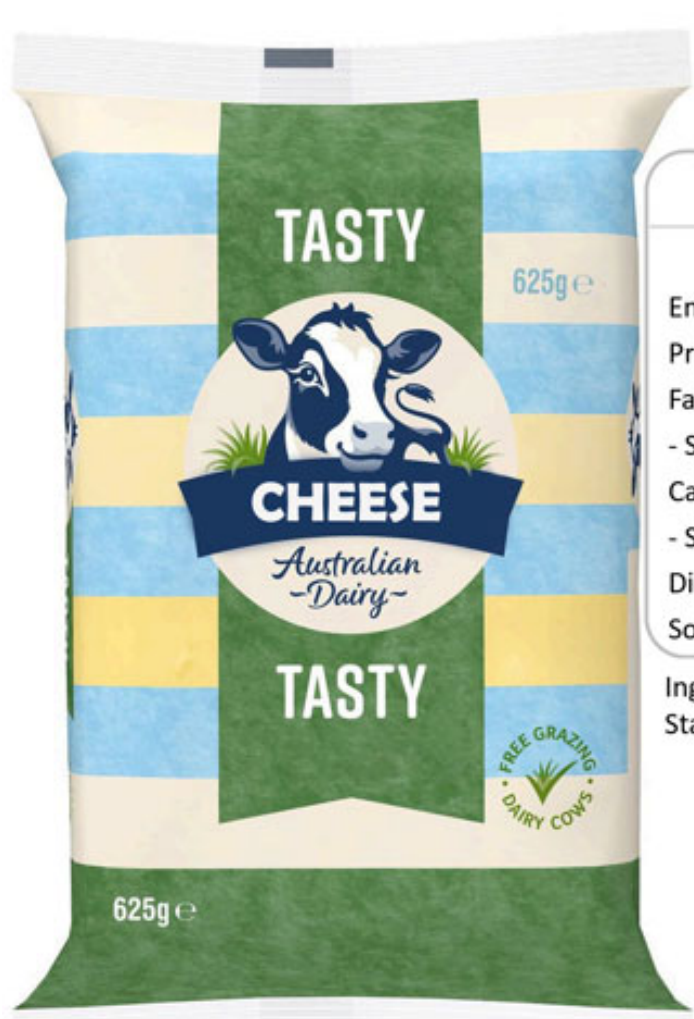

Nutrition Information	Quantity Per 100g/ 100mL
Energy	1730kJ
Protein	24.3g
Fat, Total	35.0g
- Saturated	23.9g
Carbohydrate	<1g
- Sugars	<1g
Dietary Fibre	-
Sodium	633mg

Ingredients: Cheese (Pasteurised Milk, Salt, Starter Cultures, Enzyme (Non-Animal Rennet)).

☐ Plain yoghurt

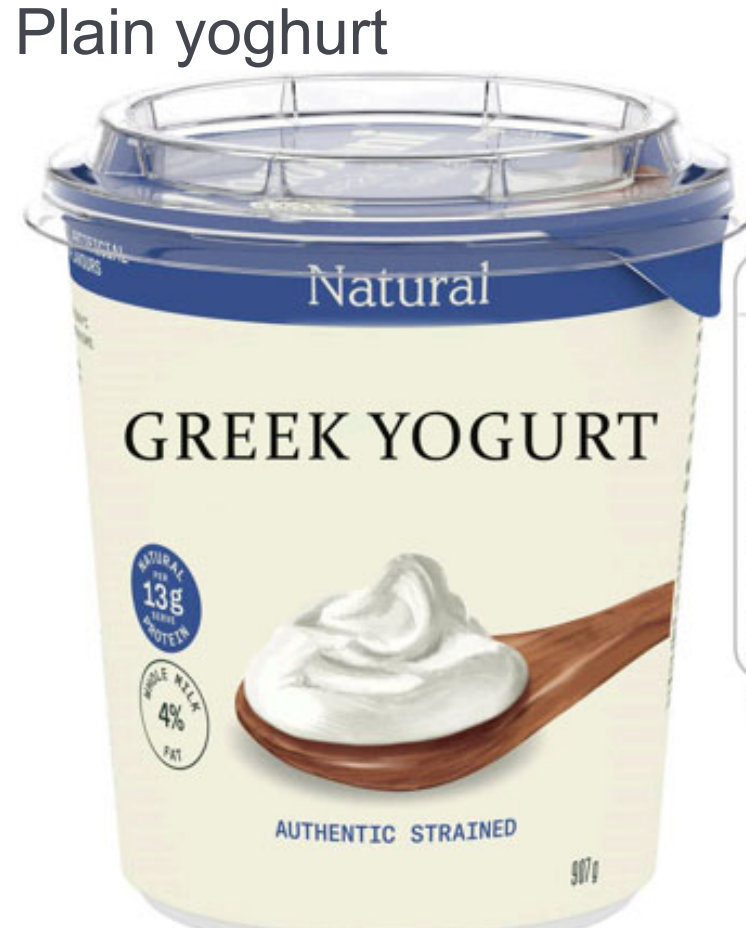

Nutrition Information	Quantity Per 100g/ 100mL
Energy	362kJ
Protein	8.7g
Fat, Total	4.0g
- Saturated	2.6g
Carbohydrate	3.9g
- Sugars	2.7g
Dietary Fibre	-
Sodium	33mg

Ingredients: Milk, Cream, Live Yogurt Cultures.

☐ » Multi-grain packaged bread

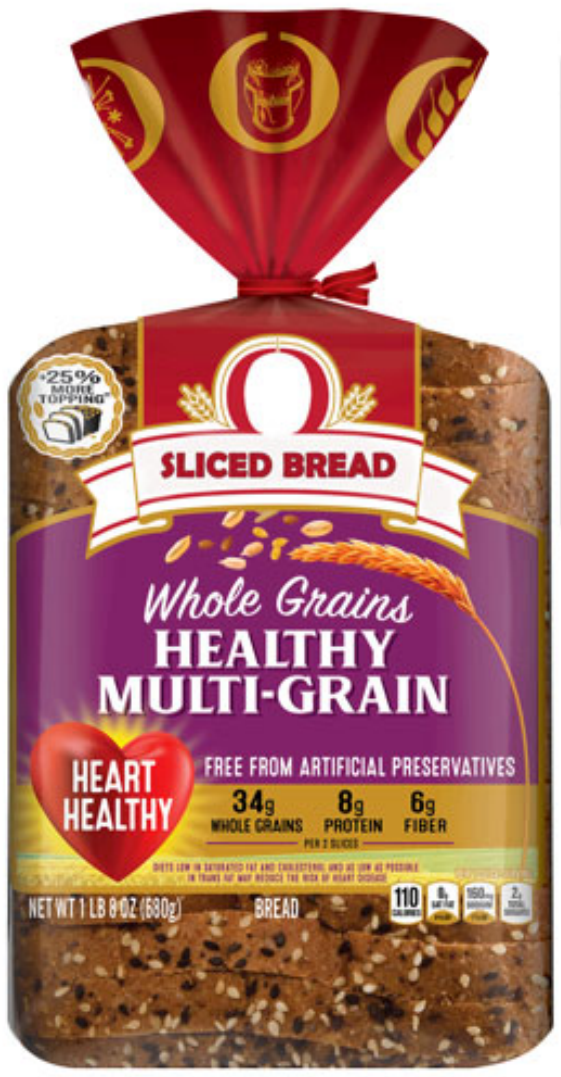

Nutrition Information	Quantity Per 100g/ 100mL
Energy	1166kJ
Protein	11.6g
Fat, Total	5.8g
- Saturated	0.0g
Carbohydrate	46.2g
- Sugars	9.3g
Dietary Fibre	6.7g
Sodium	419mg

Ingredients: Whole Wheat Flour, Water, Sugar, Wheat Gluten, Brown And Golden Flaxseed, Yeast, Honey, Bulgur Wheat, Vegetable Oil (Soybean), Cornmeal, Oats, Brown Rice, Steel Cut Oats, Sunflower Seeds, Sea Salt, Cellulose Fibre, Molasses, Cultured Wheat Flour, Grain Vinegar, Natural Flavors, Soy Lecithin, Citric Acid, Oat Fibre.

☐ Cheese block

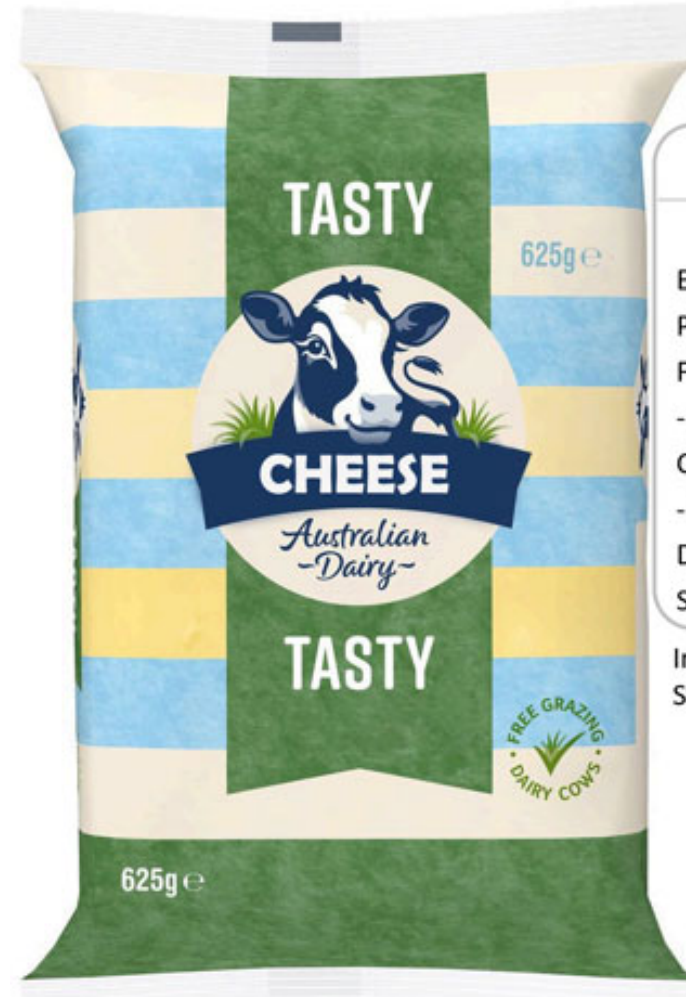

Nutrition Information	Quantity Per 100g/ 100mL
Energy	1730kJ
Protein	24.3g
Fat, Total	35.0g
- Saturated	23.9g
Carbohydrate	<1g
- Sugars	<1g
Dietary Fibre	-
Sodium	633mg

Ingredients: Cheese (Pasteurised Milk, Salt, Starter Cultures, Enzyme (Non-Animal Rennet)).

☐ » Muesli

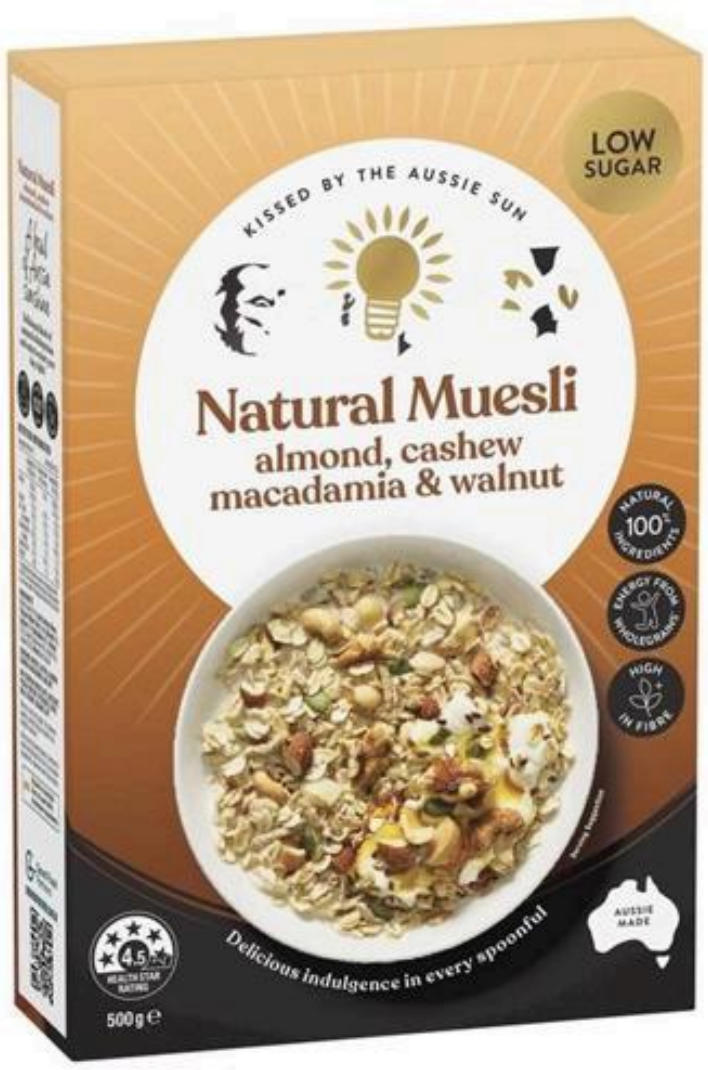

Nutrition Information	Quantity Per 100g/100mL
Energy	1700kJ
Protein	13.0g
Fat, Total	16.8g
- Saturated	2.3g
Carbohydrate	49.0g
- Sugars	1.7g
Dietary Fibre	9.6g
Sodium	8.0mg

Ingredients: Whole Grains (75%) Oats, Barley, Wheat, Triticale (Wheat, Rye), Nuts Almond (5%), Cashew (5%), Macadamia (2%) Walnut (2%), Seeds Sunflower (4%), Pepita (2%), Linseed (2%), Wheat Bran Straws.

☐ » Full cream milk

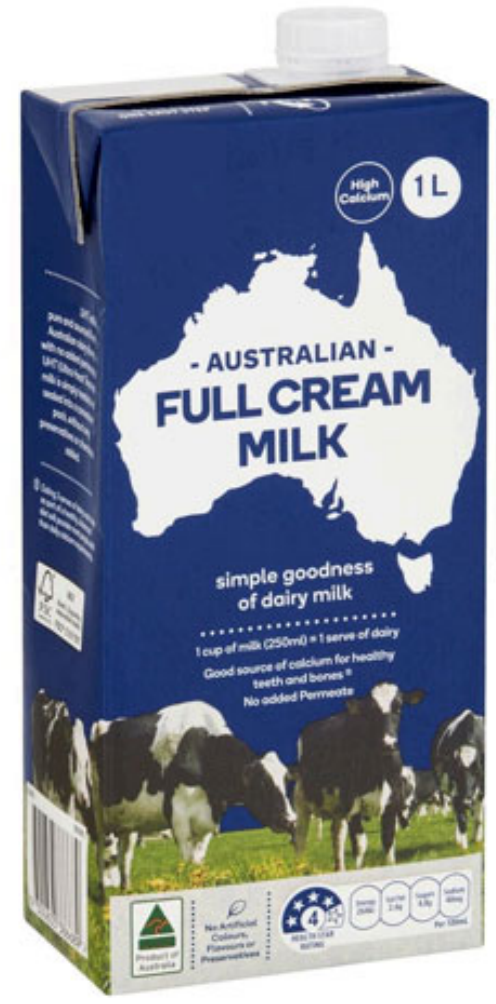

Nutrition Information	Quantity Per 100g/ 100mL
Energy	264kJ
Protein	3.3g
Fat, Total	3.2g
- Saturated	2.5g
Carbohydrate	4.8g
- Sugars	4.8g
Dietary Fibre	-
Sodium	40mg

Ingredients: Full Cream Milk.

☐ Bakery white bread

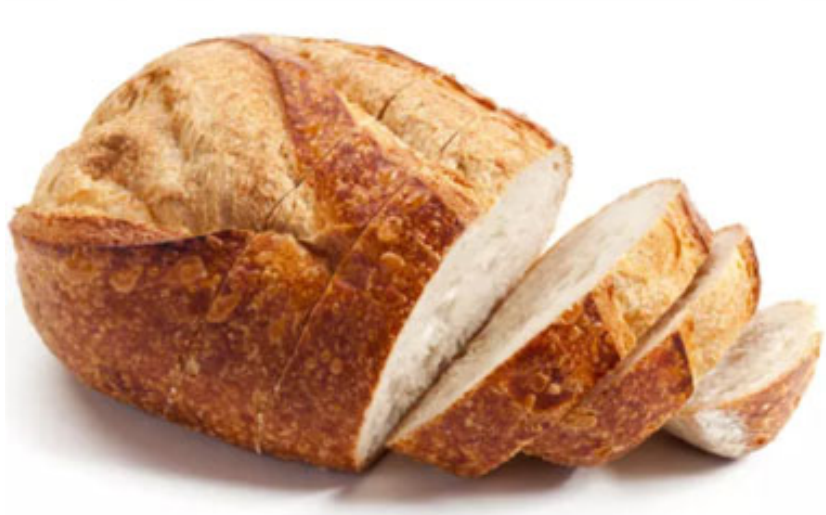

Nutrition Information	Quantity Per 100g/ 100mL
Energy	1040kJ
Protein	9.3g
Fat, Total	1.4g
- Saturated	0.3g
Carbohydrate	45.6g
- Sugars	2.7g
Dietary Fibre	2.6g
Sodium	440mg

Ingredients: Wheat Flour, Water, Sourdough Culture (Water, Wheat Flour, Whole meal Wheat Flour), Whole meal Wheat Flour, Iodised Salt, Yeast, Vitamins (Thiamin, Folic Acid).

☐ » Cereal bar

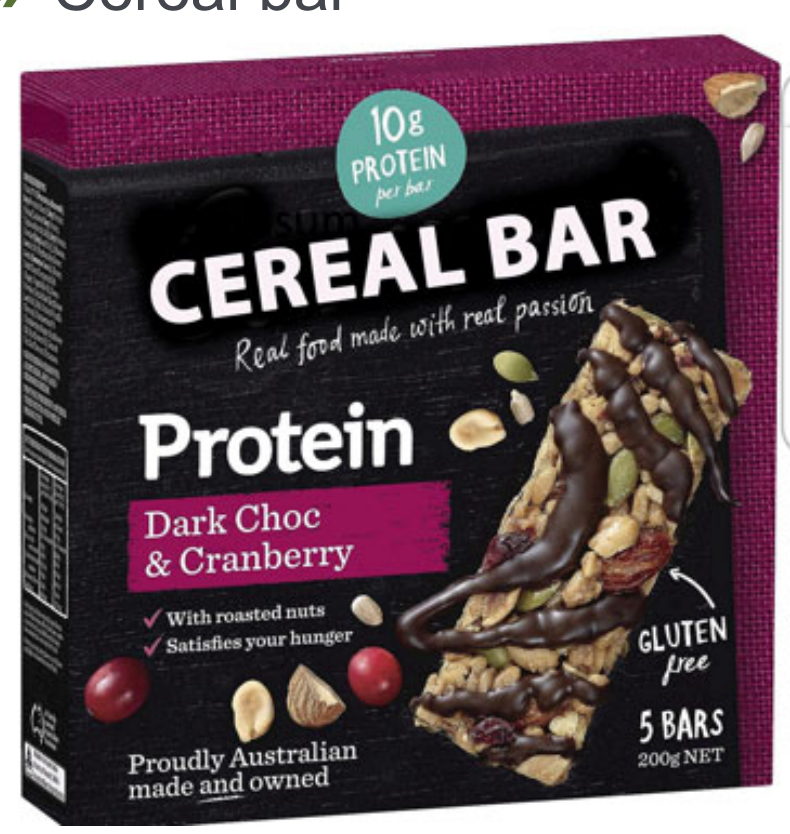

Nutrition Information	Quantity Per 100g/ 100mL
Energy	2060kJ
Protein	26.2g
Fat, Total	26.7g
- Saturated	5.6g
Carbohydrate	33.5g
- Sugars	23.0g
Dietary Fibre	6.6g
Sodium	130mg

Ingredients: Nuts 26% (Peanuts, Almonds), Plant Protein Blend (Soy Protein Crisps Isolated Soy Protein, Tapioca Starch, Salt, Soy Protein Isolate), Glucose, Seeds 11% (Sunflower, Pepitas, Sesame), Honey, Dark Choc Compound 9% (Sugar, Vegetable Oil, Cocoa Powder, Emulsifier Soy Lecithin, Sunflower Lecithin, Salt, Natural Flavour, Milk), Cranberries 7% (Sugar, Cranberries, Sunflower Oil), Sunflower Oil, Natural Flavours, Cinnamon.

☐ Full cream milk

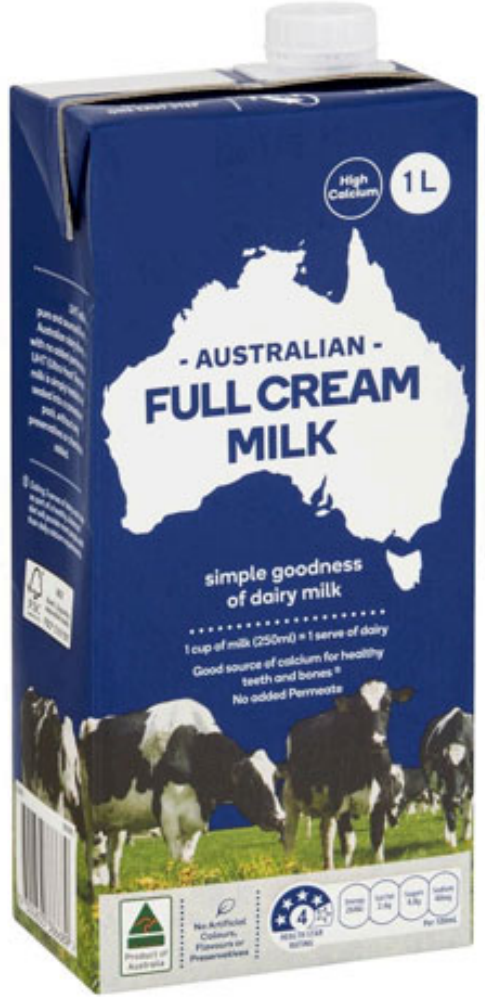

Nutrition Information	Quantity Per 100g/ 100mL
Energy	264kJ
Protein	3.3g
Fat, Total	3.2g
- Saturated	2.5g
Carbohydrate	4.8g
- Sugars	4.8g
Dietary Fibre	-
Sodium	40mg

Ingredients: Full Cream Milk.

☐ » Pasta

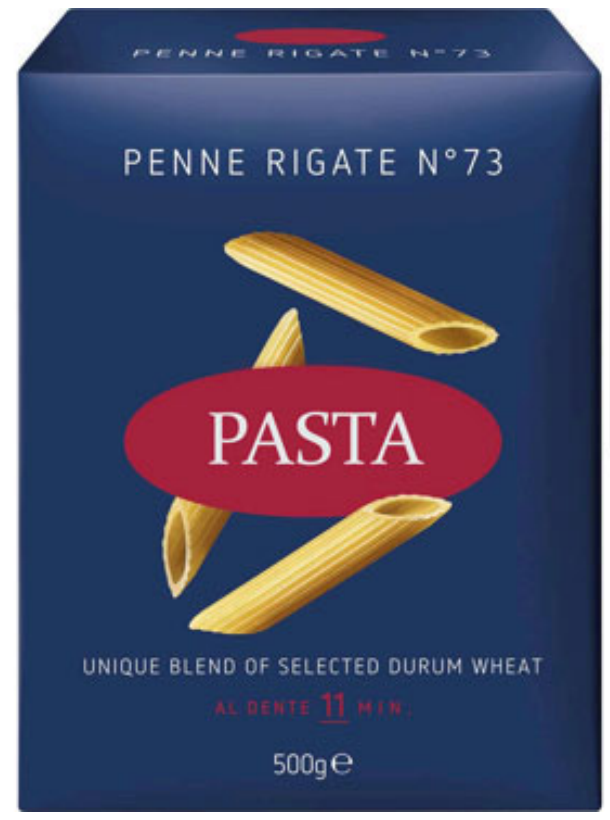

Nutrition Information	Quantity Per 100g/ 100mL
Energy	1520kJ
Protein	12.5g
Fat, Total	2.0g
- Saturated	0.5g
Carbohydrate	71.2g
- Sugars	3.5g
Dietary Fibre	3.0g
Sodium	5mg

Ingredients: Durum wheat semolina, Water.

☐ » Instant noodles

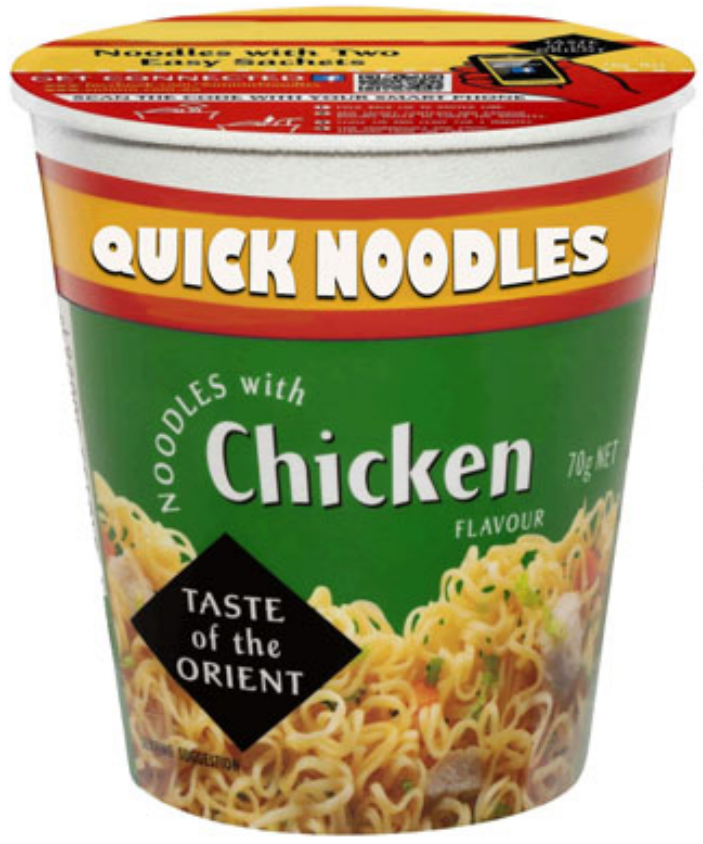

Nutrition Information	Quantity Per 100g/ 100mL
Energy	378kJ
Protein	1.7g
Fat, Total	3.6g
- Saturated	2.1g
Carbohydrate	11.3g
- Sugars	0.4g
Dietary Fibre	8.4g
Sodium	420mg

Ingredients: Noodles: Wheat Flour, Vegetable Oil (Antioxidant (320)), Salt.
Flavour Sachet: Salt, Flavour Enhancers (621, 635), Maltodextrin, Flavour, Anticaking Agent (551), Chicken Powder, Onion Powder, Herbs and Spice, Vegetable Oil (Antioxidant (307b)), Colours (150a, 100).
Vegetable Sachet: Dried Carrot, Textured Vegetable Protein, Dried Onion.

☐ 100% Fruit juice

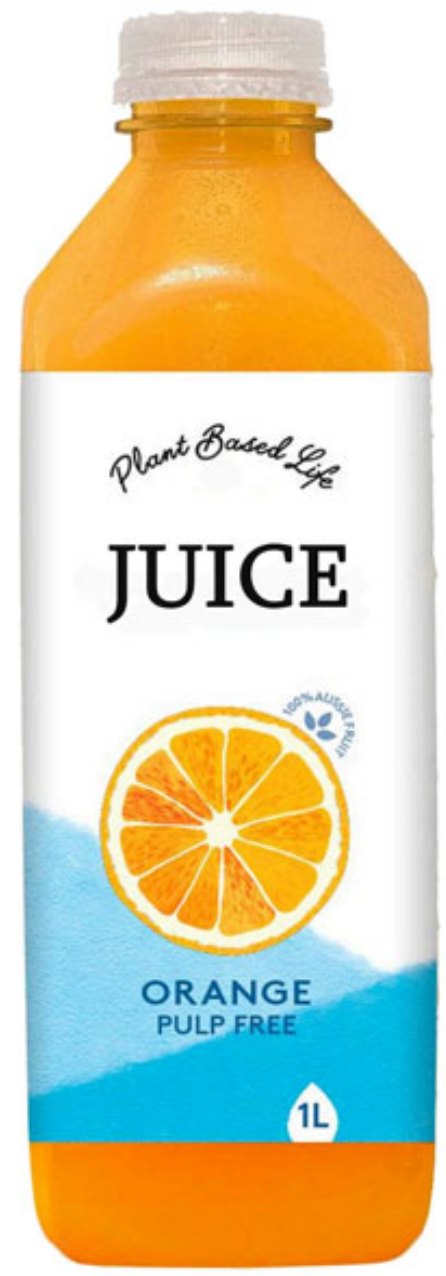

Nutrition Information	Quantity Per 100g/ 100mL
Energy	493kJ
Protein	1.2g
Fat, Total	0.1g
- Saturated	0.1g
Carbohydrate	27.2g
- Sugars	21.3g
Dietary Fibre	0.1g
Sodium	1.0mg

Ingredients: 100% Orange Juice.

☐ » Bolognese pasta sauce

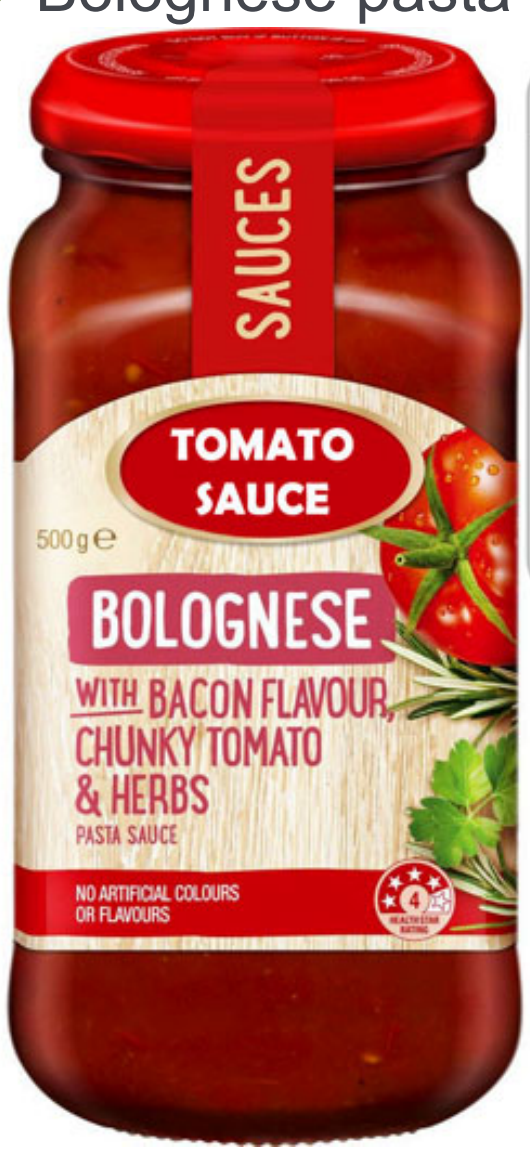

Nutrition Information	Quantity Per 100g/ 100mL
Energy	181kJ
Protein	1.7g
Fat, Total	0.2g
- Saturated	0.1g
Carbohydrate	7.6g
- Sugars	4.8g
Dietary Fibre	1.8g
Sodium	361mg

Ingredients: Tomato puree (78%) (from paste), diced tomatoes (9%), vegetables (onion, carrot), thickener (1442), sugar, yeast extract, garlic, salt, natural flavour, spices, herbs (parsley, oregano, rosemary, thyme).

☐ » Ready meal lasagne

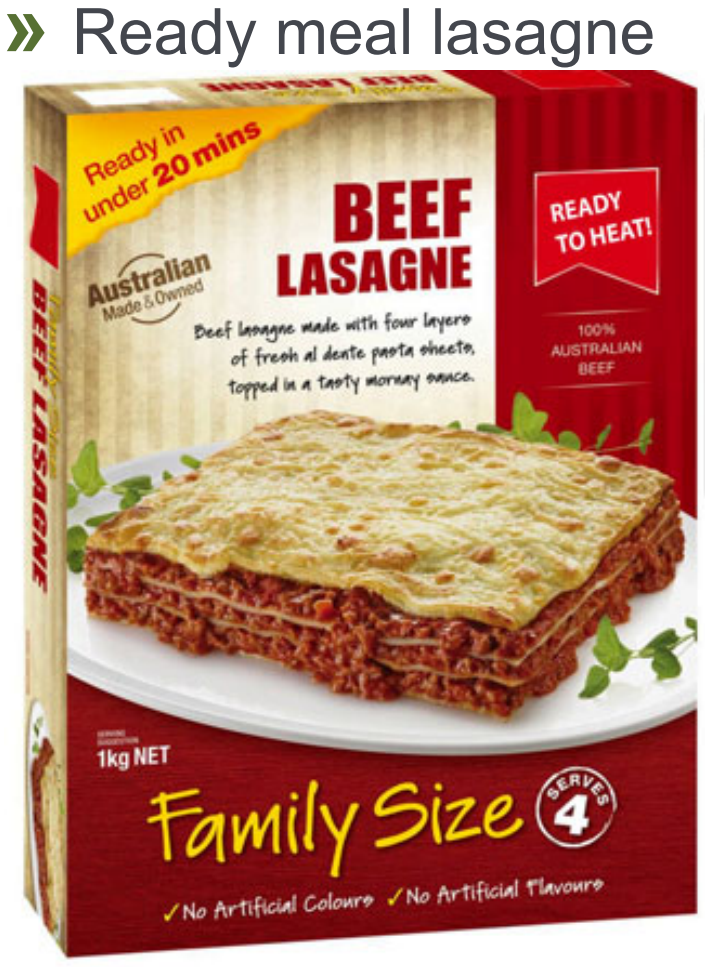

Nutrition Information	Quantity Per 100g/ 100mL
Energy	607kJ
Protein	5.3g
Fat, Total	6.2g
- Saturated	3.1g
Carbohydrate	16.1g
- Sugars	2.2g
Dietary Fibre	2.1g
Sodium	277mg

Ingredients: Water, Cooked Pasta (27%) [Wheat Semolina, Water], Milk, Beef (11%), Tomato Paste (Tomatoes, Food Acid (330), Cheese (Milk), Onion, Vegetable Spread (Soy), Thickener (1422), Wheat Flour, Carrot, Sugar, Salt, Spices, Herbs, Yeast Extract, Food Acid (330), Flavour Enhancers (635).

☐ » Plain yoghurt

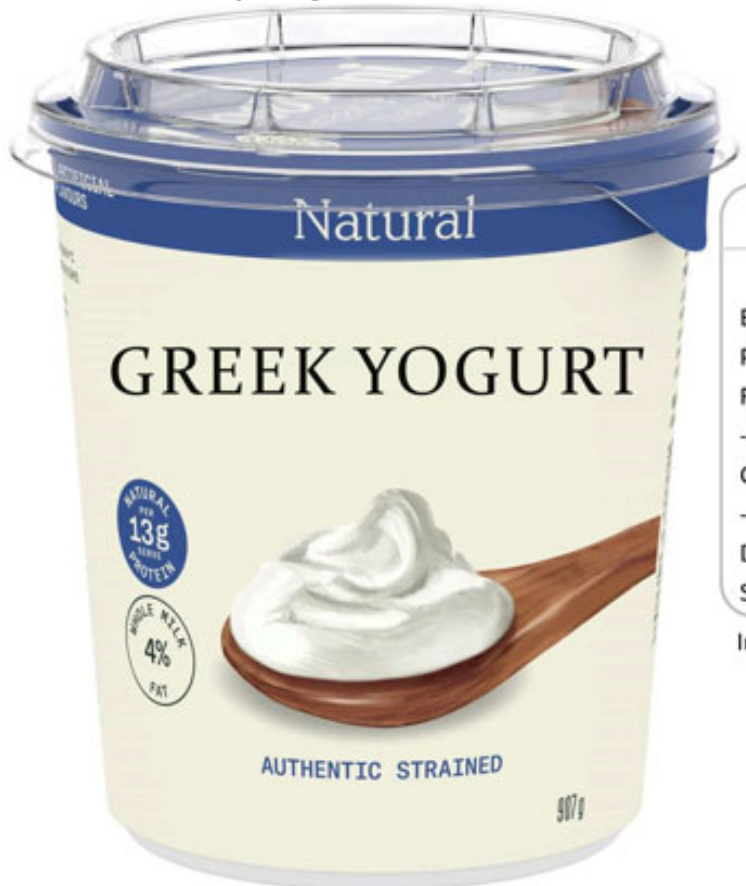

Nutrition Information

	Quantity Per 100g/ 100mL
Energy	362kJ
Protein	8.7g
Fat, Total	4.0g
- Saturated	2.6g
Carbohydrate	3.9g
- Sugars	2.7g
Dietary Fibre	-
Sodium	33mg

Ingredients: Milk, Cream, Live Yogurt Cultures.

☐ Chicken nuggets

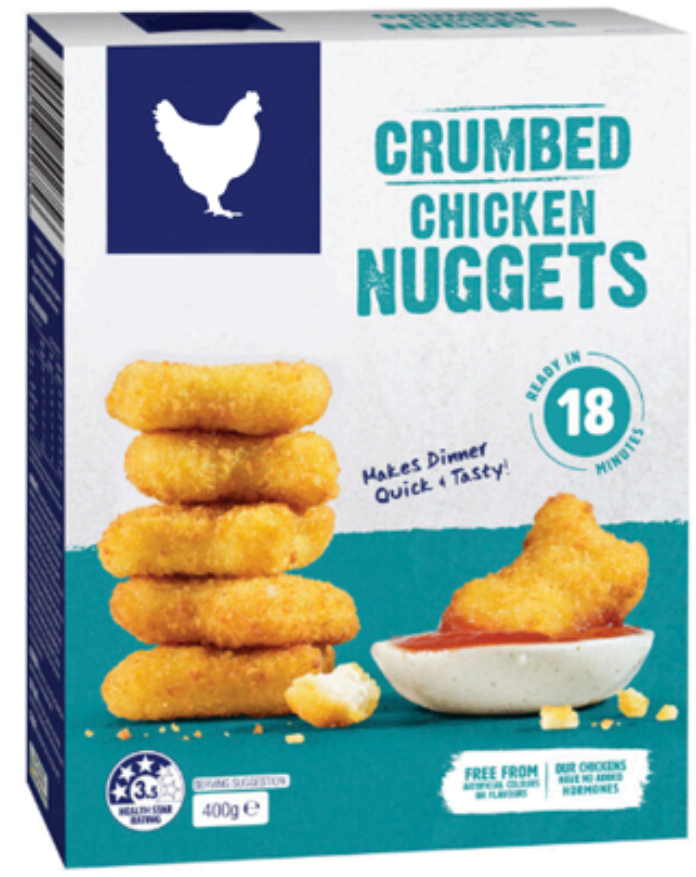

Nutrition Information

	Quantity Per 100g/ 100mL
Energy	962kJ
Protein	11.1g
Fat, Total	12.5g
- Saturated	6.2g
Carbohydrate	18.2g
- Sugars	1.9g
Dietary Fibre	1.0g
Sodium	500mg

Ingredients: Chicken Breast (41%), Coating (37%) Potato Flakes (Potato, Emulsifier (471), Mineral Salt (450) Preservative (223) (Sulphites), Food Acid (330)), Wheat Flour, Rice Flour, Salt, Emulsifier (481), Dextrose, Spice Extracts (160c, 100), Water, Wheat Gluten, Wheat Starch, Vegetable Oil, Thickener (1404), Mineral Salts (450, 500) Vegetable Gums (412, 415), Water, Vegetable Oil, Tapioca Starch (Sulphites), Soy Protein, Mineral Salts (451, 450), Dehydrated Vegetables (Onion, Garlic), Yeast Extract.

☐ Bolognese pasta sauce

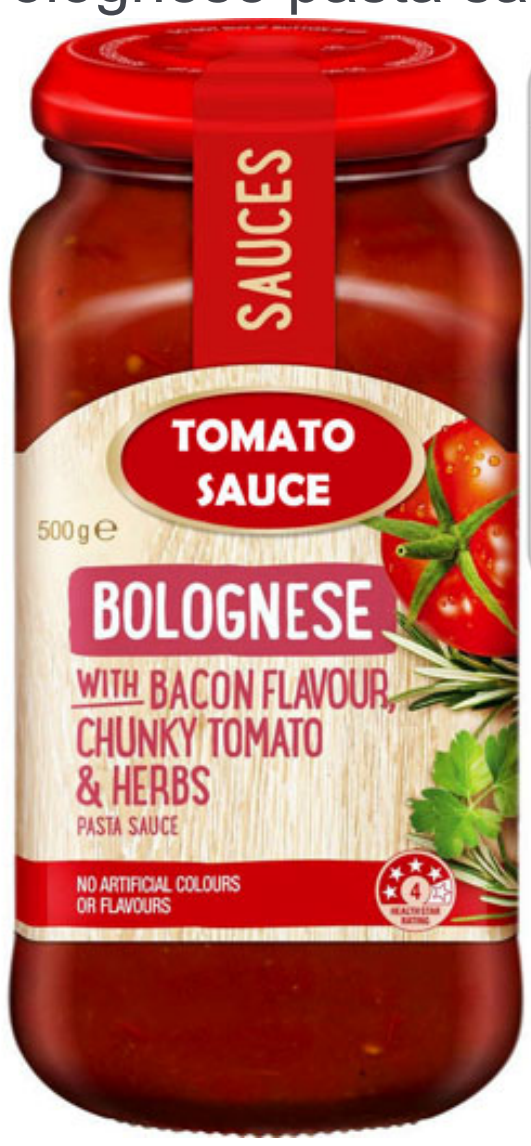

Nutrition Information

	Quantity Per 100g/ 100mL
Energy	181kJ
Protein	1.7g
Fat, Total	0.2g
- Saturated	0.1g
Carbohydrate	7.6g
- Sugars	4.8g
Dietary Fibre	1.8g
Sodium	361mg

Ingredients: Tomato puree (78%) (from paste), diced tomatoes (9%), vegetables (onion, carrot), thickener (1442), sugar, yeast extract, garlic, salt, natural flavour, spices, herbs (parsley, oregano, rosemary, thyme).

☐ Flavoured yoghurt

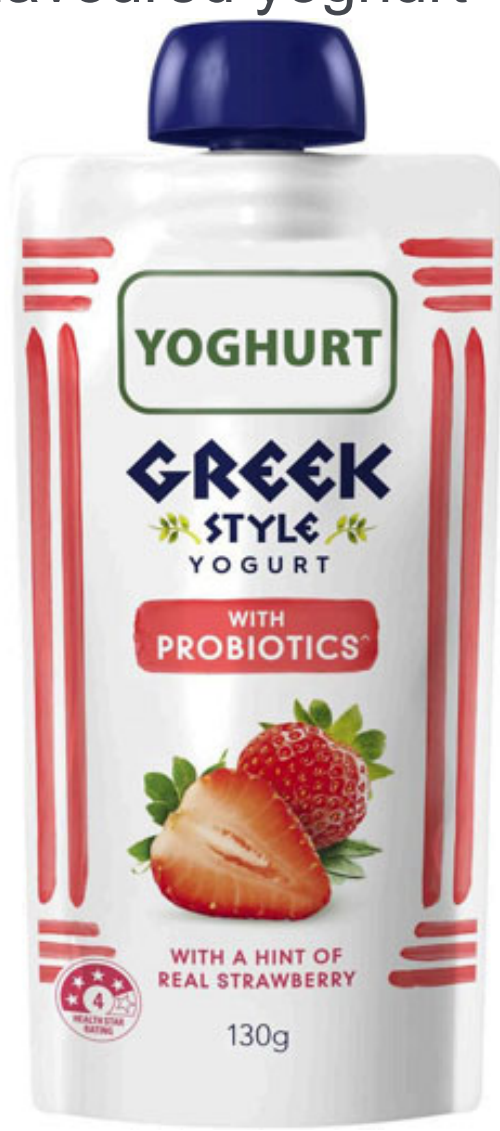

Nutrition Information

	Quantity Per 100g/ 100mL
Energy	376kJ
Protein	5.0g
Fat, Total	2.9g
- Saturated	1.9g
Carbohydrate	10.7g
- Sugars	10.2g
Dietary Fibre	0.0g
Sodium	50mg

Ingredients: Milk, Milk Solids, Strawberry (5%), (Puree, Reconstituted Juice), Water, Sugar, Cream (Milk), Thickener (Modified Starch), Natural Flavour, Natural Colour (Carrot Concentrate), Milk Minerals, Probiotic Cultures (Milk): B. lactis (BB-12), Yoghurt Cultures (Milk): S. Thermophilus, L. Bulgaricus.

Could you tell us what about the product made you think that it might have been ultra-processed? For instance, did you consider elements from the packaging or rely on what you already know about these foods? Your insights will help us understand your perspective better.

» Multi-grain packaged bread

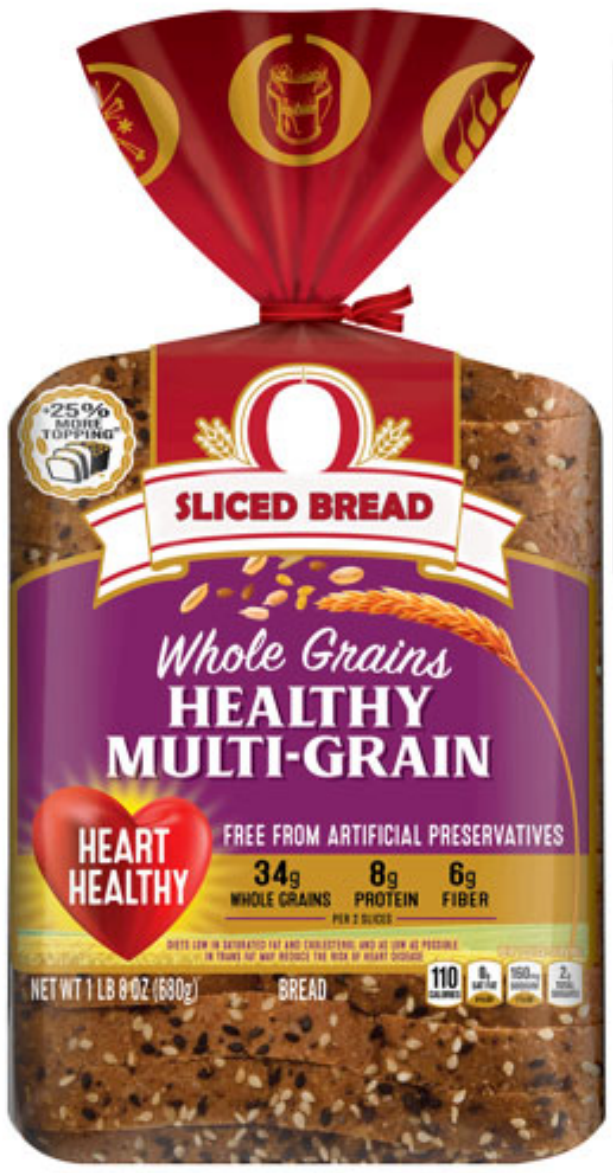

Nutrition Information

	Quantity Per 100g/ 100mL
Energy	1166kJ
Protein	11.6g
Fat, Total	5.8g
- Saturated	0.0g
Carbohydrate	46.2g
- Sugars	9.3g
Dietary Fibre	6.7g
Sodium	419mg

Ingredients: Whole Wheat Flour, Water, Sugar, Wheat Gluten, Brown And Golden Flaxseed, Yeast, Honey, Bulgur Wheat, Vegetable Oil (Soybean), Cornmeal, Oats, Brown Rice, Steel Cut Oats, Sunflower Seeds, Sea Salt, Cellulose Fibre, Molasses, Cultured Wheat Flour, Grain Vinegar, Natural Flavors, Soy Lecithin, Citric Acid, Oat Fibre.

» Ready meal lasagne

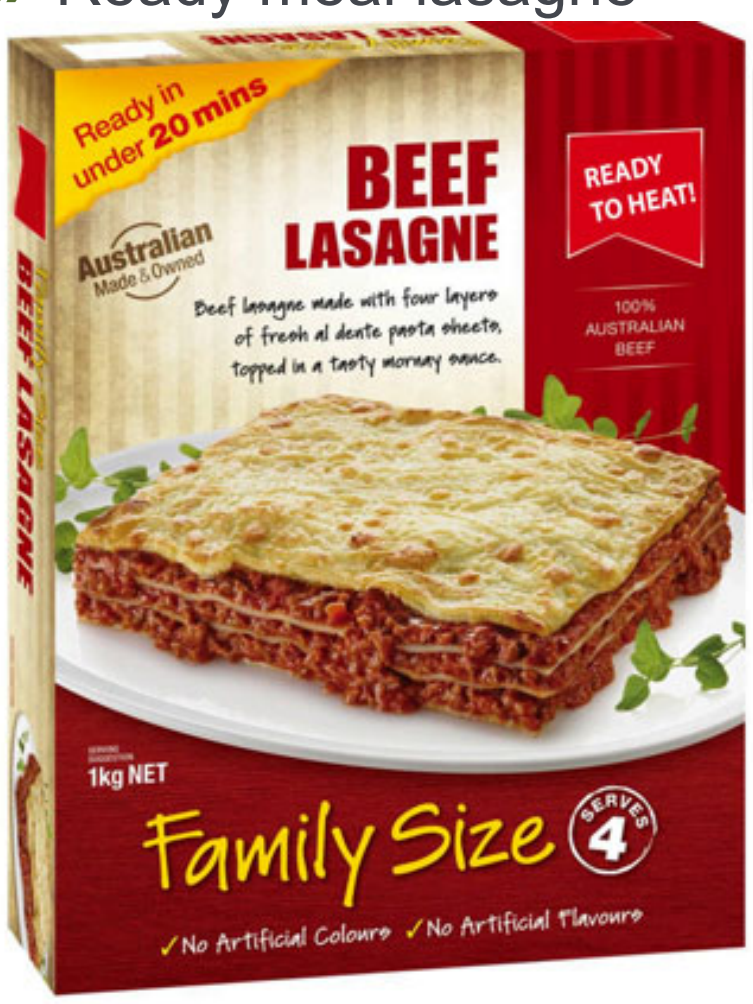

Nutrition Information

	Quantity Per 100g/ 100mL
Energy	607kJ
Protein	5.3g
Fat, Total	6.2g
- Saturated	3.1g
Carbohydrate	16.1g
- Sugars	2.2g
Dietary Fibre	2.1g
Sodium	277mg

Ingredients: Water, Cooked Pasta (27%) [Wheat Semolina, Water], Milk, Beef (11%), Tomato Paste (Tomatoes, Food Acid (330), Cheese (Milk), Onion, Vegetable Spread (Soy), Thickener (1422), Wheat Flour, Carrot, Sugar, Salt, Spices, Herbs, Yeast Extract, Food Acid (330), Flavour Enhancers (635).

» Instant noodles

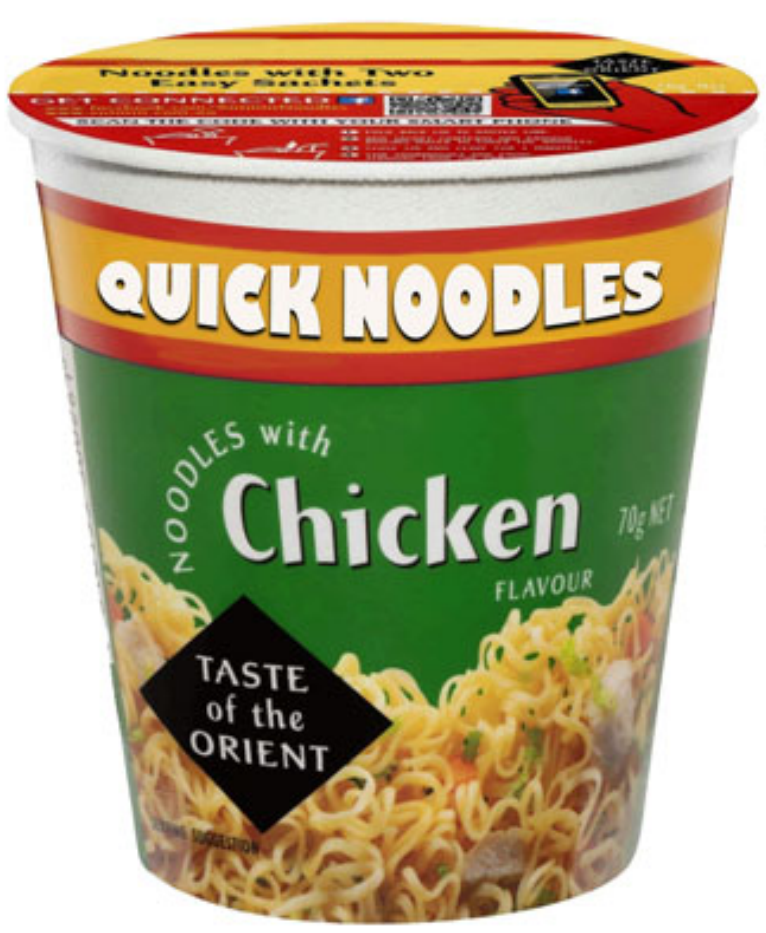

Nutrition Information

	Quantity Per 100g/ 100mL
Energy	378kJ
Protein	1.7g
Fat, Total	3.6g
- Saturated	2.1g
Carbohydrate	11.3g
- Sugars	0.4g
Dietary Fibre	8.4g
Sodium	420mg

Ingredients: Noodles: Wheat Flour, Vegetable Oil (Antioxidant (320)), Salt.
Flavour Sachet: Salt, Flavour Enhancers (621, 635), Maltodextrin, Flavour, Anticaking Agent (551), Chicken Powder, Onion Powder, Herbs and Spice, Vegetable Oil (Antioxidant (307b)), Colours (150a, 100).
Vegetable Sachet: Dried Carrot, Textured Vegetable Protein, Dried Onion.

» Bolognese pasta sauce

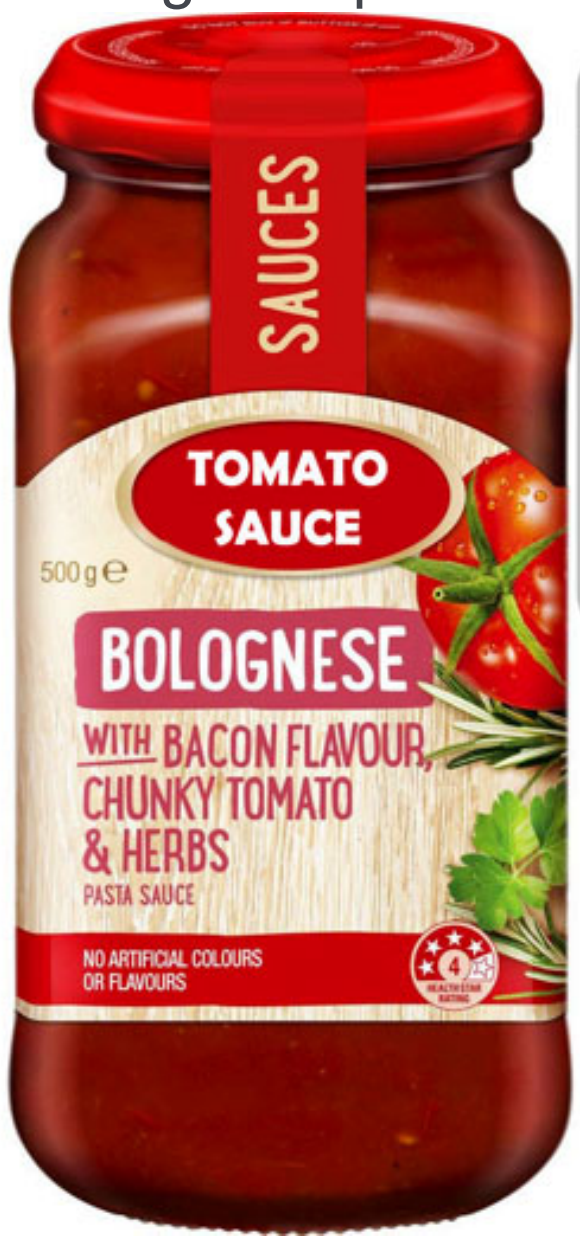

Nutrition Information

	Quantity Per 100g/ 100mL
Energy	181kJ
Protein	1.7g
Fat, Total	0.2g
- Saturated	0.1g
Carbohydrate	7.6g
- Sugars	4.8g
Dietary Fibre	1.8g
Sodium	361mg

Ingredients: Tomato puree (78%) (from paste), diced tomatoes (9%), vegetables (onion, carrot), thickener (1442), sugar, yeast extract, garlic, salt, natural flavour, spices, herbs (parsley, oregano, rosemary, thyme).

» Cereal bar

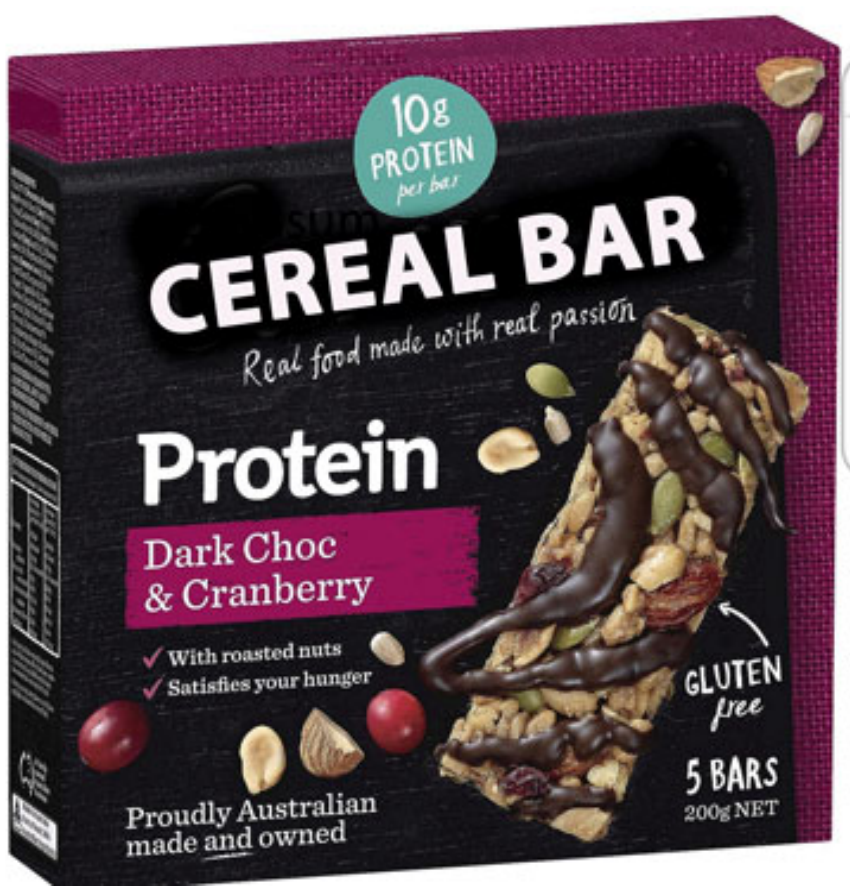

Nutrition Information

	Quantity Per 100g/ 100mL
Energy	2060kJ
Protein	26.2g
Fat, Total	26.7g
- Saturated	5.6g
Carbohydrate	33.5g
- Sugars	23.0g
Dietary Fibre	6.6g
Sodium	130mg

Ingredients: Nuts 26% (Peanuts, Almonds), Plant Protein Blend (Soy Protein Crisps Isolated Soy Protein, Tapioca Starch, Salt, Soy Protein Isolate), Glucose, Seeds 11% (Sunflower, Pepitas, Sesame), Honey, Dark Choc Compound 9% (Sugar, Vegetable Oil, Cocoa Powder, Emulsifier Soy Lecithin, Sunflower Lecithin, Salt, Natural Flavour, Milk), Cranberries 7% (Sugar, Cranberries, Sunflower Oil), Sunflower Oil, Natural Flavours, Cinnamon.

» Flavoured water

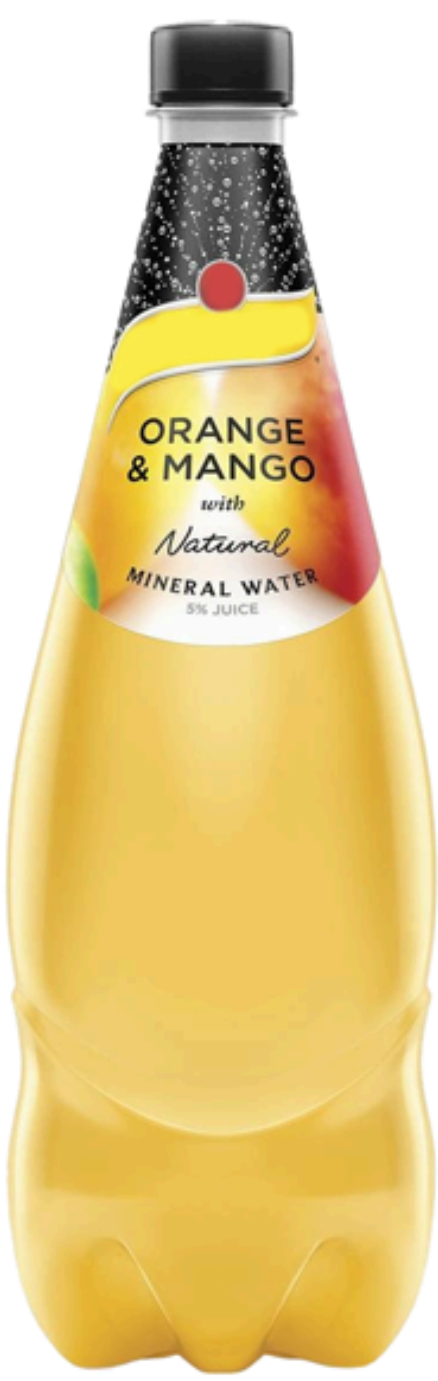

Nutrition Information

	Quantity Per 100g/ 100mL
Energy	65kJ
Protein	0.0g
Fat, Total	0.0g
- Saturated	0.0g
Carbohydrate	3.5g
- Sugars	3.4g
Dietary Fibre	-
Sodium	6mg

Ingredients: Carbonated Water (Mineral Water 80%, Purified Water 11%), Reconstituted Juices (Orange 4%, Mango 1%) , Sugar, Natural Flavours, Food Acids (Citric Acid , Ascorbic Acid), Preservatives (211, 202, 223 (SULPHITES)), Natural Colours (Carminic Acid, Lutein) , Antioxidant (385).

» Chicken nuggets

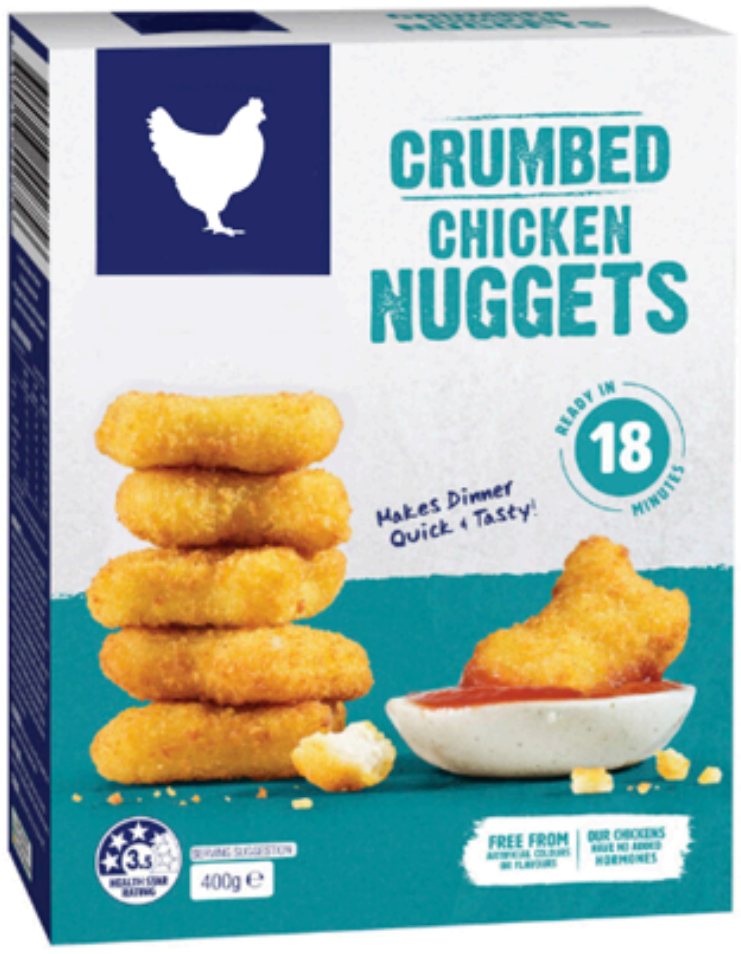

Nutrition Information

	Quantity Per 100g/ 100mL
Energy	962kJ
Protein	11.1g
Fat, Total	12.5g
- Saturated	6.2g
Carbohydrate	18.2g
- Sugars	1.9g
Dietary Fibre	1.0g
Sodium	500mg

Ingredients: Chicken Breast (41%), Coating (37%) Potato Flakes (Potato, Emulsifier (471), Mineral Salt (450) Preservative (223) (Sulphites), Food Acid (330)), Wheat Flour, Rice Flour, Salt, Emulsifier (481), Dextrose, Spice Extracts (160c, 100), Water, Wheat Gluten, Wheat Starch, Vegetable Oil, Thickener (1404), Mineral Salts (450, 500) Vegetable Gums (412, 415), Water, Vegetable Oil, Tapioca Starch (Sulphites), Soy Protein, Mineral Salts (451, 450), Dehydrated Vegetables (Onion, Garlic), Yeast Extract.

» Flavoured yoghurt

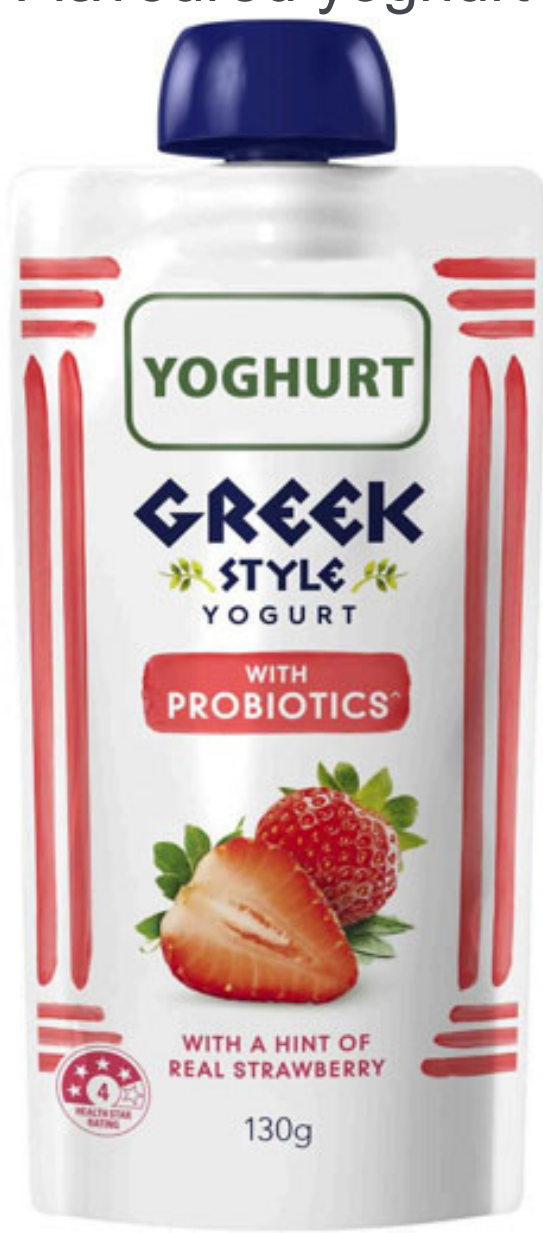

Nutrition Information

	Quantity Per 100g/ 100mL
Energy	376kJ
Protein	5.0g
Fat, Total	2.9g
- Saturated	1.9g
Carbohydrate	10.7g
- Sugars	10.2g
Dietary Fibre	0.0g
Sodium	50mg

Ingredients: Milk, Milk Solids, Strawberry (5%), (Puree, Reconstituted Juice), Water, Sugar, Cream (Milk), Thickener (Modified Starch), Natural Flavour, Natural Colour (Carrot Concentrate), Milk Minerals, Probiotic Cultures (Milk): B. lactis (BB-12), Yoghurt Cultures (Milk): S. Thermophilus, L. Bulgaricus.

» Chocolate block

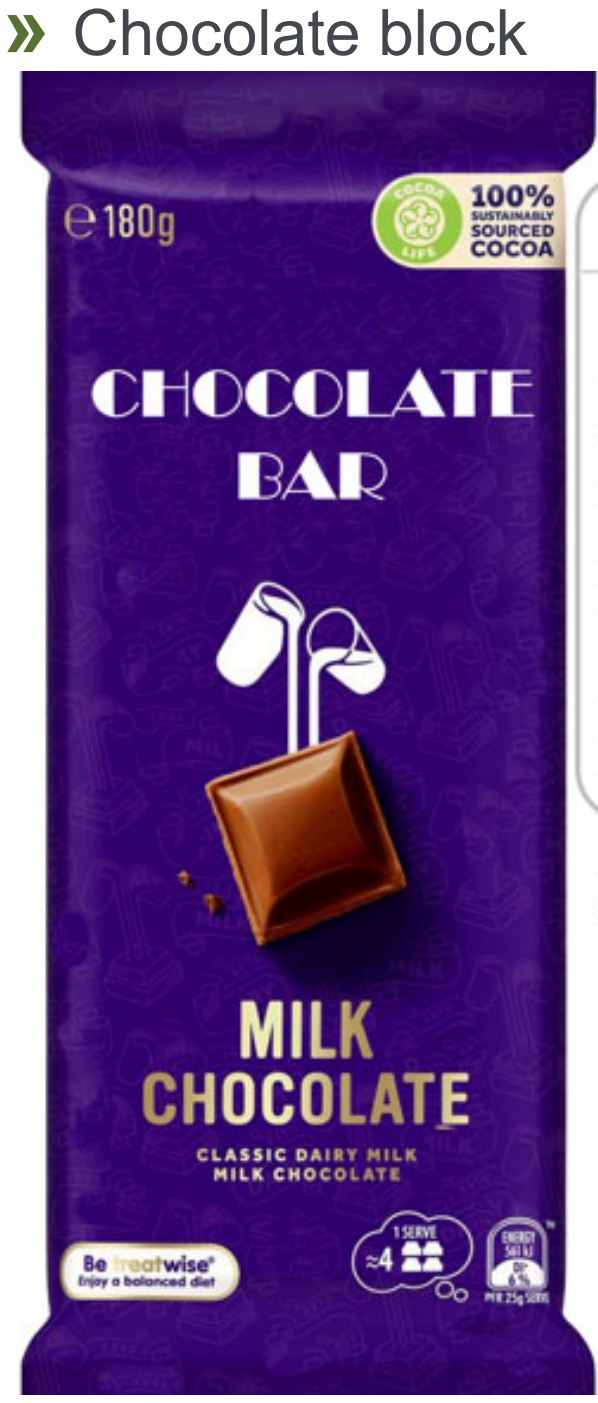

Nutrition Information

	Quantity Per 100g/ 100mL
Energy	2240kJ
Protein	7.4g
Fat, Total	30.5g
- Saturated	18.2g
Carbohydrate	57.0g
- Sugars	55.2g
Dietary Fibre	1.0g
Sodium	83mg

Ingredients: Full Cream Milk, Sugar, Cocoa Butter, Cocoa Mass, Milk Solids, Emulsifiers (Soy Lecithin, 476), Flavours.

» Fast food burger

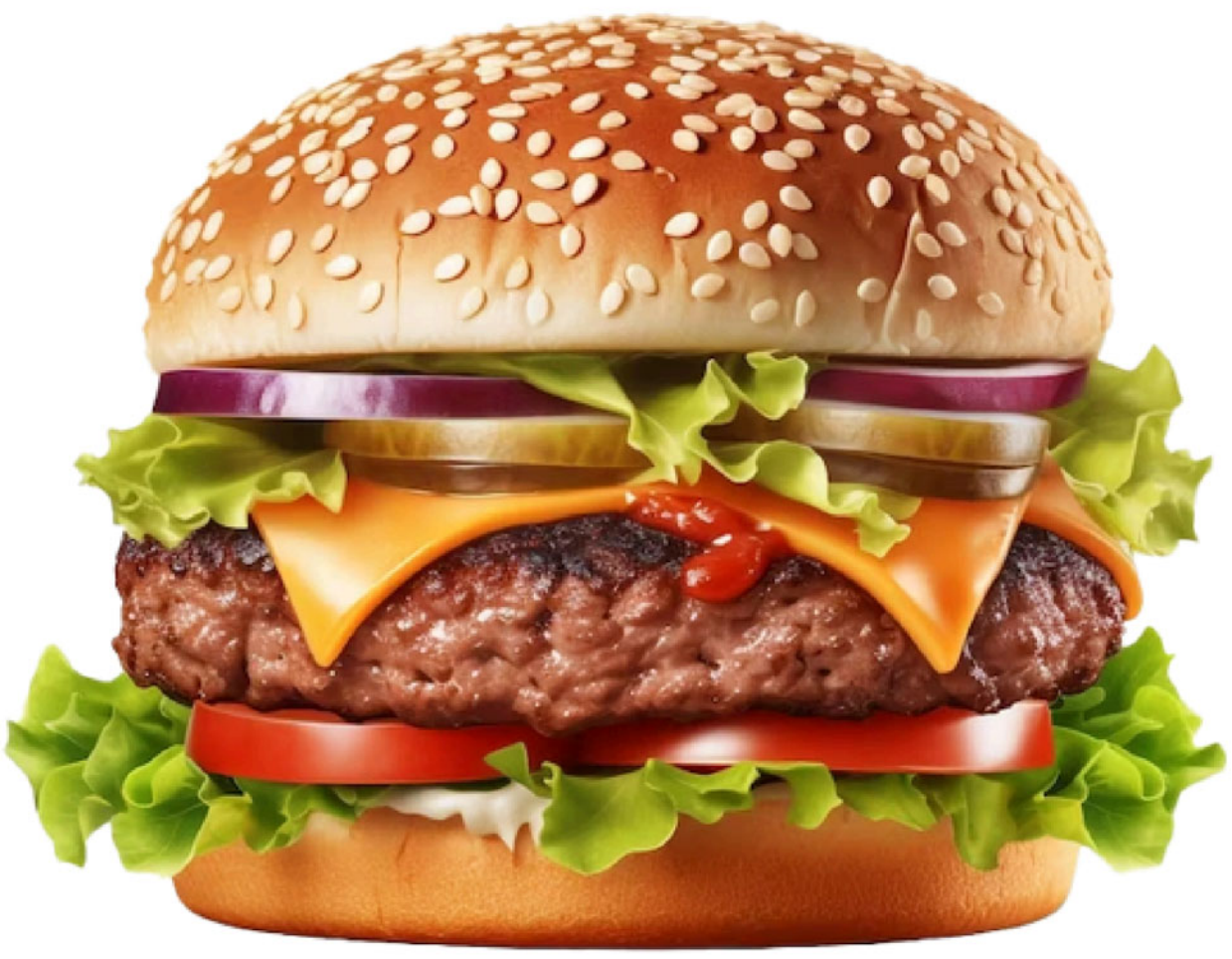

» Full cream milk

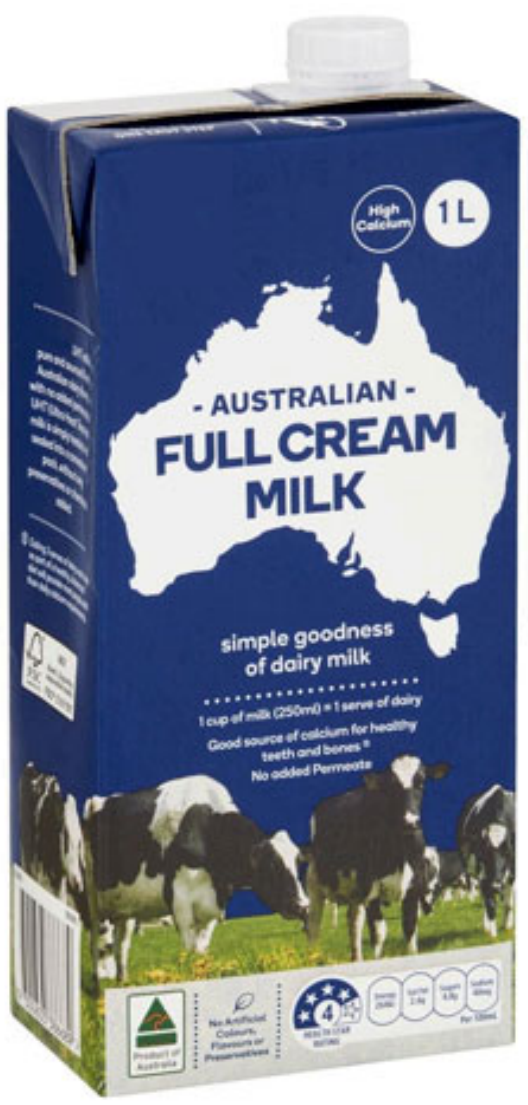

Nutrition Information

	Quantity Per 100g/ 100mL
Energy	264kJ
Protein	3.3g
Fat, Total	3.2g
- Saturated	2.5g
Carbohydrate	4.8g
- Sugars	4.8g
Dietary Fibre	-
Sodium	40mg

Ingredients: Full Cream Milk.

» Canned corn

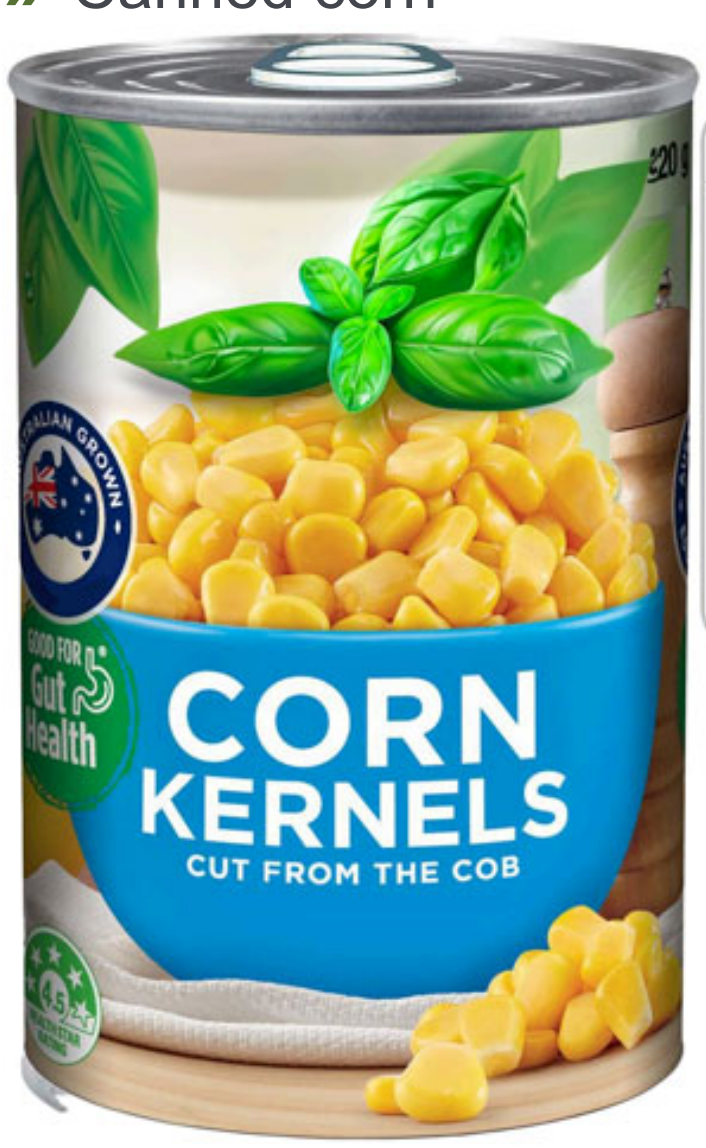

Nutrition Information

	Quantity Per 100g/ 100mL
Energy	275kJ
Protein	2.5g
Fat, Total	1.9g
- Saturated	0.4g
Carbohydrate	8.2g
- Sugars	3.2g
Dietary Fibre	2.6g
Sodium	241mg

Ingredients: Sweet corn (58%), water, salt.

» Muesli

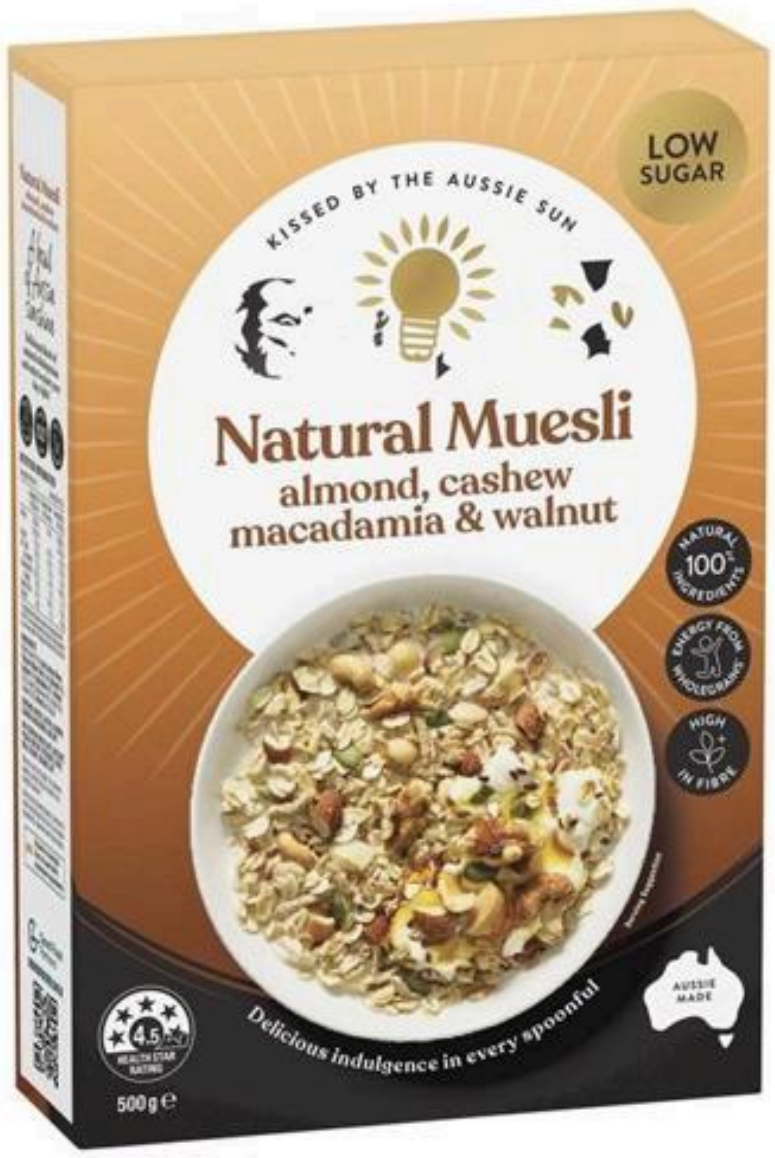

Nutrition Information

	Quantity Per 100g/100mL
Energy	1700kJ
Protein	13.0g
Fat, Total	16.8g
- Saturated	2.3g
Carbohydrate	49.0g
- Sugars	1.7g
Dietary Fibre	9.6g
Sodium	8.0mg

Ingredients: Whole Grains (75%) Oats, Barley, Wheat, Triticale (Wheat, Rye), Nuts Almond (5%), Cashew (5%), Macadamia (2%) Walnut (2%), Seeds Sunflower (4%), Pepita (2%), Linseed (2%), Wheat Bran Straws.

» Cheese block

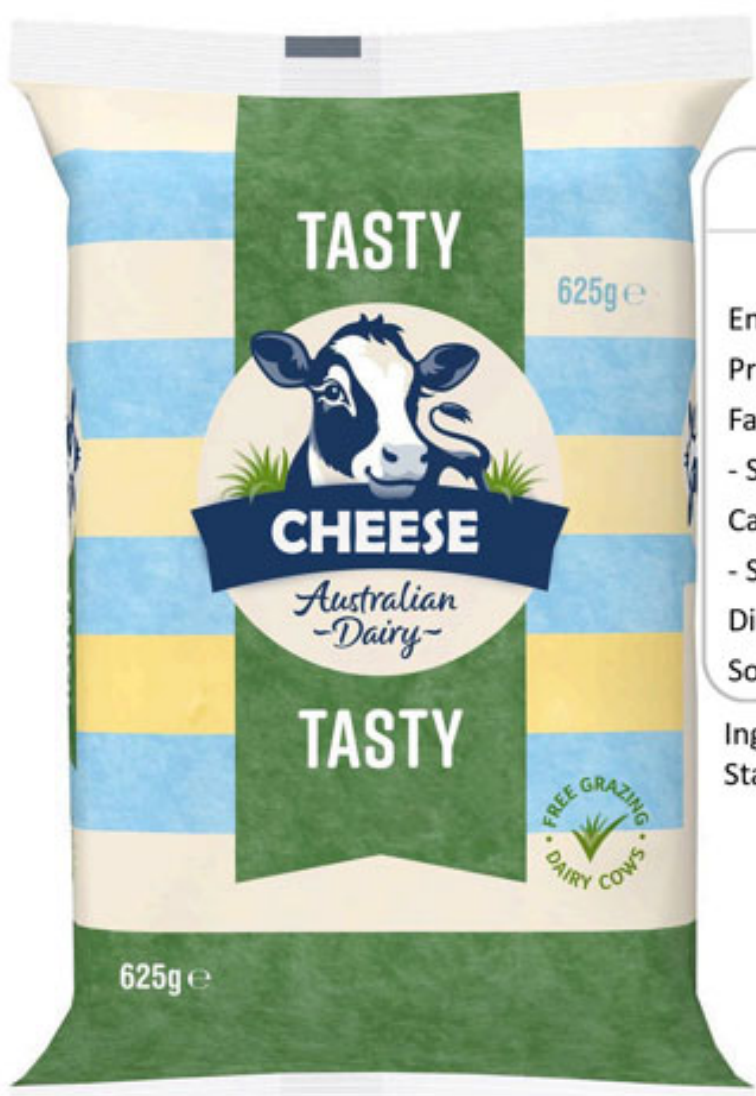

Nutrition Information

	Quantity Per 100g/ 100mL
Energy	1730kJ
Protein	24.3g
Fat, Total	35.0g
- Saturated	23.9g
Carbohydrate	<1g
- Sugars	<1g
Dietary Fibre	-
Sodium	633mg

Ingredients: Cheese (Pasteurised Milk, Salt, Starter Cultures, Enzyme (Non-Animal Rennet)).

» Plain yoghurt

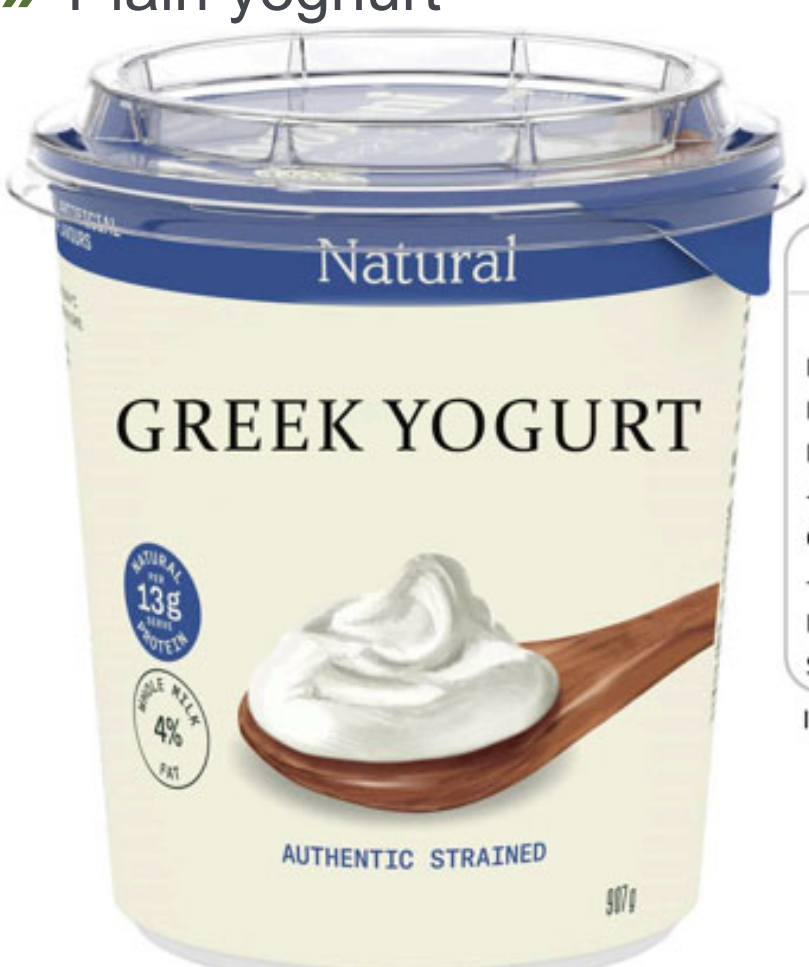

Nutrition Information

	Quantity Per 100g/ 100mL
Energy	362kJ
Protein	8.7g
Fat, Total	4.0g
- Saturated	2.6g
Carbohydrate	3.9g
- Sugars	2.7g
Dietary Fibre	-
Sodium	33mg

Ingredients: Milk, Cream, Live Yogurt Cultures.

» Canned fish

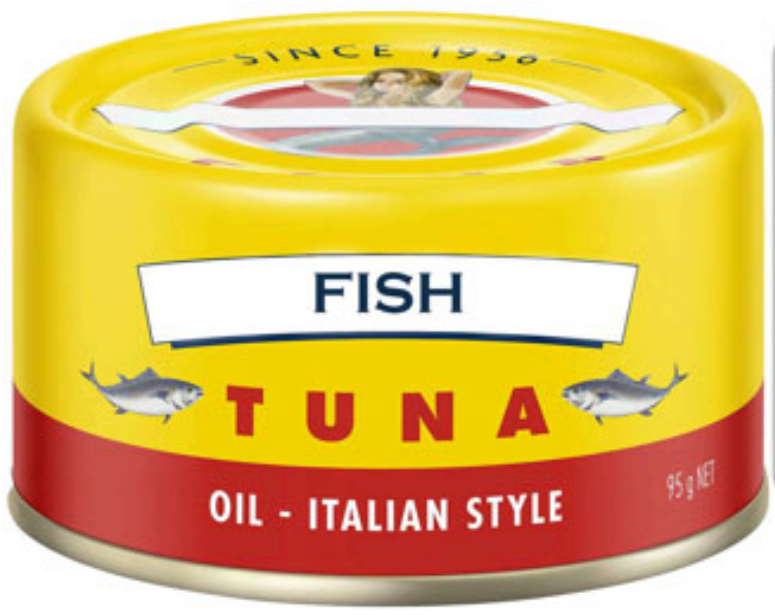

Nutrition Information

	Quantity Per 100g/ 100mL
Energy	809kJ
Protein	26.0g
Fat, Total	9.9g
- Saturated	1.2g
Carbohydrate	1.0g
- Sugars	1.0g
Dietary Fibre	-
Sodium	519mg

Ingredients: Pole & Line Caught Yellowfin Tuna (Fish)(73.5%), Oil (Sunflower Oil And Extra Virgin Olive Oil) (25%), Sea Salt.

» Bakery white bread

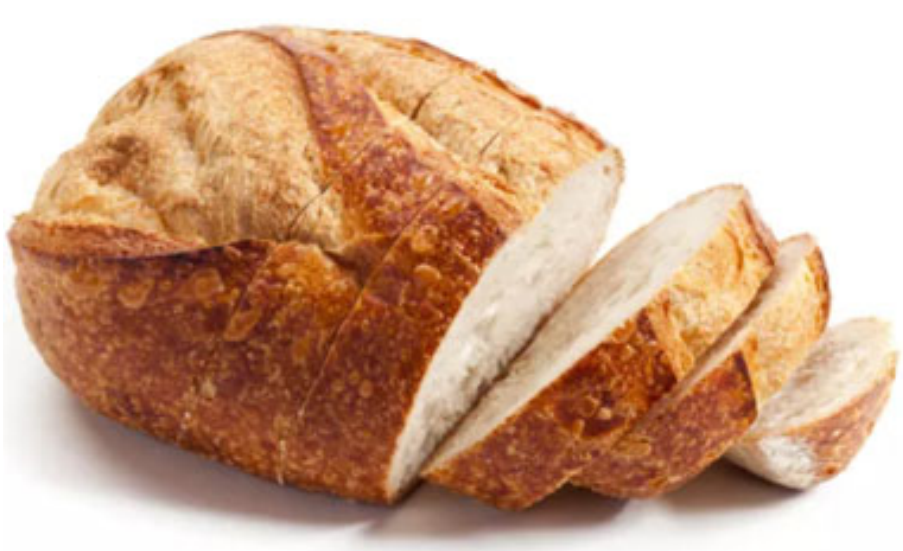

Nutrition Information

	Quantity Per 100g/ 100mL
Energy	1040kJ
Protein	9.3g
Fat, Total	1.4g
- Saturated	0.3g
Carbohydrate	45.6g
- Sugars	2.7g
Dietary Fibre	2.6g
Sodium	440mg

Ingredients: Wheat Flour, Water, Sourdough Culture (Water, Wheat Flour, Whole meal Wheat Flour), Whole meal Wheat Flour, Iodised Salt, Yeast, Vitamins (Thiamin, Folic Acid).

» Pasta

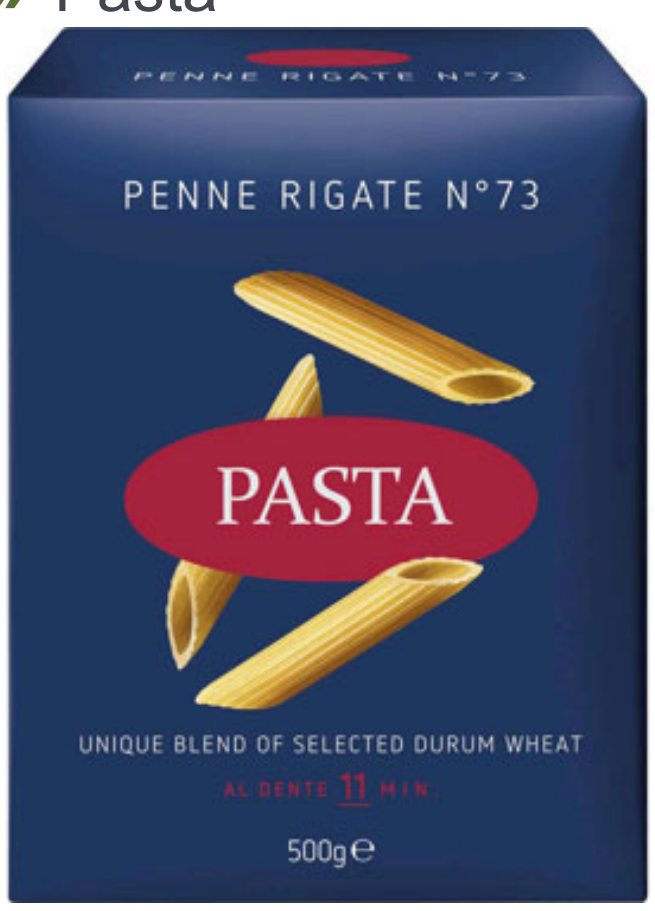

Nutrition Information

	Quantity Per 100g/ 100mL
Energy	1520kJ
Protein	12.5g
Fat, Total	2.0g
- Saturated	0.5g
Carbohydrate	71.2g
- Sugars	3.5g
Dietary Fibre	3.0g
Sodium	5mg

Ingredients: Durum wheat semolina, Water.

» Tortilla strips corn

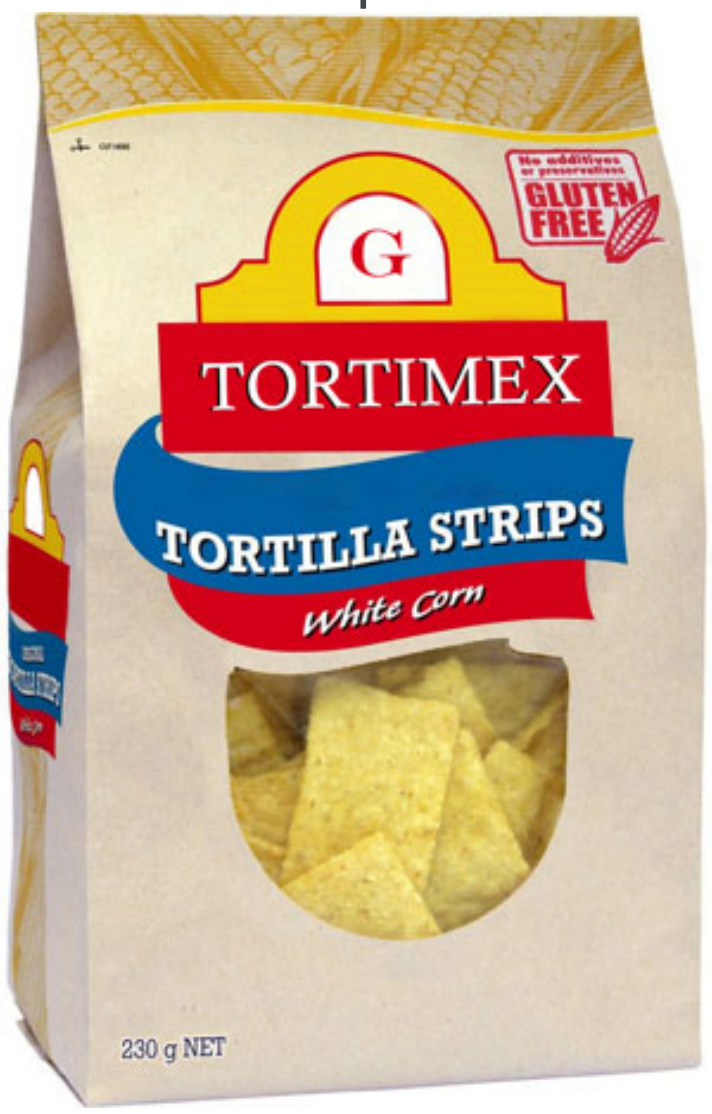

Nutrition Information

	Quantity Per 100g/ 100mL
Energy	2140kJ
Protein	6.3g
Fat, Total	26.5g
- Saturated	2.3g
Carbohydrate	59.2g
- Sugars	1g
Dietary Fibre	2.1g
Sodium	330mg

Ingredients: White Corn Flour (76%), Vegetable Oil , Water, Salt.

» 100% Fruit juice

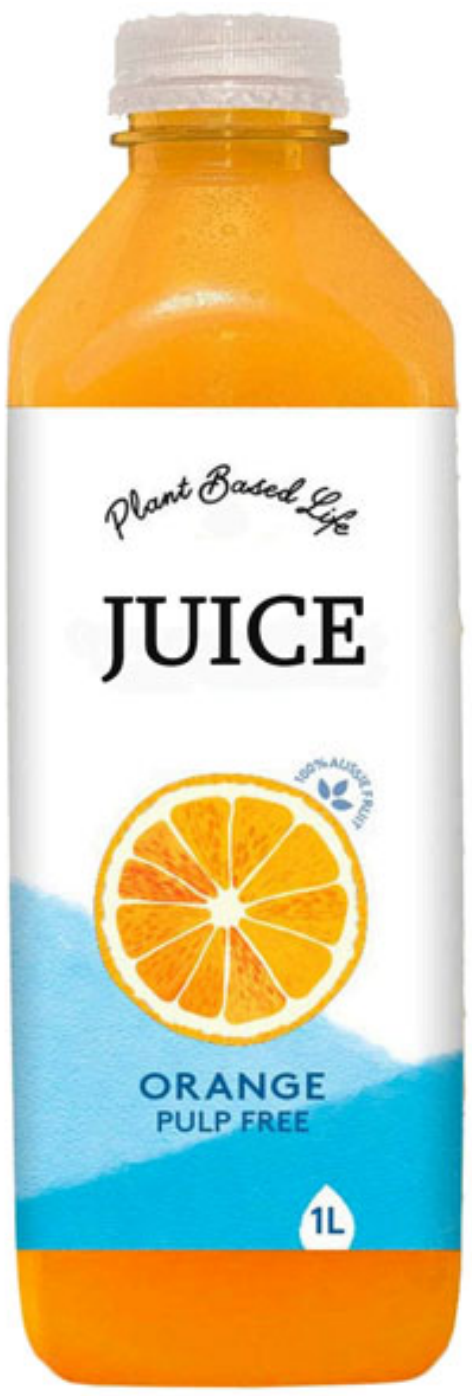

Nutrition Information

	Quantity Per 100g/ 100mL
Energy	493kJ
Protein	1.2g
Fat, Total	0.1g
- Saturated	0.1g
Carbohydrate	27.2g
- Sugars	21.3g
Dietary Fibre	0.1g
Sodium	1.0mg

Ingredients: 100% Orange Juice.

Now we are interested in your thoughts on how foods are different from each other. In the next four screens you will see a series of foods presented in groups of three. We would like you to compare and contrast the two products on the top with the one on the bottom.

There are no right or wrong answers, we would like to know more about how you think the various products differ.

Please feel free to think broadly, for example about the specific characteristics of the

foods, how you might use them, any positive and/or negative aspects of these products, or anything else that comes to mind.

In your view, how do the two products on the top differ from the one on the bottom? Please include both the top and bottom products in your response.

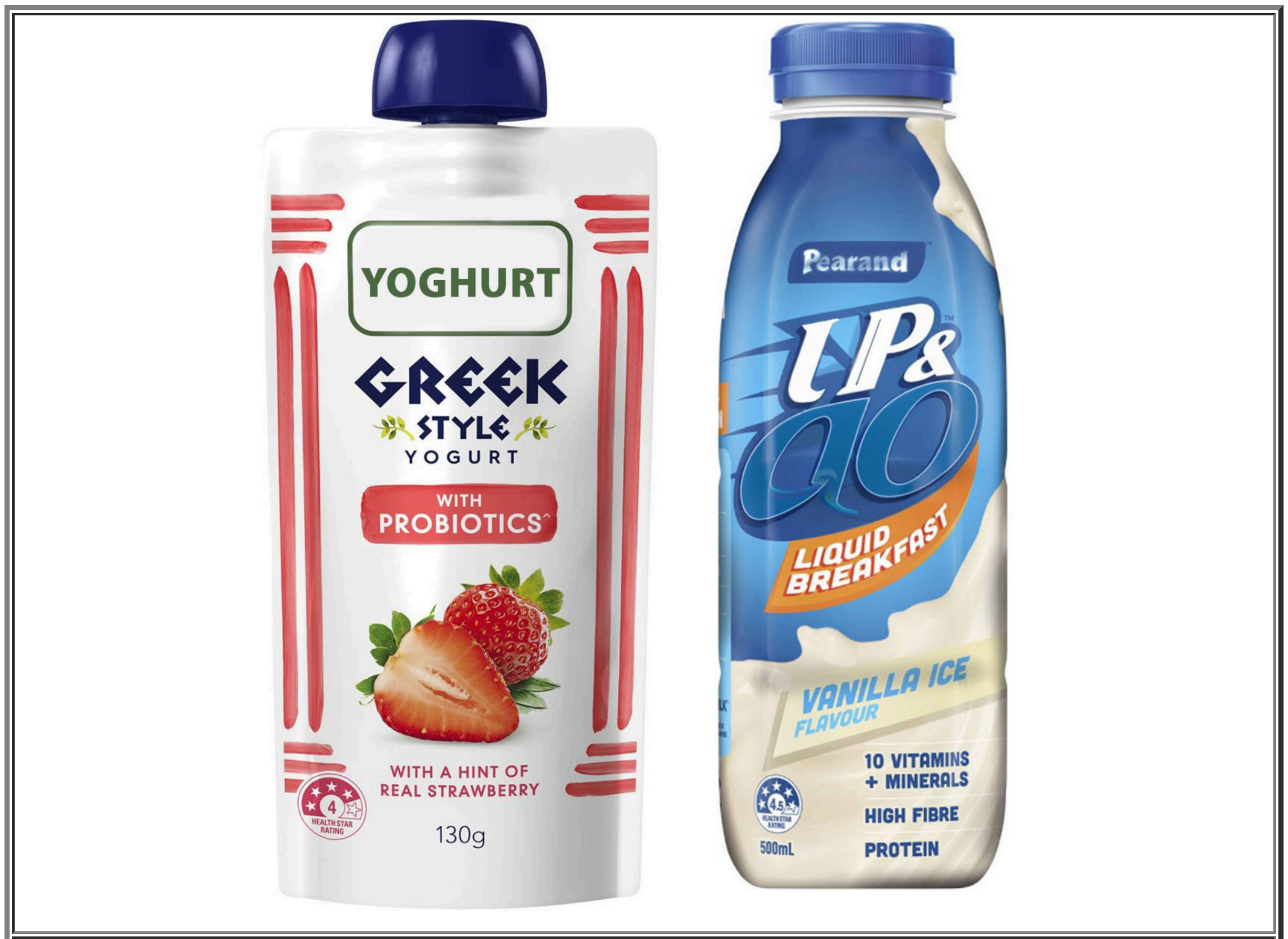

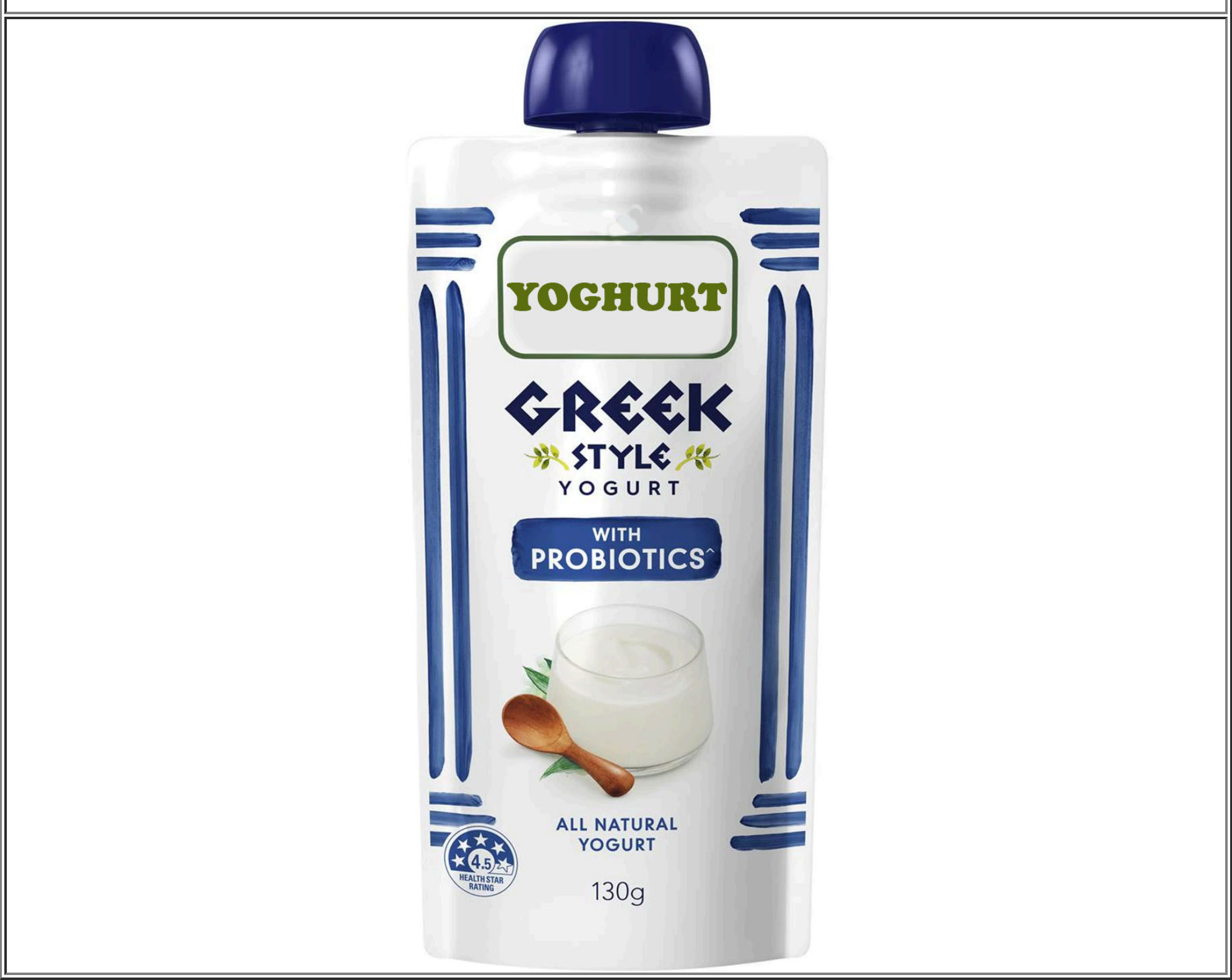

In your view, how do the two products on the top differ from the one on the bottom? Please include both the top and bottom products in your response.

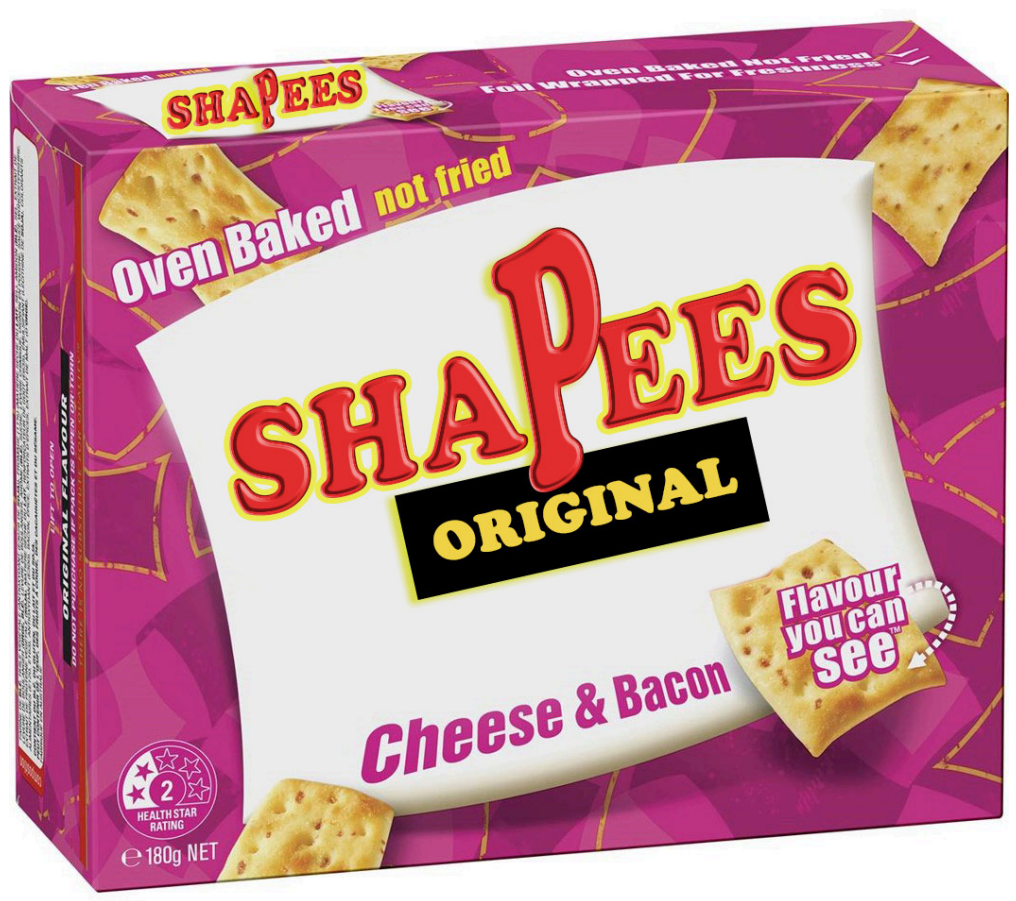

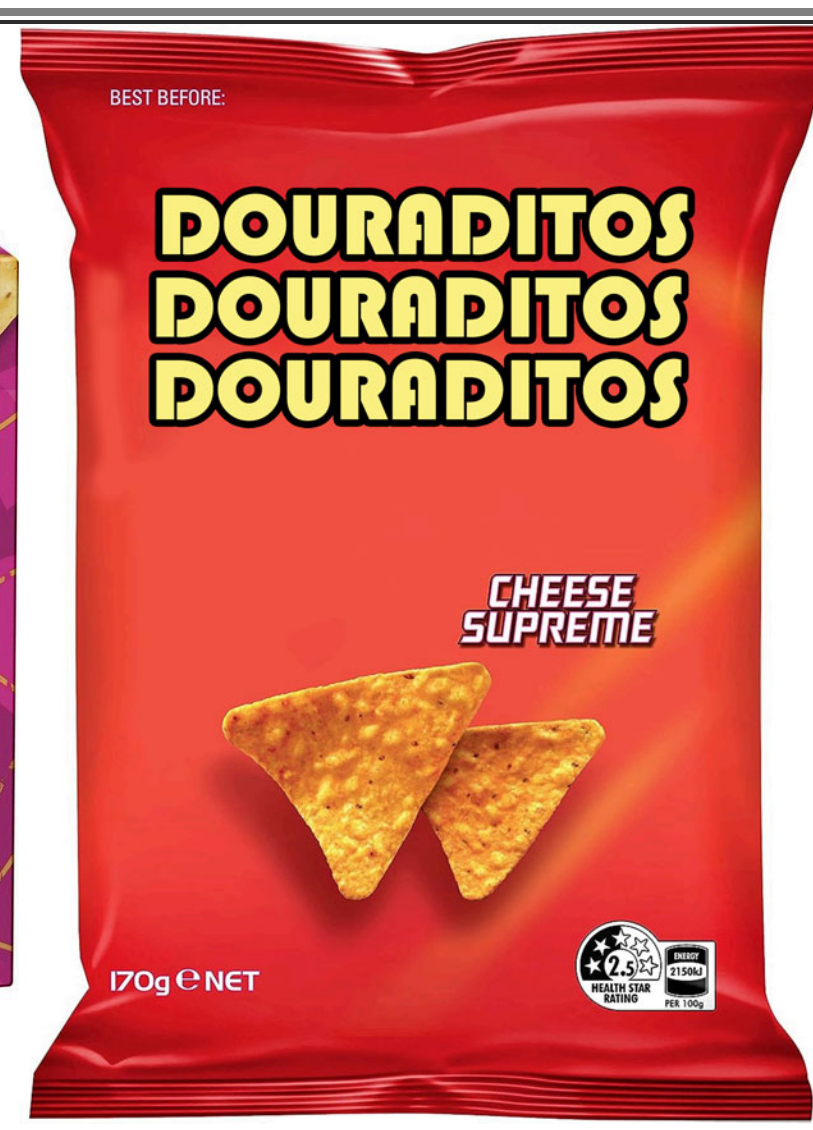

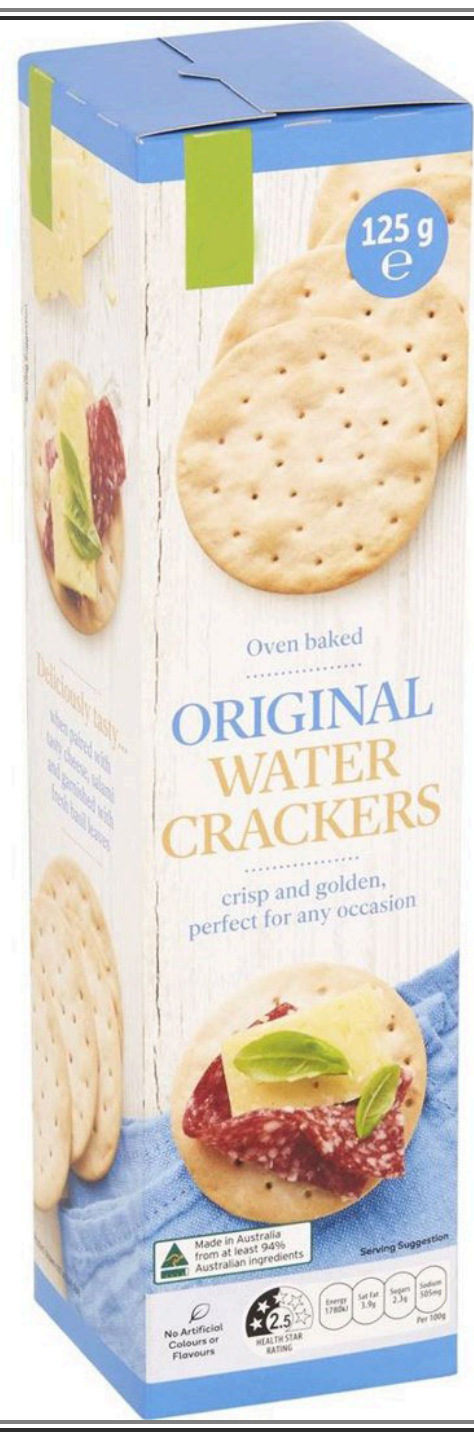

In your view, how do the two products on the top differ from the one on the bottom? Please include both the top and bottom products in your response.

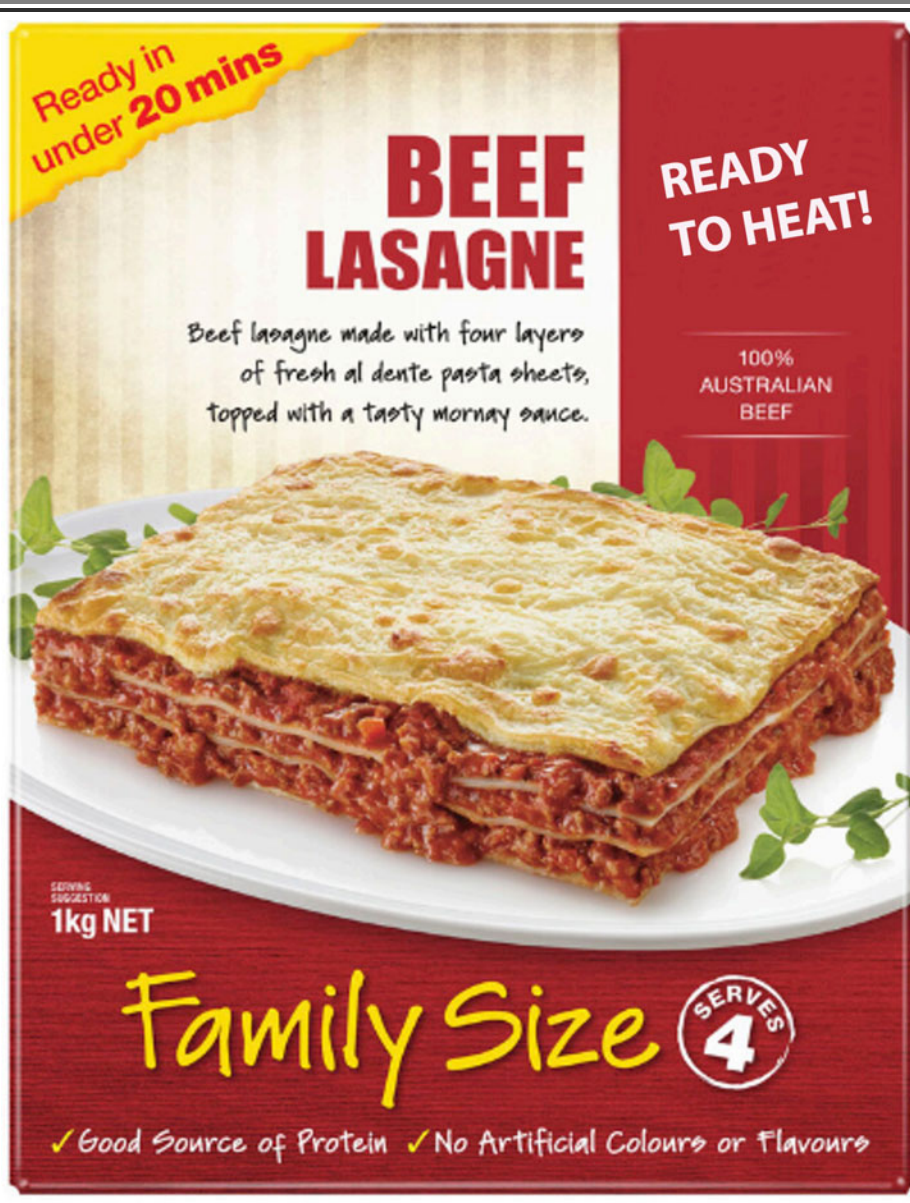

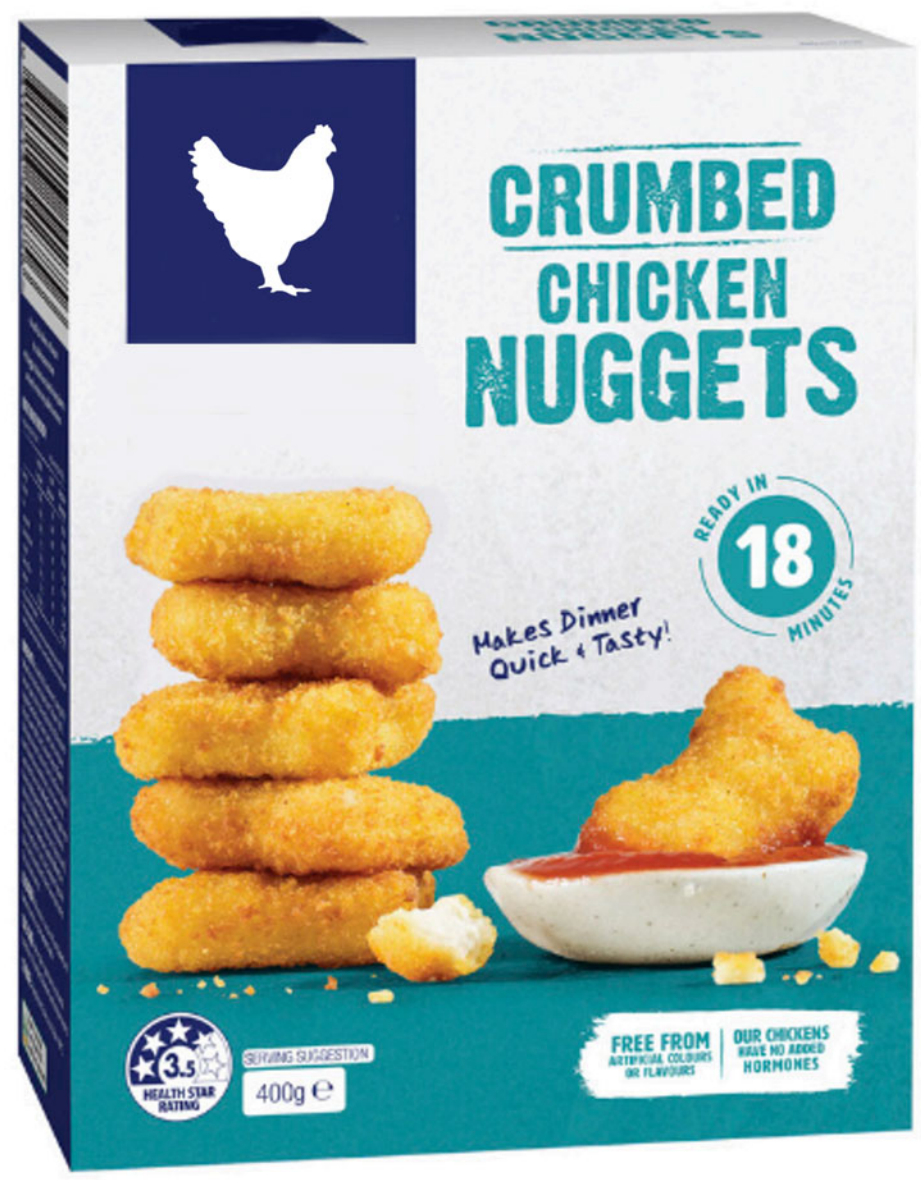

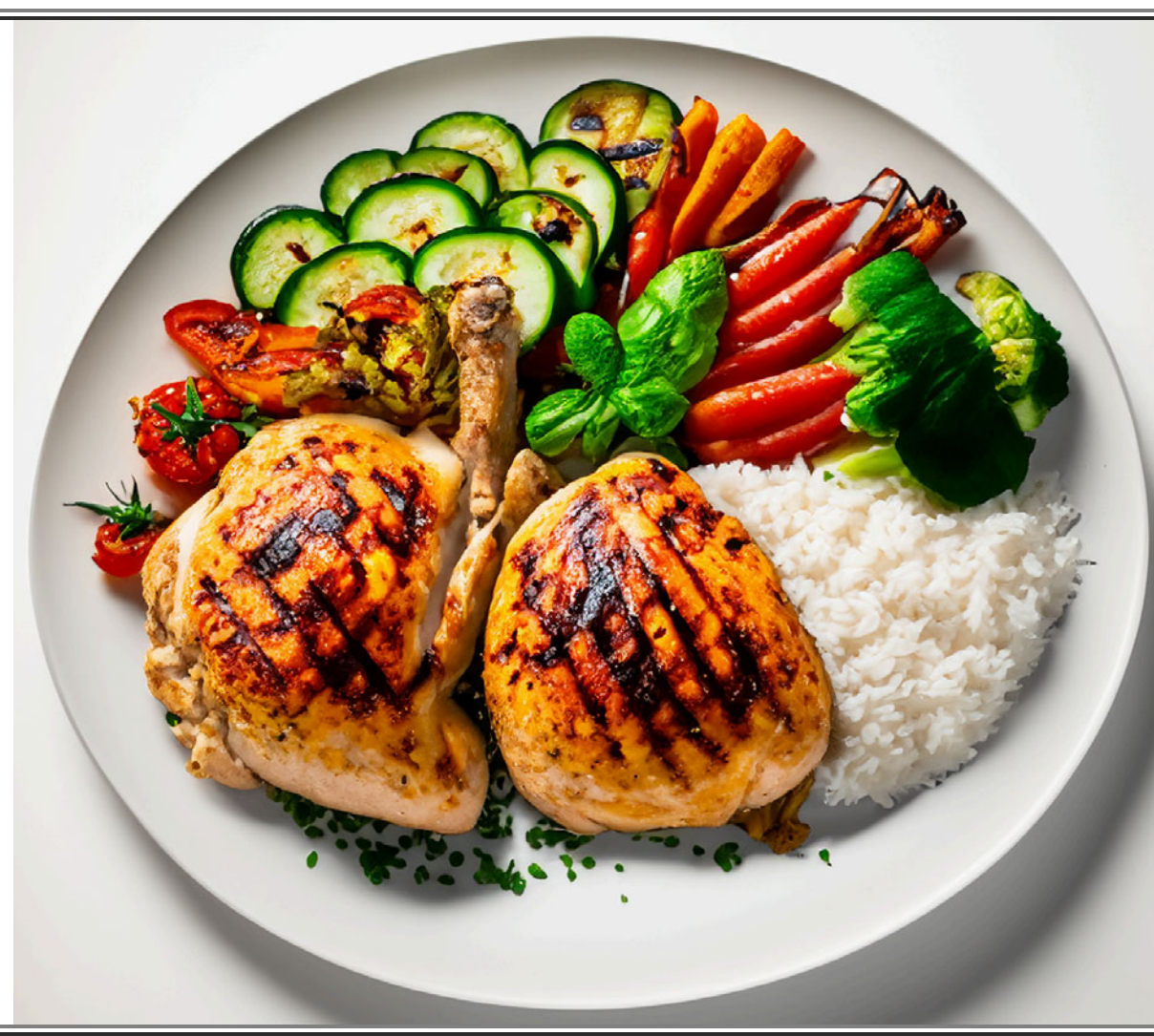

In your view, how do the two products on the top differ from the one on the bottom? Please include both the top and bottom products in your response.

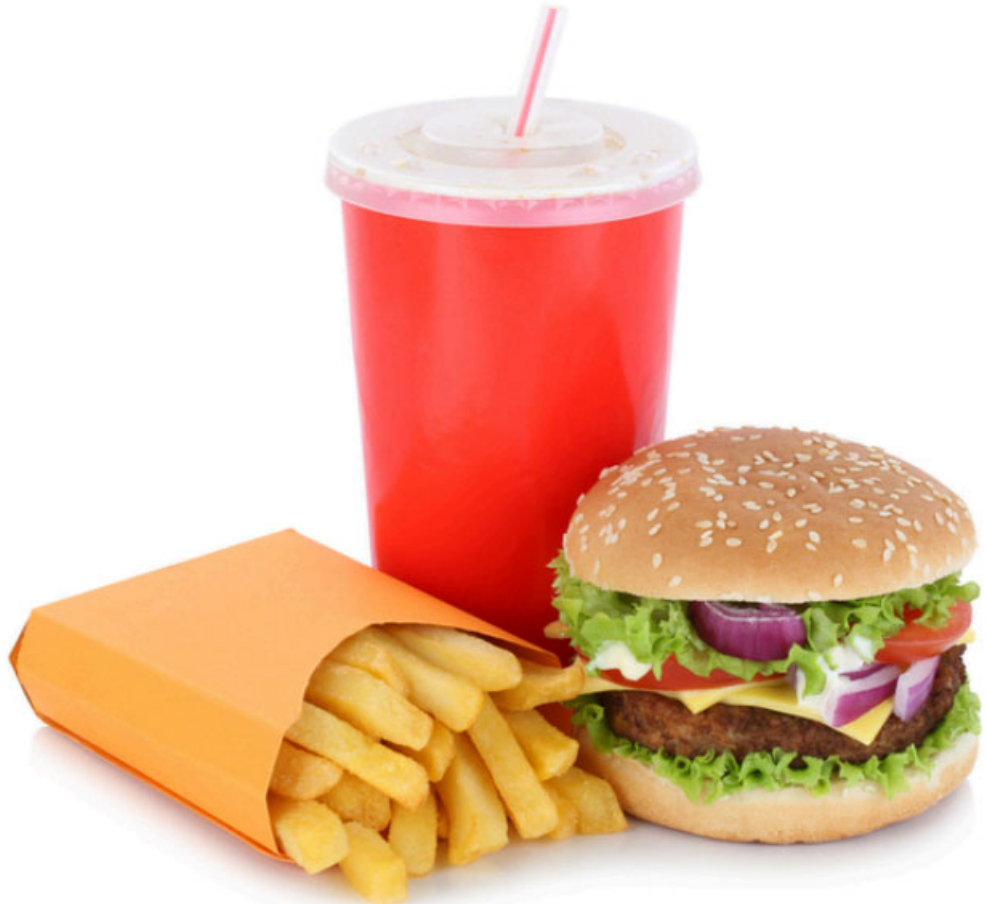

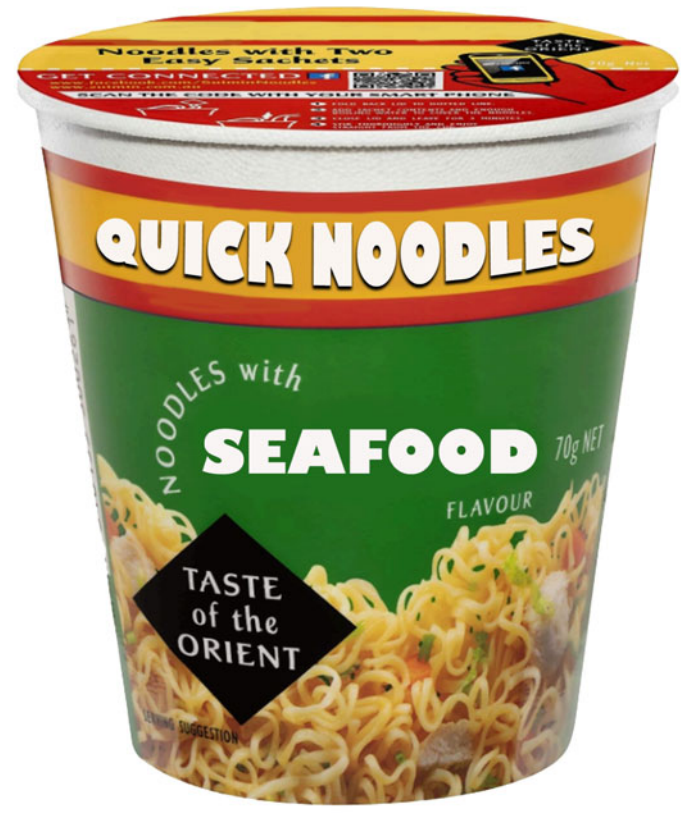

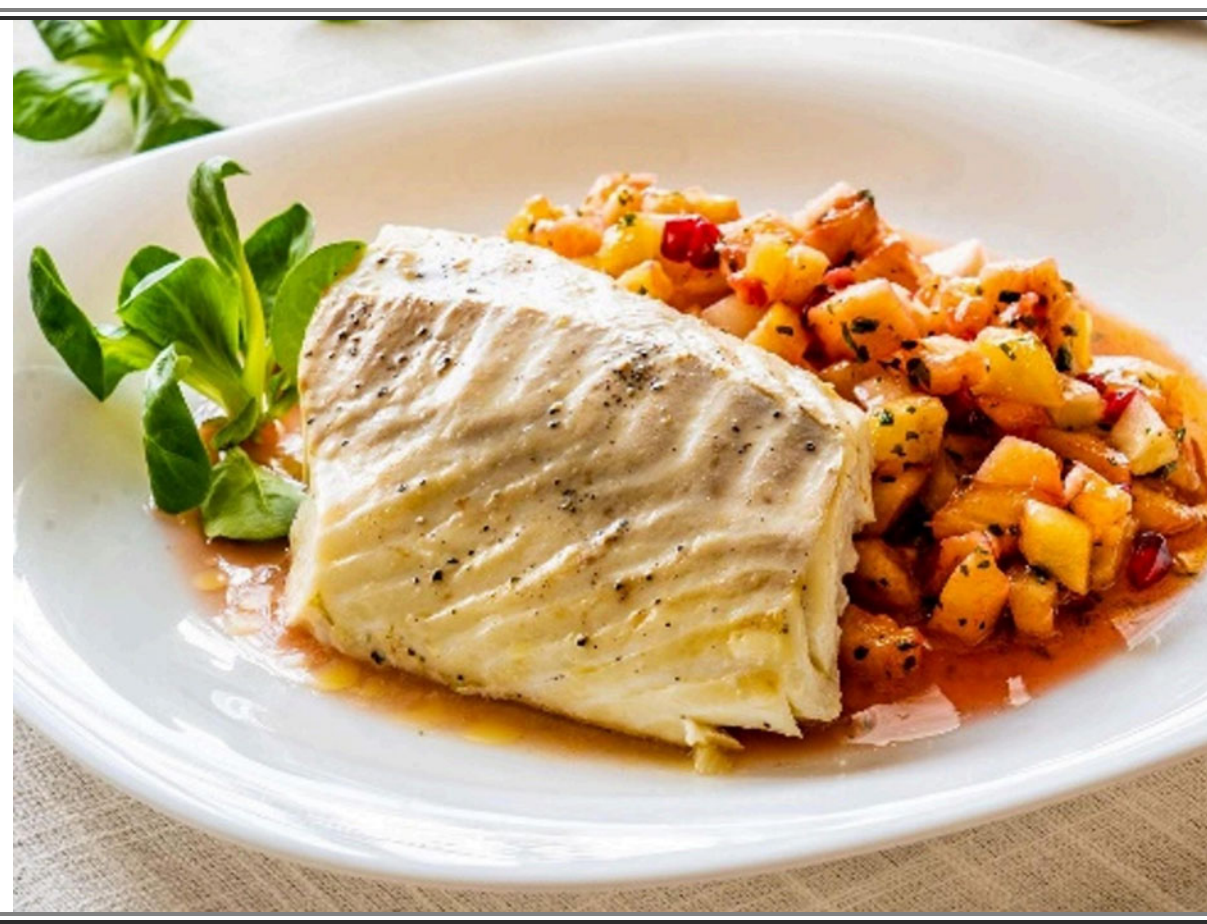

Overall, how healthy is this food product? Please move the bar under the image on a scale from 0 to 10, where 0 = Not at all healthy, and 10 = Extremely healthy.

	Not healthy at all							Extremely healthy				Don't know
	0	1	2	3	4	5	6	7	8	9	10	
Apple												☐

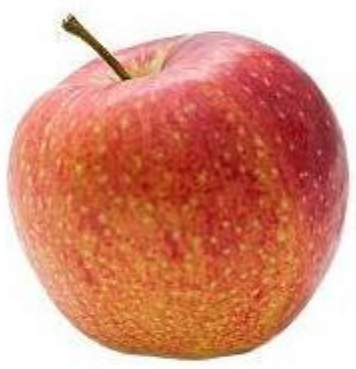

	Not healthy at all							Extremely healthy				Don’t know
	0	1	2	3	4	5	6	7	8	9	10	
Apple Fruit Drink												☐

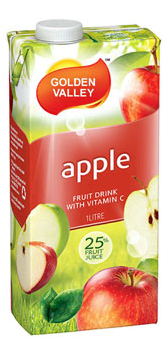

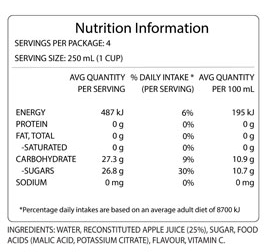

Nutrition Information

SERVINGS PER PACKAGE: 4

SERVING SIZE: 250 mL (1 CUP)

	AVG QUANTITY PER SERVING	% DAILY INTAKE * (PER SERVING)	AVG QUANTITY PER 100 mL
ENERGY	487 kJ	6%	195 kJ
PROTEIN	0 g	0%	0 g
FAT, TOTAL	0 g	0%	0 g
-SATURATED	0 g	0%	0 g
CARBOHYDRATE	27.3 g	9%	10.9 g
-SUGARS	26.8 g	30%	10.7 g
SODIUM	0 mg	0%	0 mg

*Percentage daily intakes are based on an average adult diet of 8700 kJ

INGREDIENTS: WATER, RECONSTITUTED APPLE JUICE (25%), SUGAR, FOOD ACIDS (MALIC ACID, POTASSIUM CITRATE), FLAVOUR, VITAMIN C.

	Not healthy at all							Extremely healthy				Don’t know
	0	1	2	3	4	5	6	7	8	9	10	
Breakfast Cereal Bar												☐

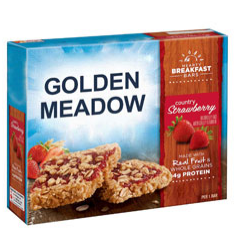

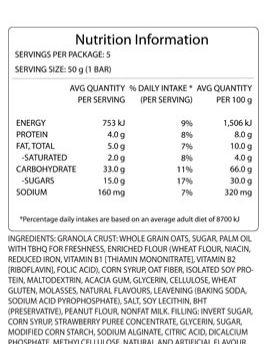

Nutrition Information

SERVINGS PER PACKAGE: 5

SERVING SIZE: 50 g (1 BAR)

	AVG QUANTITY PER SERVING	% DAILY INTAKE * (PER SERVING)	AVG QUANTITY PER 100 g
ENERGY	753 kJ	9%	1,506 kJ
PROTEIN	4.0 g	8%	8.0 g
FAT, TOTAL	5.0 g	7%	10.0 g
-SATURATED	2.0 g	8%	4.0 g
CARBOHYDRATE	33.0 g	11%	66.0 g
-SUGARS	15.0 g	17%	30.0 g
SODIUM	160 mg	7%	320 mg

*Percentage daily intakes are based on an average adult diet of 8700 kJ

INGREDIENTS: GRANOLA CRUST: WHOLE GRAIN OATS, SUGAR, PALM OIL WITH TBHQ FOR FRESHNESS, ENRICHED FLOUR (WHEAT FLOUR, NIACIN, REDUCED IRON, VITAMIN B1 [THIAMIN MONONITRATE], VITAMIN B2 [RIBOFLAVIN], FOLIC ACID), CORN SYRUP, OAT FIBER, ISOLATED SOY PROTEIN, MALTODEXTRIN, ACACIA GUM, GLYCERIN, CELLULOSE, WHEAT GLUTEN, MOLASSES, NATURAL FLAVOURS, LEAVENING (BAKING SODA, SODIUM ACID PYROPHOSPHATE), SALT, SOY LECITHIN, BHT (PRESERVATIVE), PEANUT FLOUR, NONFAT MILK. FILLING: INVERT SUGAR, CORN SYRUP, STRAWBERRY PUREE CONCENTRATE, GLYCERIN, SUGAR, MODIFIED CORN STARCH, SODIUM ALGINATE, CITRIC ACID, DICALCIUM PHOSPHATE, METHYLCELLULOSE, NATURAL AND ARTIFICIAL FLAVOUR, CARAMEL COLOUR, MALIC ACID, RED 40.

	Not healthy at all							Extremely healthy				Don't know
	0	1	2	3	4	5	6	7	8	9	10	
Breakfast Oats 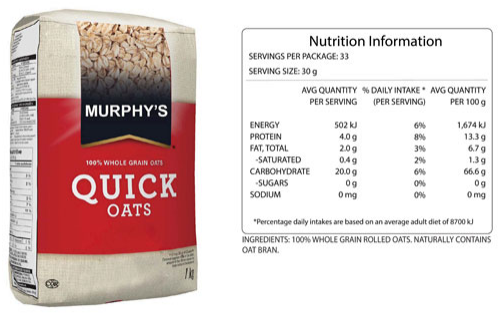												☐

	Not healthy at all							Extremely healthy				Don't know
	0	1	2	3	4	5	6	7	8	9	10	
Milk 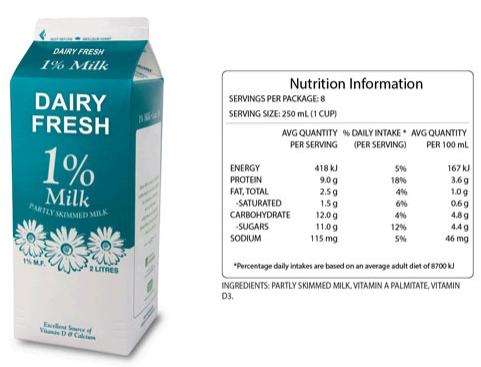												☐

	Not healthy at all							Extremely healthy				Don't know
	0	1	2	3	4	5	6	7	8	9	10	
Cheese Slices 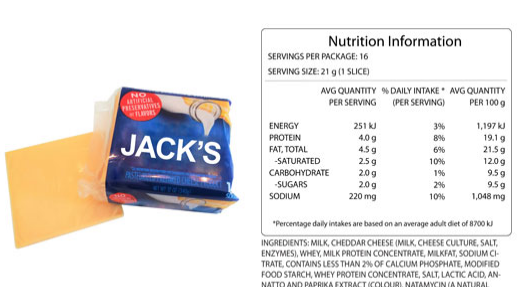												☐

	Not healthy at all							Extremely healthy				Don't know
	0	1	2	3	4	5	6	7	8	9	10	
Chicken Breast 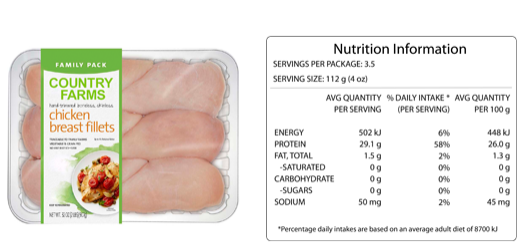												☐

	Not healthy at all							Extremely healthy				Don't know
	0	1	2	3	4	5	6	7	8	9	10	
Chicken Nuggets 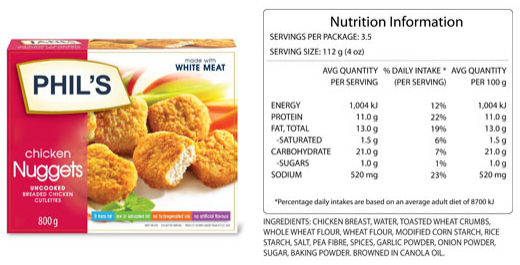												☐

Is there anything else you would like to tell us about ultra-processed foods?

Prior to today, had you heard about ultra-processed foods?

- ◯ Yes
- ◯ No

How do you feel about having a quick way to spot ultra-processed foods on packaging? For example, this could be a logo or other mark on the package.

Block 5

Please click the next button to submit your responses.

Would you like to learn more about ultra-processed foods?

Watch the 1 minute video below!

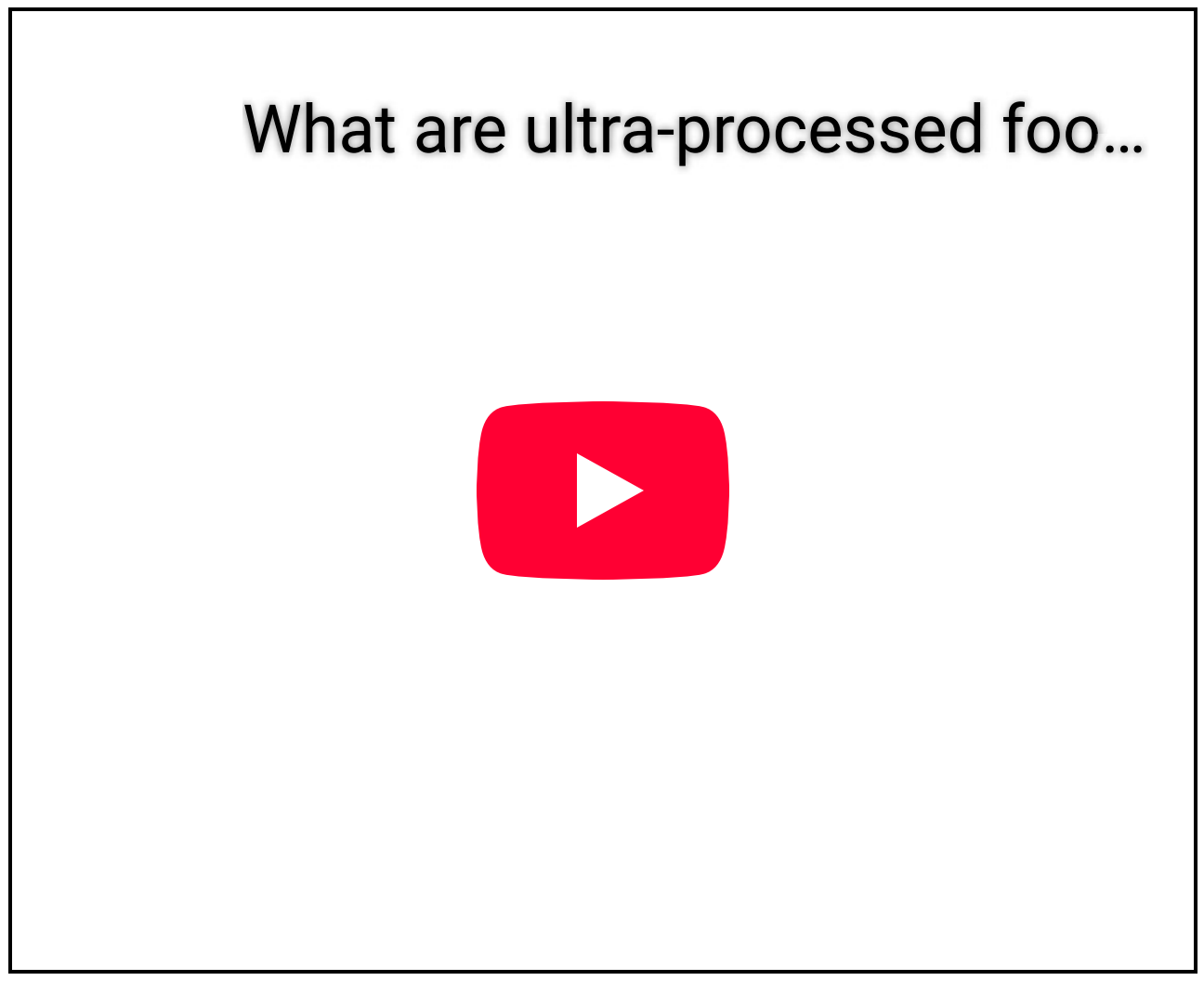

Deakin University CRICOS Provider Code 00113B.

Powered by Qualtrics

Supplementary file 3. Good Reporting of A Mixed Methods Study (GRAMMS) checklist

**Guideline**	**Section: page**
**Describe the justification for using a mixed methods approach to the research question**	Methods – Study design and participants; pg. 9
**Describe the design in terms of the purpose, priority and sequence of methods**	Methods – Study design and participants; pg. 9
**Describe each method in terms of sampling, data collection and analysis**	Methods – Study measures; pg. 9-11<br>Methods – Data analysis: pg. 11-14
**Describe where integration has occurred, how it has occurred and who has participated in it**	Methods – Data analysis: pg. 14
**Describe any limitation of one method associated with the present of the other method**	Discussion - Strengths and Limitations; pg. 26
**Describe any insights gained from mixing or integrating methods**	Discussion; pg. 26

Reference:

O'Cathain A, Murphy E, Nicholl J. The quality of mixed methods studies in health services research. *J Health Serv Res Policy*. 2008;13: 92-98.

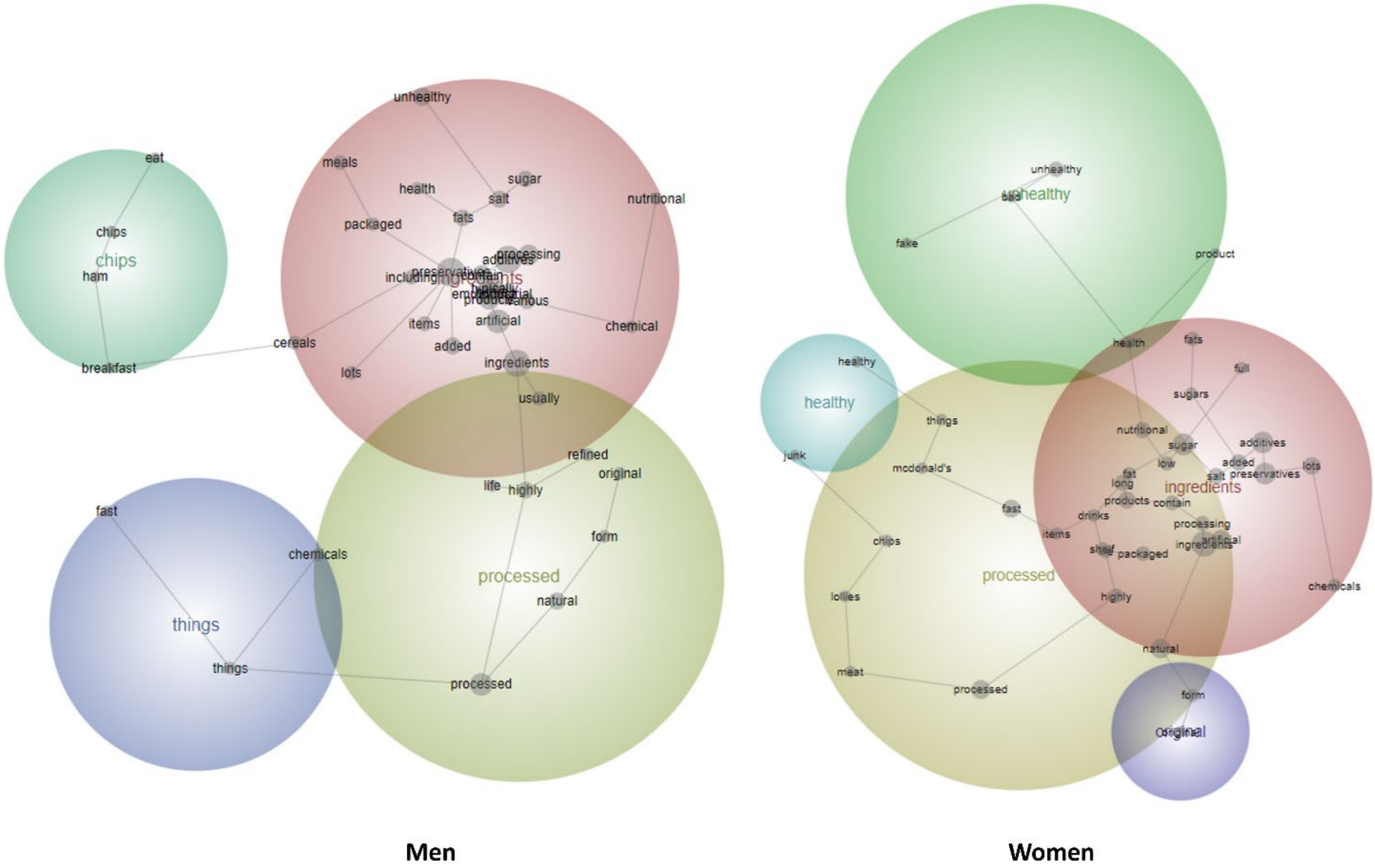

Supplementary file 4. Concept maps for participant descriptions of ultra-processed foods stratified by gender. In order of decreasing dominance themes for men were: “ingredients”, “processed”, “things”, “chips”. In order of decreasing dominance themes for women were: “ingredients”, “processed”, “unhealthy”, “healthy”, “original”.

Supplementary file 5. Classification of ultra-processed food according to respondents (n=1,086)

Food item	Correct (N, %)	Incorrect (N, %)
**Total**	**692 (65.5)**	**364 (34.5)**
**Ultra-processed food**	**480 (45.5)**	**576 (54.5)**
Chicken nuggets	788 (72.6)	298 (27.4)
Fast food burger	754 (69.4)	332 (30.6)
Instant noodles	747 (68.8)	339 (31.2)
Ready meal lasagne	672 (61.9)	414 (38.1)
Chocolate bar	478 (44.0)	608 (56.0)
Flavoured water	439 (40.4)	647 (59.6)
Cereal bar	353 (32.5)	733 (67.5)
Bolognese pasta sauce	284 (26.1)	802 (73.9)
Flavoured yoghurt	240 (22.1)	846 (77.9)
Multi-grain packaged bread	184 (16.9)	902 (83.1)
**Non-ultra-processed food**	**903 (85.5)**	**153 (14.5)**
Plain yoghurt	1,008 (92.8)	78 (7.2)
Pasta	981 (90.3)	105 (9.7)
Full cream milk	973 (89.6)	113 (10.4)
Canned corn	950 (87.5)	136 (12.5)
Muesli	938 (86.4)	148 (13.6)
Canned fish	928 (85.5)	158 (14.5)
100% Fruit juice	922 (84.9)	164 (15.1)
Cheese block	896 (82.5)	190 (17.5)
Bakery white bread	851 (78.4)	235 (21.6)
Tortilla strips corn	835 (76.9)	251 (23.1)

Supplementary file 6. Food Processing Knowledge Score by food category using continuous (main analysis) and binary scoring (n=1,012).

Category	FoodProK continuous score*	FoodProK binary score*
	Mean (SD)	Mean (SD)
Fruit	5.31 ± 2.72	0.95 ± 0.22
Grain	3.85 ± 2.92	0.86 ± 0.35
Dairy	2.62 ± 2.94	0.76 ± 0.43
Meat	5.23 ± 2.94	0.91 ± 0.28
**FoodProK score**	**17.1 ± 8.53**	**3.48 ± 0.84**

*Continuous score ranges from -40 to +40 is calculated based on difference between healthiness ratings attributes to UPFs vs non-UPFs. Binary score ranges from 0 to 4 and is calculated as 0/1 per category if a higher healthiness rating is given to a non-UPF (yes/no).

Supplementary file 7. Socio-demographic correlates of the number of items correctly identified as UPFs and Food Processing Knowledge (FoodProK) scores.

	Number of items correctly identified as UPF*			FoodProK		
	β	95 % CI	P-value	β	95 % CI	P-value**
**Gender**						
Men vs. Women	-0.74	-1.09, -0.38	**<0.001**	-3.78	-5.04, -2.51	**<0.001**
Non-binary/other vs. Women	0.06	-1.33, 1.46	1.00	1.96	-3.44, 7.36	1.00
Non-binary/other vs. Men	0.80	-0.60, 2.20	0.51	5.74	0.33, 11.15	**0.03**
**Age (years)**						
30-39 vs. 20-29	-0.003	-0.55, 0.54	1.00	1.37	-0.61, 3.35	0.41
40-49 vs. 20-29	0.14	-0.42, 0.69	1.00	2.15	0.12, 4.18	**0.03**
50-64 vs. 20-29	-0.12	-0.66. 0.43	1.00	1.96	-0.03, 3.94	0.06
40-49 vs. 30-39	0.14	-0.41, 0.69	1.00	0.78	-1.23, 2.79	1.00
50-64 vs. 30-39	-0.11	-0.66, 0.43	1.00	0.59	-1.38, 2.56	1.00
50-64 vs. 40-49	-0.26	-0.81, 0.30	1.00	-0.19	-2.21, 1.82	1.00
**Education**						
Year 11 or lower vs. Year 12	-0.42	-0.80, -0.03	**0.03**	-0.11	-1.52, 1.30	0.88
**Area-level disadvantage**						
Q2 vs. Q1	-0.00	-0.69, 0.69	1.00	-0.05	-2.57, 2.48	1.00
Q3 vs. Q1	-0.02	-0.67, 0.62	1.00	-0.29	-2.66, 2.08	1.00
Q4 vs. Q1	0.20	-0.44, 0.85	1.00	0.85	-1.53, 3.23	1.00
Q5 vs. Q1	0.54	-0.15, 1.23	0.28	1.37	-1.17, 3.91	1.00
Q3 vs. Q2	-0.02	-0.67, 0.62	1.00	-0.25	-2.61, 2.11	1.00
Q4 vs. Q2	0.20	-0.45, 0.86	1.00	0.89	-1.48, 3.26	1.00
Q5 vs. Q2	0.54	-0.15, 1.24	0.29	1.41	-1.12, 3.95	1.00
Q4 vs. Q3	0.23	-0.37, 0.83	1.00	1.14	-1.06, 3.34	1.00
Q5 vs. Q3	0.56	-0.08, 1.21	0.14	1.66	-0.71, 4.04	0.49
Q5 vs. Q4	0.33	-0.32, 0.98	1.00	0.52	-1.86, 2.91	1.00
**Country of birth**						
Other vs. Australia	-0.31	-0.65, 0.04	0.08	-0.82	-2.08, 0.43	0.19
**Language spoken**						
Other vs. English	-0.57	-1.18, 0.04	0.06	-2.47	-4.67, -0.26	**0.03**
**Aboriginal, Torres Strait Islander or both**						
Yes vs. No	-0.82	-1.59, -0.06	**0.035**	-5.78	-8.63, -2.92	**<0.001**
**State**						
ACT vs NSW	0.47	-1.50, 2.43	1.00	-0.66	-8.22, 6.91	1.00
VIC vs NSW	0.39	-0.21, 0.99	1.00	0.20	-2.01, 2.41	1.00
QLD vs NSW	0.11	-0.54, 0.76	1.00	-1.21	-3.61, 1.2	1.00
SA vs NSW	-0.01	-0.88, 0.87	1.00	-0.26	-3.47, 2.95	1.00
WA vs NSW	0.15	-0.75, 1.05	1.00	-0.29	-3.53, 2.94	1.00
TAS vs NSW	1.60	0.18, 3.02	**0.01**	-0.96	-6.23, 4.32	1.00
NT vs NSW	1.07	-2.68, 4.82	1.00	2.90	-10.55, 16.36	1.00
VIC vs ACT	-0.07	-2.05, 1.60	1.00	0.86	-6.74, 8.45	1.00
QLD vs ACT	-0.36	-2.34, 1.63	1.00	-0.55	-8.2, 7.1	1.00
SA vs ACT	-0.48	-2.55, 1.60	1.00	0.40	-7.54, 8.34	1.00
WA vs ACT	-0.32	-2.40, 1.74	1.00	0.37	-7.59, 8.32	1.00
TAS vs ACT	1.13	-1.22, 3.45	1.00	-0.30	-9.27, 8.68	1.00
NT vs ACT	0.6	-3.59, 4.79	1.00	3.56	-11.73, 18.85	1.00
QLD vs VIC	-0.28	-0.96, 0.39	1.00	-1.41	-3.89, 1.08	1.00
SA vs VIC	-0.40	-1.29, 0.49	1.00	-0.46	-3.73, 2.81	1.00
WA vs VIC	-0.24	-1.16, 0.67	1.00	-0.49	-3.79, 2.8	1.00
TAS vs VIC	1.21	-0.22, 2.63	0.24	-1.16	-6.47, 4.15	1.00
NT vs VIC	0.67	-3.08, 4.42	1.00	2.70	-10.77, 16.17	1.00
SA vs QLD	-0.12	-1.05, 0.81	1.00	0.95	-2.46, 4.35	1.00
WA vs QLD	0.04	-0.91, 0.98	1.00	0.91	-2.52, 4.34	1.00
TAS vs QLD	1.49	0.04, 2.94	**0.04**	0.25	-5.14, 5.64	1.00
NT vs QLD	0.96	-2.80, 4.72	1.00	4.11	-9.4, 17.61	1.00
WA vs SA	0.16	-0.96, 1.27	1.00	-0.03	-4.06, 4	1.00
TAS vs SA	1.61	0.04, 3.18	**0.04**	-0.70	-6.49, 5.1	1.00
NT vs SA	1.08	-2.72, 4.88	1.00	3.16	-10.51, 16.83	1.00
TAS vs WA	1.45	-0.12, 3.03	0.11	-0.66	-6.48, 5.15	1.00
NT vs WA	0.92	-2.89, 4.73	1.00	3.19	-10.48, 16.87	1.00
NT vs TAS	-0.53	-4.50, 3.43	1.00	3.86	-10.44, 18.15	1.00

**Rurality**						
Other vs. Major city	0.04	-0.31,0.39	0.82	0.69	-0.59, 1.97	0.29
**Health background**						
Yes vs. No	-0.45	-0.86, -0.05	**0.03**	-2.53	-4.00, -1.07	**0.001**
**Prior awareness about UPFs**						
Yes vs. No	1.01	0.72, 1.30	**<0.001**	2.08	0.98, 3.17	**<0.001**

Notes: CI Confidence Intervals. NSW: New South Wales; VIC: Victoria; QLD: Queensland; SA: South Australia; WA: Western Australia; TAS: Tasmania; ACT: Australian Capital Territory; NT: Northern Territory

*Ranges from 0-20, includes correctly classifying both UPF (n=10) and non-UPF (n=10) items.

**Variables are significant if $p < 0.05$ after post hoc adjustment using Bonferroni procedure.

Supplementary file 8. Leximancer identified themes and concepts from participant descriptions of ultra-processed foods (n=1,086)

Theme	Hits	Concept	Count	Relevance
Ingredients	490	ingredients	107	62%
		preservatives	106	61%
		additives	98	57%
		artificial	69	40%
		sugar	66	38%
		lots	59	34%
		added	44	25%
		chemicals	43	25%
		processing	42	24%
		products	39	23%
		fats	37	21%
		nutritional	37	21%
		packaged	36	21%
		salt	35	20%
		contain	30	17%
		full	25	14%
		processes	25	14%
		long	24	14%
		items	24	14%
		meals	20	12%
		shelf	20	12%
		nutrients	18	10%
		typically	17	10%
		drinks	15	9%
		instant	14	8%
		taste	14	8%
Processed	320	processed	173	100%
		natural	103	60%
		meat	47	27%
		highly	42	24%
		form	31	18%
		original	27	16%
Fast	194	fast	66	38%
		things	37	21%
		chips	34	20%
		junk	30	17%
		McDonald's	28	16%
		ready	22	13%
		cereals	19	11%
		biscuits	13	8%
Unhealthy	182	unhealthy	121	70%
		healthy	33	19%
		health	23	13%
		bad	23	13%

Theme names were automatically assigned by Leximancer based on the most frequently occurring concept under a given theme. Hits refers to the number of times a segment of text has been captured under a theme. Count is the number of times a concept appeared in participant responses. Relevance refers to the frequency of text segments that contain the concept, proportionate to the frequency of the most dominant concept.
